# Transcriptome Sequencing Reveals Wide Expression Reprogramming of Basal and Unknown Genes in *Leptospira biflexa* Biofilms

**DOI:** 10.1128/mSphere.00042-16

**Published:** 2016-04-06

**Authors:** Gregorio Iraola, Lucía Spangenberg, Bruno Lopes Bastos, Martín Graña, Larissa Vasconcelos, Áurea Almeida, Gonzalo Greif, Carlos Robello, Paula Ristow, Hugo Naya

**Affiliations:** a Unidad de Bioinformática, Institut Pasteur, Montevideo, Montevideo, Uruguay; b Sección Genética Evolutiva, Facultad de Ciencias, Montevideo, Uruguay; c Laboratório de Bacteriologia e Saúde, Instituto de Biologia, Universidade Federal da Bahia, Salvador, Bahia, Brazil; d Unidad de Biología Molecular, Institut Pasteur, Montevideo, Montevideo, Uruguay; e Departamento de Bioquímica, Facultad de Medicina, Montevideo, Uruguay; f Departamento de Producción Animal y Pasturas, Facultad de Agronomía, Montevideo, Uruguay; University of Wisconsin

**Keywords:** *Leptospira*, RNA sequencing, biofilms, gene expression, transcriptomics

## Abstract

The genus *Leptospira* is composed of pathogenic and saprophytic spirochetes. Pathogenic *Leptospira* is the etiological agent of leptospirosis, a globally spread neglected disease. A key ecological feature of some pathogenic species is their ability to survive both within and outside the host. For most leptospires, the ability to persist outside the host is associated with biofilm formation, a most important bacterial strategy to face and overcome hostile environmental conditions. The architecture and biochemistry of leptospiral biofilms are rather well understood; however, the genetic program underpinning biofilm formation remains mostly unknown. In this work, we used the saprophyte *Leptospira biflexa* as a model organism to assess over- and underrepresented transcripts during the biofilm state, using transcriptome sequencing (RNA-seq) technology. Our results showed that some basal biological processes like DNA replication and cell division are downregulated in the mature biofilm. Additionally, we identified significant expression reprogramming for genes involved in motility, sugar/lipid metabolism, and iron scavenging, as well as for outer membrane-encoding genes. A careful manual annotation process allowed us to assign molecular functions to many previously uncharacterized genes that are probably involved in biofilm metabolism. We also provided evidence for the presence of small regulatory RNAs in this species. Finally, coexpression networks were reconstructed to pinpoint functionally related gene clusters that may explain how biofilm maintenance is regulated. Beyond elucidating some genetic aspects of biofilm formation, this work reveals a number of pathways whose functional dissection may impact our understanding of leptospiral biology, in particular how these organisms adapt to environmental changes.

**IMPORTANCE** In this work, we describe the first transcriptome based on RNA-seq technology focused on studying transcriptional changes associated with biofilm growth in a member of the genus *Leptospira*. As many pathogenic species of this genus can survive inside the host but also persist in environmental water, mostly forming biofilms, identifying the molecular basis of this capacity can impact the understanding of how leptospires are able to fulfill a complete life cycle that alternates between adaptation to the host and adaptation to hostile external environmental conditions. We identified several genes and regulatory networks that can be the kickoff for deepening understanding of the molecular mechanisms involving bacterial persistence via biofilm formation; understanding this is important for the future development of tools for controlling leptospirosis.

## Abstract (precis)

In this work, we describe the first transcriptome based on RNA-seq technology focused on studying transcriptional changes associated with biofilm growth in a member of the genus *Leptospira*. As many pathogenic species of this genus can survive inside the host but also persist in environmental water, mostly forming biofilms, identifying the molecular basis of this capacity can impact the understanding of how leptospires are able to fulfill a complete life cycle that alternates between adaptation to the host and adaptation to hostile external environmental conditions. We identified several genes and regulatory networks that can be the kickoff for deepening understanding of the molecular mechanisms involving bacterial persistence via biofilm formation; understanding this is important for the future development of tools for controlling leptospirosis.

## INTRODUCTION

Leptospirosis is a neglected disease caused by infections with bacteria belonging to the genus *Leptospira*. This worldwide-distributed zoonotic disease is relevant for animal and human health, with more than 500,000 documented cases per year and particularly high incidences in developing countries (1). The genus *Leptospira* contains both saprophytic and pathogenic species differing in their capacities for surviving and colonizing different environments and hosts, ranging from soil and water to mammalian tissues during infection (2). *Leptospira* species have been historically classified into three groups according to their pathogenic potential: pathogens, intermediate pathogens, and saprophytes (3). The advent of genomics allowed researchers to identify 21 species that are phylogenetically correlated with the previously referenced groups. Recently, a revision of leptospiral taxonomy based on genomics proposed the following classification: group I (previously known as pathogens) comprises 9 species that include *Leptospira interrogans*, *Leptospira kirschneri*, and *Leptospira noguchii*, which cause the most severe cases of leptospirosis. Group II (intermediate pathogens) includes 5 species that predominantly cause milder cases of leptospirosis. Group III (saprophytes) is composed of nonpathogenic, free-living environmental leptospires like *Leptospira biflexa* (4). This classification is adopted here.

The increasing availability of whole-genome sequences for species belonging to the three groups has enabled the identification of genome-wide evolutionary processes involved in the transition from a nonpathogenic and free-living form to a pathogenic and host-adapted lifestyle. For example, comparative genomics have revealed that *L. interrogans* (group I) has a larger genome than *L. biflexa* (group III), probably reflecting additional genetic features required for survival in both soil/water and mammalian hosts (2). Importantly, the fact that *L. interrogans* retained the ability to survive in the environment as a free-living organism directly impacts the ecology and epidemiology of leptospirosis, since these organisms are capable of colonizing and multiplying inside the renal tubules of chronically infected reservoir species, disseminating in the urine, and contaminating soil and water. Humans and other mammals are then infected by direct contact with animal fluids or contaminated water (5).

As stated before, survival outside the host is a key aspect of leptospiral ecology and hence for pathogenesis. Like most prokaryotes, *Leptospira* can form biofilms to survive when cells are exposed to the outside environment. These matrix-confined bacterial populations protect single cells from adverse conditions, favoring persistence and transmission of infectious diseases (6). The transition between planktonic and biofilm phenotypes occurs as a response to various environmental signals. It involves producing and assembling components of an extracellular matrix (ECM), cell migration, adhesion, and aggregation, among other processes, which are regulated by the expression of specific genes. In this sense, the consolidation of whole-RNA sequencing (RNA-seq) as the gold standard method for evidencing transcription reprogramming through biological conditions (7) has enabled the study of differential gene expression associated with biofilm formation in many microorganisms (8–10). Nonetheless, and despite biofilm formation having been described *in vitro* for pathogenic and saprophytic leptospires (11), and also observed *in vivo* (12), a genome-wide transcriptomic analysis is still lacking for *Leptospira* species in the context of biofilm formation.

From an ecological point of view, leptospiral pathogenesis can be linked with biofilms particularly in species that can complete a life cycle within and outside the host, so elucidating the genetic basis of biofilm formation can provide useful tools for genetic manipulation, drug design, and vaccine development, which should directly impact disease handling and could substantially improve the design of preventive schemes. In this work, we selected *L. biflexa* serovar Patoc strain Patoc I (Paris) as a model organism to compare the global gene expression profiles between biofilm grown on abiotic surfaces and planktonic cells, using RNA-seq. Our results indicate that biofilm growth requires the extensive reprogramming of transcription patterns along the three replicons of *L. biflexa* and involves many regulatory networks like c-di-GMP signaling, anti-anti-sigma factors, and canonical two-component systems that control basal functions, like DNA metabolism and replication, as well as more specific functions like cell motility or lipid and sugar metabolisms.

## RESULTS AND DISCUSSION

### Transcriptomic overview of *L. biflexa*.

The whole transcriptome was sequenced for 12 cultures of *L. biflexa* Patoc strain Patoc I harvested at 48 h and 120 h under both biofilm and planktonic culture conditions, using biological triplicates. The average yield of reads per sample was ~4 million, indicating a sufficient amount of data for performing differential expression analyses (13). Out of the total number of reads sequenced per sample, ~99% mapped against the reference *L. biflexa* Patoc strain Patoc I genome (see Table S4 in the supplemental material). The hierarchical clustering of samples using normalized read counts was consistent with harvesting time and culture condition, upon removal of three discordant samples, not included in subsequent analyses. For differential gene expression analyses, all possible comparisons of time (mature or late) and culture conditions (planktonic or biofilm) were carried out using the 9 consistently clustering samples (see Fig. S1). The numbers of down- and upregulated genes (false discovery rate [FDR], <1e−2) for each comparison are shown in Table 1. The most relevant information for identifying functional changes in gene expression came from comparing mature biofilm with mature planktonic cells. The full list of differentially expressed genes in each comparison is presented in Table S5.

10.1128/mSphere.00042-16.1 Figure S1  Multidimensional scaling plot for samples considered in differential expression analysis. Download Figure S1, PDF file, 0.1 MB. Copyright © 2016 Iraola et al. 2016 Iraola et al. This content is distributed under the terms of the Creative Commons Attribution 4.0 International license.

**TABLE 1:** Number of differentially expressed genes detected in each comparison at an FDR of <1e−2

Comparison	No. of genes		
	Upregulated	Downregulated	Total
Biofilm vs planktonic growth, 48 h	121	198	319
Biofilm vs planktonic growth, 120 h	184	117	301
Biofilm, 48 h vs 120 h	151	172	323
Planktonic growth, 48 h vs 120 h	184	240	424

The reference genome of *L. biflexa* Patoc strain Patoc I contains a total of 3,771 predicted genes distributed within three replicons: chromosome I (CI), chromosome II (CII), and a 74-kb plasmid (P74) with chromosome-like features (2). Transcriptional activity was detected in 3,762 genes in at least one sample, indicating that the vast majority (99%) of predicted genes of *L. biflexa* were transcriptionally active. Further analysis of 9 annotated genes that remained silent in all samples evidenced the presence of small hypothetical proteins and RNA coding genes. Most notably, two pairs of the MerR/MerT system were identified among these silent genes. This system belongs to the *mer* operon, involved in the resistance to high concentrations of metals like mercury and aluminum (14, 15). These are the two unique copies of *merR* and *merT* genes in the genome of *L. biflexa*, interspaced by ~36 kb in CI. The two gene pairs show the same arrangement and are surrounded by a number of hypothetical genes. However, one pair is closer to putative plasmid-like genes, suggesting horizontal acquisition and/or gene duplication. Dissecting why this system remains totally silent in *L. biflexa* will require further investigation.

### Expression through replicons.

**FIG 1:**
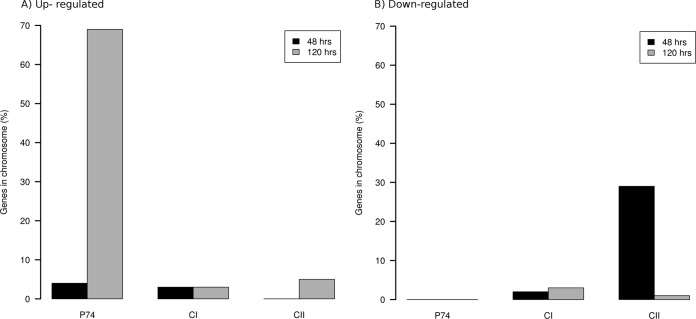
Number of genes (percentage) in each replicon with differential expression. The bar graphs show the percentage of each *L. biflexa* replicon (measured as number of differentially expressed genes over total number of genes in the replicon) that were upregulated (A) and downregulated (B).

The number of differentially expressed genes varied when considering mature (48-h) or late (120-h) biofilms and also when considering gene location (CI, CII, or P74). For instance, in mature biofilms, upregulated genes came only from CI and P74 (Fig. 1A), while downregulated genes were exclusively found in CII (29% of genes carried in this chromosome) (Fig. 1B). These results suggest that replicons in *L. biflexa* fulfill different tasks during biofilm formation and that up- and downregulation are appreciably compartmentalized throughout this growing condition. This notion is reinforced when examining late biofilms (120 h). During this stage, both up- and downregulated genes were almost equally distributed between CI and CII (Fig. 1B); however, around 68% of genes present in P74 were upregulated. It is not clear whether P74 behaves as a chromosome or as an extrachromosomal element, even if some essential survival genes (like *recBCD*) are located in that replicon. These genes are found in CI in other pathogenic species like *L. interrogans*, and altering their sequences has been linked to lower viability in other bacterial species, suggesting that P74 is essential for survival of *L. biflexa* (2). Our findings support this hypothesis, considering the pervasive upregulation of most genes contained in P74, and also suggest a previously unknown role of this replicon in the late stages of biofilm, which are characterized by cell recycling, disaggregation, and death.

### Replication and cell growth.

The capacity to persist under resource-limiting conditions (like environmental water in the case of *Leptospira*) is a major advantage conferred by biofilms. This ability is based on an altruistic behavior that relies on maximizing the biomass formed per amount of resources used (16), meaning that single cells can reduce their growth rate and resource consumption for the benefit of the whole population (biofilm). In the context of this hypothesis, we found that key genes involved in DNA replication and cell division were differentially expressed*.* In particular, the gene coding for the chromosomal replication initiator protein DnaA (*LEPBI_I0001*) was downregulated during mature biofilm, as well as other genes coding for proteins implied in replication, like DNA polymerase III subunits (*LEPBI_I0012*, *LEPBI_I3461*, and *LEPBI_I3479*), chromosome partitioning protein ParB (*LEPBI_I3473* and *LEPBI_II0026*), replication proteins GidA and GidB (*LEPBI_I3477* and *LEPBI_3475*), DNA replication and repair protein RecF (*LEPBI_I0003*), and DNA gyrase GyrB1 (*LEPBI_I0005*). Additionally, we found one putative gene for the virulence-associated protein of unknown function VagC (*LEPBI_I2249*) to be upregulated during mature biofilm. This gene presented homology to *mazE*, belonging to the MazF-MazE toxin/antitoxin system, and was also placed next to a hypothetical protein-encoding gene (*LEPBI_I2248*) with homology to MazF. This system is involved in cell growth regulation and programmed cell death during resource shortages in *Escherichia coli* (17), and although the cognate MazF homolog was not transcriptionally altered, just altering the levels of MazE is enough to regulate cell growth (18). Moreover, we found three additional downregulated genes in mature and late biofilms that code for HepA, Fis, and a pyrroloquinoline quinone (*LEPBI_I3440*, *LEPBI_I0011*, and *LEPBI_I3348*, respectively) that have been associated with cell growth control in other bacteria. In particular, HepA and Fis have been identified as overexpressed genes during the fast growth or exponential growth phase (19, 20), while here we found them downregulated, in accordance with the notion of low replication and cell growth in *L. biflexa* biofilms.

### Lack of translational motility.

Motility is a central paradigm in bacterial physiology. In *Leptospira*, this mechanism is mainly controlled by two periplasmic flagella, whose opposite rotation provokes a topological change in both cell poles (spiral-hook configuration) that allows translational displacement by a corkscrew movement (21). Switching from motile to nonmotile forms depends on the fine interaction between the flagellar apparatus and chemotactic systems. Using dark-field microscopy, we observed null translational motility in the vast majority of cells in mature and late biofilms. This observation led us to hypothesize that genes involved in determining the spiral-hook configuration needed for translational movement were altered in the biofilm condition. We found two genes encoding homologs of the *pilZ* product (*LEPBI_I0008* and *LEPBI_II0088*) consistently downregulated in mature biofilm. The interaction of PilZ-containing proteins with the flagellar switch-complex proteins FliG and FliM induces counterclockwise motor bias that results in reversal of the flagellar rotation (22). The fact that *LEPBI_I0008* and *LEPBI_II0088* remained downregulated supports our hypothesis that spiral-hook configuration could be prevented by reducing the interaction of these PilZ-containing proteins with FliG and FliM. However, the identification of other PilZ-containing proteins with additional molecular functions, like Alg44, which is involved in exopolysaccharide biosynthesis in *Pseudomonas* (23), points out that our hypothesis needs to be checked in the future by experimental procedures.

The motor switch proteins FliG and FliM also have other interactors that affect flagellar motor bias, such as the signal transducer CheY. This protein presents four annotated paralogs in the genome of *L. biflexa* (*cheY1* to -*4*), but only *cheY1* (*LEPBI_I0917*) showed differential expression (upregulation) in mature biofilm in our analysis. The role of CheY in motility behavior has been studied using recombinant *E. coli* to evaluate *cheY* genes carried by *L. interrogans* (24), where they are also highly redundant (5 paralogs)*.* The overexpression of *cheY* genes from *L. interrogans* in *E. coli* mainly caused swarming inhibition (25). Moreover, we found that *cheR* (*LEPBI_I1764*) was downregulated in mature biofilm. The deletion of this gene in *L. interrogans* resulted in a swarming-defective phenotype (26). Based on these results, we can suggest that the upregulation of *cheY1* and downregulation of *cheR* should be contributing to the lack of translational movement observed in *L. biflexa* biofilms. In addition, leptospires are attached to one another in the biofilm and enclosed by an exopolysaccharidic matrix, which hampers translational motility once biofilm is mature (11).

When considering the structural components of the flagellar filament, it was striking to find that *flaB123* (core flagellar subunits) (*LEPBI_I1589*, *LEPBI_I2133*, and *LEPBI_I2132*, respectively) and *flaA1* (sheath subunit) (*LEPBI_I2335*) were upregulated in mature biofilm, while no additional genes coding for the flagellar apparatus were differentially expressed in any comparison. Leptospires have only two periplasmic flagella, and FlaB is essential for correct assembly and FlaA is required for motility and virulence in *L. interrogans* (27). It is difficult to interpret the possible role that overproduction of flagellar components may have in the context of motility, since leptospiral motility is performed with just two flagellar filaments. One possibility is that FlaA and FlaB have unknown pleiotropic functions for biofilm homeostasis; hence, deepening the study of motility genes in *Leptospira* from this perspective may prove insightful for understanding their molecular functions.

### Overexpression of genes coding for OMPs.

Outer membrane proteins (OMPs) deserve great interest in *Leptospira* and bacteria in general because they are located on the cell surface, where the microorganism interacts with the environment, acting as adhesins, antigens, transporters, or receptors (28). We found several upregulated OMP-encoding genes in mature biofilms.

Probably the most interesting upregulated gene in this context was *LEPBI_Ia0817*, which encodes the outer membrane porin OmpL1, which is a novel leptospiral extracellular matrix (ECM)-binding protein and plasminogen receptor (29). This protein is expressed during infection (27) and presented synergistic immune protection with the lipoprotein LipL41 in *Leptospira kirschneri* infection in hamsters (28). Considering this, the overexpression of *L. biflexa ompL1* suggests that it could also play an important role in the establishment and maintenance of biofilm structure by providing adhesive properties. Another interesting upregulated gene (*LEPBI_I1873*) encodes an OmpA-like protein exclusively present among group III (91% average amino acid identity), with a distant homolog in *Leptonema illini* (43% amino acid identity). The overexpression of OmpA homologs has been identified as important for cell aggregation during biofilm formation in other bacterial species, such as *E. coli* (30) and *Acinetobacter baumannii* (10), suggesting that *LEPBI_I1873* may also be implicated in biofilm aggregation in *L. biflexa* as well.

Five additional genes that code for putative surface-exposed lipoproteins were also identified as upregulated. In particular, *LEPBI_I0009* encodes a surface-exposed lipoprotein confined to group III *Leptospira* (98% average amino acid identity), LipL21 being the closest protein encoded in pathogenic species from group I and II (68% average amino acid identity). LipL21 is an abundant OMP detected *in vivo* during pathogenic *Leptospira* infection (30–32) but absent in the saprophytic *L. biflexa*, suggesting that, in fact, LEPBI_I0009 is a different protein restricted to saprophytes. Another similar case was *LEPBI_I1822*, which encodes a conserved lipoprotein among group III leptospires (92% average amino acid identity), LipL31 being its closest protein in pathogenic species (52% average amino acid identity). Furthermore, the gene *LEPBI_I2674*, which encodes the apolipoprotein *N*-acyltransferase LntB, was upregulated in mature biofilm. This protein is involved in lipoprotein biosynthesis, and its depletion provokes mislocalization of outer membrane lipoproteins (33). The overexpression of *lntB* has been also reported during biofilm formation of *Leptospirillum* (34), constituting additional evidence for the importance of lipoproteins in the development and maintenance of biofilms.

The transcriptional shift of these genes allowed us to hypothesize that molecular mechanisms of biofilm formation can have different actors in saprophytic and pathogenic leptospires. Future work on transcriptomics using pathogenic species during biofilm formation could shed light on these differences. None of the genes discussed in this section were differentially expressed in late biofilm. The main difference observed when comparing mature and late biofilms using dark-field microscopy was that late cultures presented evident signs of detachment, like less-dense biofilm mass, interspersed cellular aggregates with areas devoid of cells, and the presence of planktonic cells. This fact supports the idea that overexpression of OMPs and surface-exposed lipoproteins may be implicated in the structural maintenance of mature biofilms by promoting cell aggregation and adhesion to abiotic or biotic surfaces.

### Metabolism of sugars and lipids.

Sugars and lipids are essential cellular building blocks but also are the main carbon sources for energy production and storage. We found evidence that both sugars and lipids are mainly used to build biofilm matrix components, in particular exopolysaccharides (EPS) and fatty acids.

Galactose is a monosaccharide that can be used as a carbon source via the Leloir pathway, composed of three main enzymes: GalK, GalT, and GalE. The reduced activity of the epimerase GalE (the last step of the pathway) leads to accumulation of UDP-galactose, which is toxic for the cell. Recently, Chai et al. (35) demonstrated for *Bacillus subtilis* that *galE* null mutants presented compensatory mutations in the major biofilm repressor *sinR* that overcome UDP-galactose cytotoxicity. These mutants were characterized by an increased capacity to produce EPS, a major biofilm matrix component. In *L. biflexa*, *galK* (*LEPBI_I0073*) and one *galE*-like gene coding for a UDP-glucose 4-epimerase (*LEPBI_I0113*) were downregulated in mature biofilms (Table 2). This suggests that galactose is not being fully metabolized under that condition and supports the notion of UDP-galactose accumulation. Furthermore, the pioneering characterization of *L. biflexa* lipopolysaccharide (LPS) demonstrated that galactose is abundant in this macromolecule (36), which was further confirmed (5, 37). In addition, it is known that the first step of O-antigen biosynthesis is limited to the incorporation of UDP-NAc-glucosamine or UDP-galactose (38). Despite *sinR* homologs not having been identified in *Leptospira*, our results suggest that galactose metabolism could play a central role in EPS production and biofilm formation using a mechanism analogous to that in *B. subtilis* and that perhaps galactose acts as a modulator of other regulatory genes from *L. biflexa*. Also, two additional genes (*LEPBI_I0037* and *LEPBI_I2021*) related to galactose metabolism were upregulated during mature biofilm. The first one codes for a putative transferase belonging to the AHBA (3-amino-5-hydroxylbenzoic acid) synthase family, which includes galactosyltransferases involved in the glycosylation of several cell structures like LPS. The second is a hypothetical protein-encoding gene, but we found that its product is a putative capsule polysaccharide biosynthesis protein that belongs to a family of membrane exporters. Surprisingly, we found that genes involved in the biosynthesis and transport of other common biofilm matrix polysaccharides like alginate were downregulated in mature biofilm, in particular, the alginate *O*-acetyltransferase AlgI (*LEPBI_II0277*) and a putative alginate export protein encoded by *LEPBI_I3464*. These results indicate that some biofilm components may be produced in the early stages (before 48 h) and that their biosynthesis stops once the mature biofilm has been established, while other components seem to be continuously synthesized. Considering this, the biofilm structure is probably being regulated by differential biosynthesis over time, and stages prior to biofilm maturation need to be evaluated in order to decipher which genes are involved in the onset of biofilm formation.

**TABLE 2:** Description of discussed genes and biological processes

Biological process or category and gene	Symbol	Description	Biofilm, 48 h			Biofilm, 120 h		
			Regulation status	logFC	FDR	Regulation status	logFC	FDR
DNA replication								
* LEPBI_I0001*	*dnaA*	Chromosomal replication initiator protein	Down	−0.3	5e−3			
* LEPBI_I0012*		DNA polymerase III, delta subunit	Down	−0.35	3e−3			
* LEPBI_I3461*	*dnaX1*	DNA polymerase III, gamma subunit	Down	−0.4	9e−5			
* LEPBI_I3479*	*dnaX2*	DNA polymerase III, tau subunit	Down	−0.45	7e−4			
* LEPBI_I3473*	*parB*	Chromosome partitioning protein ParB	Down	−0.3	4e−3			
* LEPBI_II0026*	*parB*	Chromosome partitioning protein ParB	Down	−0.39	1e−3			
* LEPBI_I3477*	*gidA*	Glucose-inhibited partition protein A	Down	−0.38	5e−4			
* LEPBI_I3475*	*gidB*	Glucose-inhibited partition protein B	Down	−0.31	5e−3			
* LEPBI_I0003*	*recF*	DNA replication and repair protein RecF	Down	−0.4	1e−4			
* LEPBI_I0005*	*gyrB1*	DNA gyrase subunit B	Down	−0.3	2e−3			
Cell growth								
* LEPBI_I2249*	*vagC*	Putative virulence- associated protein B	Up	0.9	2e−5			
* LEPBI_I3440*	*hepA*	ATP-dependent RNA helicase	Down	−0.4	1e−5			
* LEPBI_I0011*	*fis*	Fis family transcriptional regulator	Down	−0.45	4e−3	Down	−0.5	3e−4
* LEPBI_I3348*		Pyrroloquinoline quinone	Down	−0.35	3e−4			
Motility								
* LEPBI_I0008*		PilZ domain	Down	−0.31	9e−3			
* LEPBI_II0088*		PilZ domain	Down	−0.5	9e−5	Down	−0.44	1e−3
* LEPBI_I0917*	*cheY1*	Chemotactic response regulator CheY	Up	0.47	6e−5			
* LEPBI_I1764*	*cheR*	Chemotaxis protein methyltransferase	Down	−0.37	1e−3			
* LEPBI_I1589*	*flaB1*	Flagellar filament core protein FlaB	Up	1.19	8e−19			
* LEPBI_I2133*	*flaB2*	Flagellar filament 35-kDa core protein	Up	0.66	4e−9			
* LEPBI_I2132*	*flaB3*	Flagellar filament 35-kDa core protein	Up	0.98	4e−12			
* LEPBI_I2335*	*flaA1*	Flagellar filament outer layer protein A	Up	0.33	6e−3			
Outer membrane proteins								
* LEPBI_Ia0817*	*ompL1*	Outer membrane protein OmpL1	Up	0.88	2e−21	Up	0.31	3e−3
* LEPBI_I1873*		OmpA-like protein	Up	0.38	3e−3			
* LEPBI_I0009*		Putative lipoprotein	Up	0.35	2e−3			
* LEPBI_I1822*		Putative LipL31	Up	0.38	5e−3			
* LEPBI_2674*	*lntB*	Apolipoprotein *N*-acyltransferase LntB	Up	0.64	8e−9			
Sugar metabolism								
* LEPBI_I0073*	*galK*	Galactokinase	Down	−0.48	9e−7			
* LEPBI_I0113*	*galE*	Putative UDP-glucose 4-epimerase	Down	−0.32	9e−4			
* LEPBI_I0037*		Putative transferase	Up	0.37	1e−3			
* LEPBI_I2021*		Putative capsule polysaccharide biosynthesis protein	Up	0.32	8e−3			
* LEPBI_II0277*	*algI*	*O*-Acetyltransferase AlgI	Down	−0.38	5e−4			
* LEPBI_I3464*		Putative alginate export protein	Down	−0.36	1e−3			
Lipid metabolism								
* LEPBI_I0107*		Long-chain fatty acid–CoA ligase	Down	−0.46	9e−6			
* LEPBI_I0104*	*acdA1*	Acyl-CoA dehydrogenase	Down	−0.33	1e−3			
* LEPBI_I0052*		Enoyl-CoA hydratase	Down	−0.36	4e−4			
* LEPBI_I0777*		Putative triglyceride lipase	Up	0.69	1.5e−6			
* LEPBI_II0198*	*fabG*	3-Oxoacyl-ACP reductase	Down	−0.45	7e−7			
* LEPBI_II0199*	*fabG*	3-Oxoacyl-ACP reductase	Down	−0.39	4e−3			
* LEPBI_II0211*	*fabG*	3-Oxoacyl-ACP reductase	Down	−0.38	2e−3			
Iron metabolism								
* LEPBI_I1883*	*fecA*	Iron(III) dicitrate TonB-dependent receptor	Up	0.5	2e−5			
* LEPBI_I2760*	NA^a^	Putative TonB- dependent receptor protein	Up	0.38	1.5e−5			
* LEPBI_I3362*	NA	TonB-dependent receptor protein	Down	−0.32	2.7e−3			
* LEPBI_I0669*	*hemO*	Heme oxygenase HemO	Up	0.5	1.4e−5			
* LEPBI_p0012*	*hemS*	Hemin degradation protein HemS	Up	0.69	5e−4			
* LEPBI_p0015*	*hemT*	ABC-type Fe^3+^-hydroxamate transport system				Up	0.48	3e−4
* LEPBI_p0014*	*hemU*	ABC-type hemin transport system, permease				Up	0.63	8e−7
* LEPBI_p0013*	*hemV*	ABC-type hemin transport system, ATPase				Up	1.1	6e−21
* LEPBI_I2375*	NA	Hemolysin	Up	0.4	1e−3			
Regulators								
* LEPBI_I1327*	*flgM*	Anti-sigma factor FlgM	Up	0.7	1e−7			
* LEPBI_I2676*	*carD*	CarD family transcriptional regulator	Up	0.86	3e−12	Up	0.65	1.9e−7
* LEPBI_I1529*	*pnp*	Polynucleotide phosphorylase/polyadenylase	Up	0.38	7e−3			
* LEPBI_I1944*	*adk*	Adenylate kinase	Up	1.2	4e−9	Up	0.72	5e−4
* LEPBI_I1460*	*fecR*	FecR protein	Up	0.76	6e−11	Up	0.44	2e−4
Uncharacterized genes								
* LEPBI_I0858*	NA	Putative lipase	Up	1.5	8.5e−9			
* LEPBI_I0859*	NA	Putative lipase	Up	1.35	8.5e−30			

a NA, not available.

Regarding lipids, we found that key enzymes involved in fatty acid degradation from hexadecanoate were downregulated in mature biofilms. One of them is a long-chain fatty acid coenzyme A (CoA) ligase (EC 6.2.1.3) encoded by *LEPBI_I0107*, which catalyzes the first step in the breakdown of hexadecanoate into acetyl-CoA*.* Additionally, both *LEPBI_I0104* (*acdA1*) and *LEPBI_I0052* were downregulated. These genes, respectively, code for the acyl-CoA dehydrogenase (EC 1.3.8.7 and EC 1.3.8.8) and the enoyl-CoA hydratase (EC 4.2.1.17), which catalyze downstream enzymatic steps of hexadecanoate degradation. Compositional analyses of the biofilm matrix from many bacteria have revealed the presence of fatty acids (hexadecanoic in particular) in abundance (39–42). Hence, the accumulation of hexadecanoate by downregulation of the enzymes involved in fatty acid degradation may indicate that these lipids could be used for matrix composition in *L. biflexa*. This hypothesis is reinforced by the overexpression of *LEPBI_I0777*, which codes for a triglyceride lipase (EC 3.1.1.3), allocated to glycerolipid metabolism and involved in degrading triglycerides to single fatty acids (Table 2). Furthermore, most enzymes belonging to the canonical pathway of fatty acid biosynthesis from acetyl-CoA were not differentially expressed during biofilm formation, suggesting that fatty acids are being synthesized *de novo* during biofilm. Exceptionally, we found one *fabG* homolog (*LEPBI_II0198*) downregulated. FabG (EC 1.1.1.100) catalyzes a key step of fatty acid biosynthesis, and so its depletion should indicate reduced or null production of fatty acids. Further analysis of the *L. biflexa* genome revealed the presence of 16 putative *fabG* homologs with total conservation of catalytic residues, but just 3 of them (*LEPBI_II0198*, *LEPBI_II0199*, and *LEPBI_II0211*) were downregulated. Beyond their role in biofilm formation, the great level of paralogy for this gene, unique among the genes in the pathway, may suggest functional redundancy or the evolution of substrate-specific FabG isoforms. The fact that only 3 *fabG* copies were downregulated may indicate that the fatty acid biosynthetic pathway is not stopped at the FabG step, but further investigation will be needed for elucidating the striking role of the high redundancy of *fabG* in *L. biflexa* biology.

### Iron uptake.

Iron plays a crucial role in biological processes by composing essential enzyme cofactors or in electron transport chains. *Leptospira* requires an environmental iron source to grow and, like many other bacteria, has evolved diverse strategies to scavenge it from its surroundings. Considering that biofilm-embedded cells are mostly sessile, we thought that these scavenging systems should be transcriptionally altered in response to iron availability. The genomic and functional characterization of iron uptake systems in *L. biflexa* has revealed the presence of redundant outer membrane TonB-dependent receptors (with different specificities for iron-containing compounds), hemolysins, inner membrane hemin transporters, and the FeoAB system but an absence of genes coding for siderophore biosynthesis (43).

A salient feature of mature biofilm was the overexpression of *LEPBI_I1883*, coding for *fecA*. Functional characterization of FecA in *L. biflexa* revealed its capacity to transport diverse iron compounds like aerobactin, iron citrate, iron chloride, and iron sulfate (43). Interestingly, the Ellinghausen, McCullough, Johnson, and Harris (EMJH) medium in which *L. biflexa* was cultured in this work contains iron sulfate as an iron source, suggesting that the sessile condition of biofilm cells requires the upregulation of this TonB-dependent receptor to encompass iron acquisition. Another TonB-dependent receptor-encoding gene (*LEPBI_I2760*) was also overexpressed in mature biofilm, whose disruption impairs the ability to use desferrioxamine as an iron source in *L. biflexa* (43), and a similar TonB-dependent receptor (FoxA) is responsible for desferrioxamine utilization in *Yersinia enterocolitica* (58). Furthermore, an additional TonB-dependent receptor-encoding gene (*LEPBI_I3362*) was downregulated in mature biofilm. Disrupting *LEPBI_I3362* leads to a wild-type phenotype in iron-depleted medium, probably due to functional redundancy with other iron uptake systems (43). Translocation of iron compounds from the periplasmic space to the cytoplasm in *L. biflexa* relies on siderophore- or metal-ABC transporters, the FeoAB system, and the hemin uptake system. Except for the hemin uptake system, none of these transporters were differentially expressed during biofilm formation. We also found that both the gene coding for the heme oxygenase HemO (*LEPBI_I0669*) and that coding for the hemin degradation protein HemS (*LEPBI_p0012*) were upregulated; however, differential expression of the remaining ABC transporter components HemT, HemU, and HemV was detected only in late biofilm. The gene *LEPBI_I2375*, which codes for a hemolysin, was also upregulated. These results show that iron uptake is finely tuned during biofilm formation, considering that only some specific TonB-dependent receptors, the hemin uptake system, and one hemolysin were differentially expressed in this experiment.

### Regulatory genes and coregulation networks.

**FIG 2:**
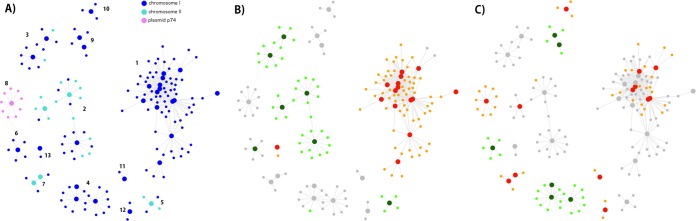
Coexpression networks. The figure shows the 13 coexpression networks that resulted from analyzing positively correlated genes. The big circles represent genes involved in regulatory processes and in panels B and C are colored red if they are upregulated and dark green if they are downregulated. Small circles are colored orange for upregulated genes and light green for downregulated genes. Gray circles are genes without differential expression under that condition. (A) Genes are colored by replicon. (B) Differentially expressed genes at 48 h. (C) Differentially expressed genes at 120 h.

In the sections above, we have presented and discussed the most relevant protein-encoding genes and gene pathways that we found altered when comparing biofilms with planktonic cells in *L. biflexa*, omitting how these genes can be modulated through the action of other regulatory genes. To assess this, we recovered all differentially expressed genes involved in any regulatory step, like transcription factors, and investigated how their transcription levels covaried with the rest of the differentially expressed genes in order to describe coregulation patterns among them. Out of 575 genes differentially expressed under any condition, 47 (8%) were annotated as transcription factors or related proteins involved in regulatory processes. In general, regulators can be classified as activators or repressors if they enhance or reduce the transcription of other genes. In this sense, we found that most regulatory networks were composed of genes whose transcription levels correlated positively, which suggests that most relevant regulatory processes were orchestrated by activators (Fig. 2). Out of the 13 different coactivation networks that were identified, 5 (accounting for 64% of coregulated genes) were differentially expressed in mature biofilm while 8 (accounting for 36% of coregulated genes) were differentially expressed in late biofilm, showing that around two-thirds of coregulatory processes are taking place in mature biofilm.

**FIG 3:**
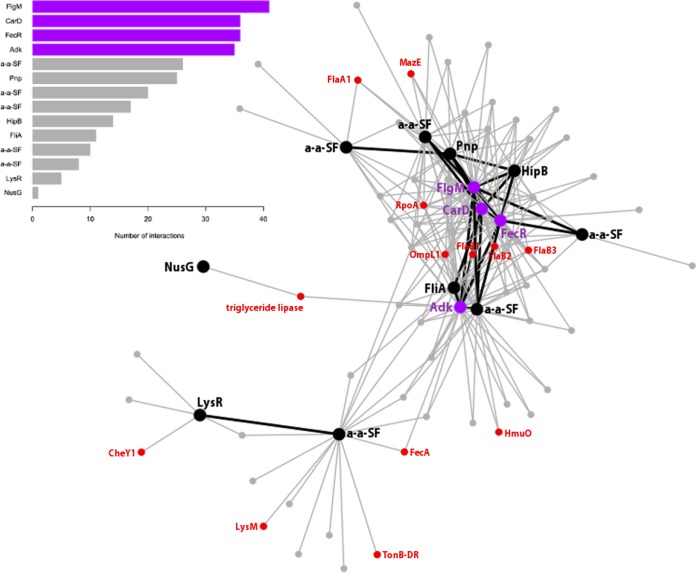
Major coexpression network. The figure shows the biggest coexpression network (network 1 in Fig. 2). The bar graph shows all regulatory genes in the network sorted by the number of interactions (coexpressed genes). The top 4 regulatory genes are highlighted in purple, while the rest are highlighted in black. Other relevant genes that are discussed in the text are in red. Black lines represent coexpression between regulatory genes.

We identified a predominant coregulation network that alone includes 40% of coregulated genes and also contains regulators that direct most relevant functions for biofilm; a detailed description of this network is presented in Fig. 3. The regulator with the highest number of interactions (coexpressed genes) was *LEBI_I1327*, a hypothetical protein-encoding gene. However, more-careful analyses (see Materials and Methods) revealed that it codes for the anti-sigma factor FlgM, which interacts with sigma factor FliA. The presence of FliA and flagellar components FlaA1 and FlaB123 in the network suggested that this system may be responsible for tuning flagellar function and hence bacterial motility. Additionally, the presence of 5 different anti-anti-sigma factors in the network implies that such antagonists also regulate anti-sigma factors like FlgM. This reveals a previously unreported role of the anti-anti-sigma regulatory system in the physiology of *L. biflexa*. However, further experimental work will be needed to confirm our structural annotation and to demonstrate that *LEBI_I1327* codes for FlgM and regulates FliA.

The second regulator in the ranked interactions was *LEPBI_I2676*, encoding a transcription factor resembling mycobacterial CarD, which is overexpressed under hostile conditions like nutrient deprivation (44). Interestingly, the gene coding for the regulatory protein polynucleotide phosphorylase (Pnp; *LEPBI_I1529*) was coexpressed with *LEPBI_I2676*, and a functional relationship between these two genes has been shown in *Mycobacterium tuberculosis* (44). Furthermore, a specific role for Pnp during biofilm formation has been established in *Salmonella*, where the expression of CsgD (the master activator of biofilm formation) was substantially reduced in the *pnp* mutant (59). The same study also identified Pnp as an indirect regulator of cyclic monophosphate nucleotides, which are key second messengers in biofilm formation. In this sense, we found that the *adk* gene (*LEPBI_I1944*), coding for an adenylate/guanylate cyclase, was coexpressed with *pnp.* The underlying molecular mechanism for the regulatory role of Pnp may involve its RNase activity, which selectively degrades mRNAs (46). This incorporates a posttranscriptional regulation step and would make this protein a key modulator of genes involved in *L. biflexa* biofilms.

Another top-scoring coregulator was FecR (*LEPBI_I1460*), which is needed for the induction of the *fecABCDE* iron transport operon in enterobacteria like *E. coli* (47). Interestingly, FecA (*LEPBI_I1883*) is present in this coregulation network, suggesting an important role for this gene pair in iron acquisition. Furthermore, previous studies were unable to identify *fecBCDE* homologs in *L. biflexa*, suggesting a different unknown pathway for these functions (43). The study of the 11 coexpressed genes with FecR, now annotated as hypothetical proteins with remote or no homology in sequence databases, will probably shed light on unknown aspects of iron metabolism in *L. biflexa*.

When analyzing where in CI, CII, or P74 the regulators and their cognate genes were located, we found that for a given regulatory network, almost all genes linked to it were located in the same replicon. However, Fig. 2 also shows that a minority of genes from CI and CII are coexpressed in the same network. This kind of interchromosomal regulation has been found in other bacteria with multiple replicons, like *Vibrio cholerae* (45). At any rate, these findings support the hypothesis introduced previously, namely, that each replicon in *L. biflexa* plays particular roles during biofilm formation, with minimal interaction between gene products from distinct replicons.

### ncRNAs.

Noncoding RNA regulators (ncRNAs) have been identified in a wide range of organisms, including bacteria, and found to play important regulatory roles in several biological processes (48). Recently, a paper describing the transcriptional adaptation of *L. interrogans* to the intrahost environment has shown the expression of ncRNAs in this species (49). The transcription of noncoding regions with ncRNA signatures has not been reported so far in *L. biflexa*. Using *de novo* prediction tools, we identified 181 putative ncRNAs dispersed in the 3 replicons (CI, 168; CII, 11; P74, 2), and just 30 of them (15%) appeared to be transcriptionally active (count per million [CPM] of >1); these active ncRNAs were placed in CI (*n* = 25) and CII (*n* = 5) (see Table S6 in the supplemental material).

Among others, we identified one CsrA-like ncRNA in CI whose activity could modulate the *csrA* homolog of *L. biflexa* (*LEPBI_I3210*), a global translational regulator in many bacterial species which indeed we found upregulated (log fold change [logFC] = 0.66, FDR = 1.5e−5). Additionally, one antisense ncRNA of 93 bp placed in CII next to the alginate biosynthesis genes was downregulated in mature biofilm (logFC = −0.45, FDR = 4e−4). Strikingly, the same ncRNA was upregulated in late biofilm (logFC = 0.58, FDR = 1.4e−4). It is worth mentioning that the alginate *O*-acetyltransferase-encoding gene *algI* was downregulated in mature biofilm and unchanged in late biofilm. Further characterization of this and other candidate ncRNAs is required to understand their role in the regulation of genes involved in biofilm formation.

### Differentially expressed genes coding for proteins of unknown function.

The phylum *Spirochaetes* has evolved many distinctive and often intriguing features since its deep branching in the bacterial phylogeny. Accordingly, a great number of leptospiral genes code for hypothetical proteins with limited or null homology in sequence databases, challenging downstream experimental procedures based on predicted protein functions. In this RNA-seq experiment, we found that 289 out of 575 (50%) genes differentially expressed under any condition were annotated as hypothetical protein-encoding genes. Even after refined manual curation, more than 50 differentially expressed genes remained without any predicted function. Indeed, among the top 5 upregulated genes (ranked by fold change) in mature biofilm we found two consecutive genes (*LEPBI_I0858* and *LEPBI_I0859*) that were originally annotated as hypothetical proteins; however, structural annotation revealed that they probably have a lipase activity. This is a strong evidence that genes encoding hypothetical proteins in *Leptospira* are true and actively transcribed genes whose functions remain to be determined, opening new grounds of research in leptospiral biology. Structural annotations are presented in Table S7 in the supplemental material.

### RT-PCR confirmation of selected differentially expressed genes.

In order to check the robustness and reproducibility of differentially expressed genes detected by the RNA-seq analysis, a set of 21 genes was used to perform relative quantification by reverse transcription real-time PCR (RT-PCR). These genes are representative of the most relevant pathways discussed throughout this work. Figure S2 in the supplemental material shows that for the vast majority of tested genes, the expression levels were consistent with those observed throughout RNA-seq analysis and differences were statistically significant (*P* < 0.05, *t* test). Furthermore, RT-PCRs were performed with a set of template RNAs derived from a biofilm experiment independent of the one used for performing RNA-seq, indicating significant reproducibility of detected transcript switches in these genes. Additionally, we proposed a set of *L. biflexa* genes that can be used for RT-PCR normalization due to their scarce transcription variability along biofilm and planktonic states (see Table S3).

10.1128/mSphere.00042-16.2 Figure S2  RT-PCR relative quantification for selected genes. Asterisks show statistical significance assessed by *t* test (*P* < 0.05). Download Figure S2, PDF file, 0.2 MB. Copyright © 2016 Iraola et al. 2016 Iraola et al. This content is distributed under the terms of the Creative Commons Attribution 4.0 International license.

### Integrative view of gene expression during biofilm formation.

In this work, we describe the first RNA-seq experiment performed over the model organism *L. biflexa* oriented to gene expression changes in biofilms, over abiotic surfaces at two time points (48 h and 120 h). At 48 h, *L. biflexa* reaches an optimal biofilm growth designated mature (11), which, compared to the planktonic state at the same time, allowed us to identify the chief genetic factors differentiating biofilm from free-living states. At 120 h, the (late) biofilm structure presents signs of cell disaggregation, showing the known detachment process responsible for biofilm-to-planktonic cell recycling or even cell death. In this sense, some genetic changes associated with this process could be identified, such as expression levels of several genes involved in adhesion and EPS production. However, and despite our paper describing a clear transcription turnover between biofilm and planktonic states, performing transcriptomics on biofilm cultures before 48 h will contribute to identifying additional features that determine biofilm formation and, in particular, those genes that govern the initial phase of interaction between cells and the abiotic surface.

As an outline, our results highlighted many functions related to cell growth and metabolism that were altered during biofilm formation, DNA replication and cell division probably being the most notable. Additionally, other well-recognized pathways like sugar, lipid, and iron metabolism presented transcriptional regulation. Beyond identifying the role of these well-known metabolic pathways during biofilm formation, we made annotation improvements for many genes lacking assigned molecular functions. In this sense, a most remarkable case is *LEBI_I1327*, which was previously annotated as a hypothetical protein but which we propose to be homologous to *flgM*. Indeed, it seems to be the most relevant regulatory gene during biofilm formation based on our coexpression analysis. Furthermore, we reported for the first time in *L. biflexa* the presence of small RNA (sRNA) regulators that were transcriptionally active during biofilm and planktonic growth. Despite identification of some candidate sRNAs for the regulation of particular processes like alginate biosynthesis, a more detailed and specific work centered on the analysis of sRNAs is required to understand their targets and regulation networks.

Another hint shown by our results is a possible differentiation between biofilm formation mechanisms in saprophytic and pathogenic leptospires, as attested by the presence of differentially expressed genes probably involved in cell-to-cell adhesion that were not found in the genomes of pathogenic *Leptospira*. In particular, as the pathogenic *L. interrogans* shares with *L. biflexa* the free-living trait, in which biofilm formation probably is crucial for bacterial persistence, elucidating transcriptional changes in *L. interrogans* during biofilm formation would be very informative for determining if both species have evolved particular features associated with this growth condition. Finally, taking into account the recent availability of genomic sequences for several leptospiral species with differential incidences in leptospirosis, we consider that further extending RNA-seq analyses to species belonging to groups I, II, and III could shed more light on the evolution of this striking organism, as well as contributing to generation of effective tools for leptospirosis control.

## MATERIALS AND METHODS

### *Leptospira biflexa* cultures and biofilm experiments.

*Leptospira biflexa* serovar Patoc strain Patoc I (Institut Pasteur, Paris, France) was a gift from Centro de Pesquisas Gonçalo Moniz (CPqGM), Fundação Oswaldo Cruz (Fiocruz), Bahia, Brazil. Bacteria were cultured in Ellinghausen, McCullough, Johnson, and Harris (EMJH) liquid medium (Difco, USA) at 29°C, without shaking. *L. biflexa* was replicated without shaking 10 times in liquid EMJH medium before performing biofilm experiments.

Biofilms were grown in borosilicate glass tubes (16 mm by 100 mm) containing 5 ml liquid EMJH medium. A starting culture in mid-exponential growth phase (~10^7^ leptospires/ml, after 48-h incubation) was expanded to 30 tubes, each containing 5 ml liquid EMJH medium (1:10, vol/vol), making six biological replicates of five tubes each. Biofilms were harvested at two time points: (i) after 48 h of incubation, when biofilms are considered to be in a mature stage, and a dense halo is visible attached to the wall of glass tubes at the air-liquid interface (here referred to as mature biofilm), and (ii) after 120 h of incubation, in a late culture stage, when biofilms are detaching (here referred to as late biofilm). Biofilms were visually inspected using dark-field microscopy by removing the biofilm mass from the tube wall in order to check for cell motility, aggregation/detachment, and biofilm mass integrity. At 48 h and 120 h, three biological replicates were randomly chosen. Liquid EMJH medium was discarded, and the biofilms were rinsed with 6 ml cold liquid EMJH medium to remove unattached bacteria. To each glass tube, 400 µl RNAprotect reagent (Qiagen, USA) was added, and biofilms were scraped using stainless steel sterile spatulas. The unavoidable destruction of the biofilm heterogeneity during sample preparation prevents the study of gene expression patterns across different populations within the biofilm; hence, the results obtained will reflect an average expression pattern of the whole biofilm. Planktonic cells were cultured in polypropylene tubes. A starting culture with 48-h incubation (~10^7^ leptospires/ml) was replicated to six polypropylene tubes containing 10 ml liquid EMJH medium each (1:10, vol/vol). At 48 h and 120 h, three tubes, representing three biological replicates, were randomly selected. From each tube, 1 ml of planktonic culture was transferred to another plastic tube containing 2 ml of RNAprotect reagent (Qiagen, USA).

### RNA purification and sequencing.

Total RNA for each biological condition and replicate was isolated using the RNeasy Protect bacterial minikit (Qiagen, USA), according to the manufacturer’s protocol. For the planktonic condition, 1 ml of liquid culture medium was used as starting material. For the biofilm condition, the biofilm mass was mechanically removed from the glass tube and homogenized in 1 ml of phosphate-buffered saline (PBS). The Ribo-Zero magnetic kit (bacteria) (Epicentre, USA) was used to deplete rRNA from 1 µg of total RNA. Obtained rRNA-depleted RNA was quantified with the Qubit RNA HS assay kit (Invitrogen, USA). The ScriptSeq v2 RNA-seq library preparation kit (Epicentre, USA) was used from 50 ng of rRNA-depleted RNA. Index primers were added to each library to allow sequence multiplexing. After 12 PCR cycles, the final library was purified with AMPure XP (Benchman, USA) and quantified with the Qubit dsDNA HS assay kit (Invitrogen, USA). Quality and length of the library were assessed with the Agilent high-sensitivity DNA kit (Agilent, USA) using the 2100 Bioanalyzer (Agilent, USA). Sequencing was performed on an Illumina Genome Analyzer II X platform at the Institut Pasteur, Montevideo, Uruguay, and generated 45,365,550 single-end reads (72 cycles).

### Detection of differentially expressed genes.

All statistical analyses were implemented in R (50). Read alignment and counting were performed using the Rsubread package (51). Read duplicates were kept as for most samples; at the coverage reached, more than one “real” duplicate is expected at each starting position. The minimum, mean, and maximum read numbers per sample were 2,631,490, 3,780,463, and 7,500,998, respectively; the proportion of mapped reads was greater than 98.6% for all samples. Differential expression analysis was carried out with the edgeR package (52). After previous analysis and visual inspection, 3 samples (BC48, PA48, and PC120) were discarded because they showed discordant expression patterns when considering time and source, probably due to problems inherent to cell manipulation. Genes with less than one count per million (CPM of <1) in any of the samples were also discarded (15 genes). A simple factorial model with two factors, time (48 and 120 h) and mode (planktonic and biofilm), was fitted; a false discovery rate (FDR) of <1e−2 was considered the threshold for differentially expressed genes.

Noncoding RNA genes (ncRNAs) were predicted with RNAspace (53), which combines the results of several *de novo* prediction tools for RNAs. Parameters were set as default, and all predicted RNAs were kept at first. Genes with a CPM of >1 or with at least 2 reads in at least 2 samples were defined as transcriptionally active. CsrA-like ncRNAs were identified by implementing the algorithm described in the work of Kulkarni et al. (54). The identification of differentially expressed ncRNAs was performed as described in the paragraph above.

### Functional annotation and coexpression analyses.

When interesting genes were annotated as hypothetical proteins in the current version of the *L. biflexa* strain Patoc I genome, additional efforts were made to predict molecular functions. In the first place, runs of BLASTP and CDD-search against the nr database (NCBI) were used to identify annotated bacterial homologs. Additionally, over the remaining set of proteins with unknown function, structural annotations were attempted using the HH suite package and database (55). Briefly, for each query protein, a profile hidden Markov model (HMM) was built using HHblits (56), with three rounds of searches over a nonredundant HMM database. Next, the resulting HMM was used as a query over the HMM database for the Protein Data Bank culled at 70% sequence identity (PDB70) provided by the authors, using the HHsearch program (55). Results were manually inspected, and when possible, a structural/functional feature was assigned to the query protein.

A simple analysis of coexpression networks was performed considering the correlation matrices of gene expression (CPM) across samples. For selected genes, a correlation coefficient value greater than 0.96 was arbitrarily set as a threshold for gene clustering. Upon visual inspection and analysis of cluster contents, only positive correlations were graphically represented using the igraph R package (57).

### Confirmation of differentially expressed genes by RT-PCR.

Twenty-one genes were selected for testing their expression levels using a real-time PCR protocol for relative transcript quantification. This experiment was performed using RNA purified from an independent experiment, different than the one used for RNA-seq to check the robustness and reproducibility of the results. For all samples, 100 ng of total RNA was used to synthesize first-strand cDNA with reverse transcriptase SuperScript II (Invitrogen, USA) and oligo(dT). The cDNA synthesis was performed at 42°C for 50 min after heat inactivation at 70°C for 10 min. The primer sequences designed for selected genes are listed in Table S2 in the supplemental material. PCR was performed using 1× KAPA SYBR Fast qPCR kit master mix (Kappa, USA) on an Illumina Eco machine (Illumina, USA). For all genes, cycling conditions were as follows: 2 min at 95°C and 40 cycles of 10 s at 95°C, plus 30 s at 60°C. The Eco study software (Illumina, USA) was used to calculate threshold cycle (ΔΔ*C_T_*) relative expression values for all the genes studied. For endogenous normalization of expression levels, we selected a set of 6 genes (see Table S3) that showed the lowest count variation among samples in the RNA-seq experiment. As differences in the performance of all genes as normalizers were not significant, we selected one of them (*LEPBI_I2771*) for presenting RT-PCR results.

10.1128/mSphere.00042-16.3 Table S1  Information for data deposited at Sequence Read Archive. Download Table S1, PDF file, 0.03 MB. Copyright © 2016 Iraola et al. 2016 Iraola et al. This content is distributed under the terms of the Creative Commons Attribution 4.0 International license.

10.1128/mSphere.00042-16.4 Table S2  Primers used in RT-PCR analysis. Download Table S2, PDF file, 0.04 MB. Copyright © 2016 Iraola et al. 2016 Iraola et al. This content is distributed under the terms of the Creative Commons Attribution 4.0 International license.

10.1128/mSphere.00042-16.5 Table S3  Genes used for normalization in RT-PCR relative quantification. Download Table S3, PDF file, 0.04 MB. Copyright © 2016 Iraola et al. 2016 Iraola et al. This content is distributed under the terms of the Creative Commons Attribution 4.0 International license.

10.1128/mSphere.00042-16.6 Table S4  Reads mapped by sample. Download Table S4, PDF file, 0.04 MB. Copyright © 2016 Iraola et al. 2016 Iraola et al. This content is distributed under the terms of the Creative Commons Attribution 4.0 International license.

10.1128/mSphere.00042-16.7 Table S5  Differentially expressed genes in all comparisons. Download Table S5, PDF file, 0.2 MB. Copyright © 2016 Iraola et al. 2016 Iraola et al. This content is distributed under the terms of the Creative Commons Attribution 4.0 International license.

10.1128/mSphere.00042-16.8 Table S6  Expression of predicted sRNAs. Download Table S6, PDF file, 0.05 MB. Copyright © 2016 Iraola et al. 2016 Iraola et al. This content is distributed under the terms of the Creative Commons Attribution 4.0 International license.

10.1128/mSphere.00042-16.9 Table S7  Annotation based on manual and structural curation. Download Table S7, PDF file, 0.1 MB. Copyright © 2016 Iraola et al. 2016 Iraola et al. This content is distributed under the terms of the Creative Commons Attribution 4.0 International license.

### Sequencing data accession numbers.

Data were deposited in the Sequence Read Archive (SRA) database under accession numbers SAMN04364752 through SAMN04364763 (BioProject accession number PRJNA288909).
