# Supplementary figures and images for: Transcriptome Sequencing Reveals Wide Expression Reprogramming of Basal and Unknown Genes in Leptospira biflexa Biofilms
Source: mSphere. 2016 Apr 6;1(2):e00042-16. doi: 10.1128/mSphere.00042-16 (PMC4863578; doi:10.1128/mSphere.00042-16)

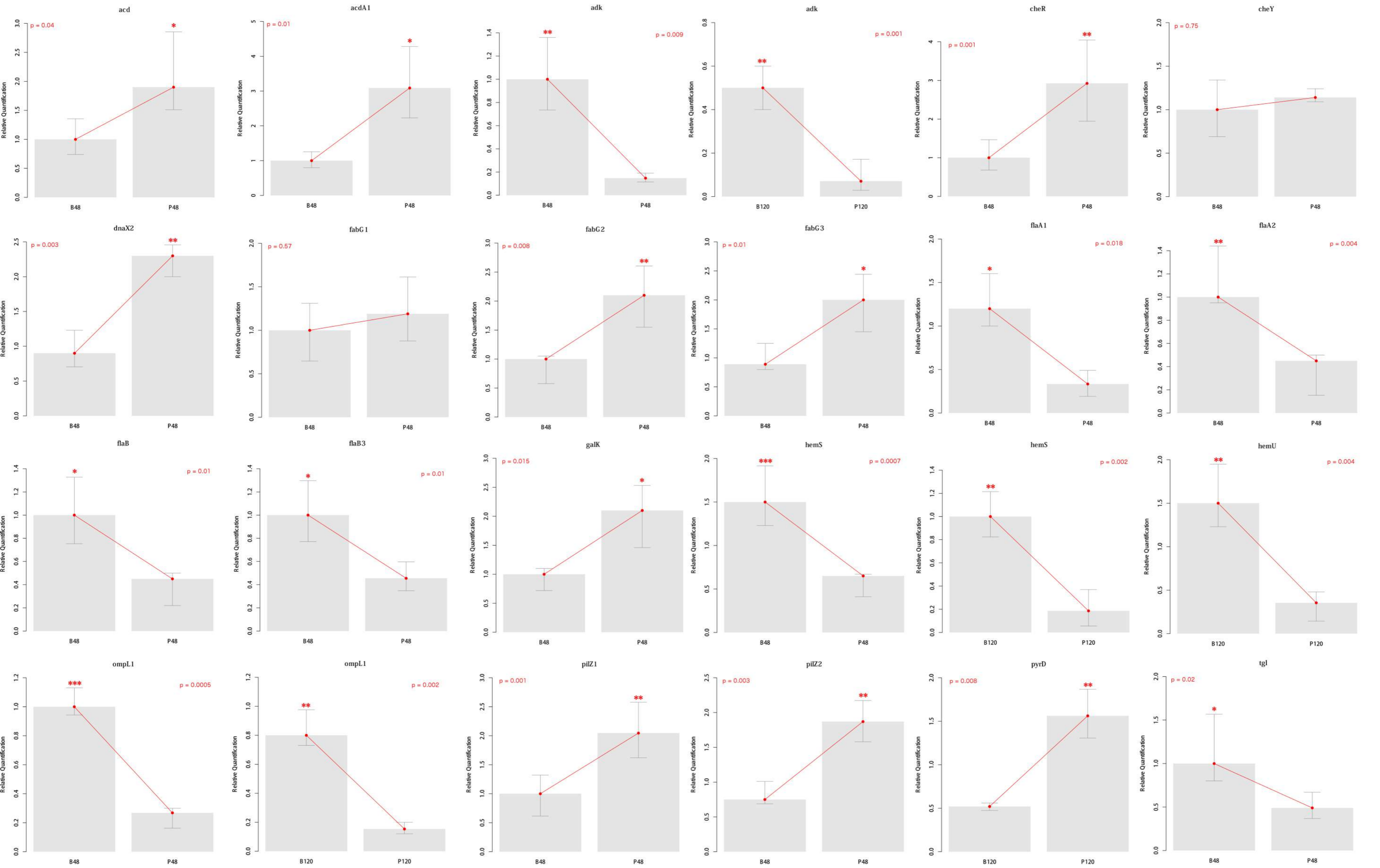

Supplement: Figure S2 [file sph002162059sf2.pdf]
