# Supplementary material for: Transcriptome Sequencing Reveals Wide Expression Reprogramming of Basal and Unknown Genes in Leptospira biflexa Biofilms
Source: mSphere. 2016 Apr 6;1(2):e00042-16. doi: 10.1128/mSphere.00042-16 (PMC4863578; doi:10.1128/mSphere.00042-16)
Supplement: Table S1 [file sph002162059st3.pdf]

Table S1. Information for deposited data at Sequence Read Archive.

BioProject	Accession	Sample Name	Organism	Strain	TaxID	SRA object
PRJNA288909	SAMN04364752	BA48	Leptospira biflexa serovar Patoc	Patoc I (Paris)	172	www.ncbi.nlm.nih.gov/sra/4364752
PRJNA288909	SAMN04364753	BB48	Leptospira biflexa serovar Patoc	Patoc I (Paris)	172	www.ncbi.nlm.nih.gov/sra/4364753
PRJNA288909	SAMN04364754	BC48	Leptospira biflexa serovar Patoc	Patoc I (Paris)	172	www.ncbi.nlm.nih.gov/sra/4364754
PRJNA288909	SAMN04364755	PA48	Leptospira biflexa serovar Patoc	Patoc I (Paris)	172	www.ncbi.nlm.nih.gov/sra/4364755
PRJNA288909	SAMN04364756	PB48	Leptospira biflexa serovar Patoc	Patoc I (Paris)	172	www.ncbi.nlm.nih.gov/sra/4364756
PRJNA288909	SAMN04364757	PC48	Leptospira biflexa serovar Patoc	Patoc I (Paris)	172	www.ncbi.nlm.nih.gov/sra/4364757
PRJNA288909	SAMN04364758	BA120	Leptospira biflexa serovar Patoc	Patoc I (Paris)	172	www.ncbi.nlm.nih.gov/sra/4364758
PRJNA288909	SAMN04364759	BB120	Leptospira biflexa serovar Patoc	Patoc I (Paris)	172	www.ncbi.nlm.nih.gov/sra/4364759
PRJNA288909	SAMN04364760	BC120	Leptospira biflexa serovar Patoc	Patoc I (Paris)	172	www.ncbi.nlm.nih.gov/sra/4364760
PRJNA288909	SAMN04364761	PA120	Leptospira biflexa serovar Patoc	Patoc I (Paris)	172	www.ncbi.nlm.nih.gov/sra/4364761
PRJNA288909	SAMN04364762	PB120	Leptospira biflexa serovar Patoc	Patoc I (Paris)	172	www.ncbi.nlm.nih.gov/sra/4364762
PRJNA288909	SAMN04364763	PC120	Leptospira biflexa serovar Patoc	Patoc I (Paris)	172	www.ncbi.nlm.nih.gov/sra/4364763
