# Supplementary material for: Transcriptome Sequencing Reveals Wide Expression Reprogramming of Basal and Unknown Genes in Leptospira biflexa Biofilms
Source: mSphere. 2016 Apr 6;1(2):e00042-16. doi: 10.1128/mSphere.00042-16 (PMC4863578; doi:10.1128/mSphere.00042-16)
Supplement: Table S2 [file sph002162059st4.pdf]

Table S2. Primers used in RT-PCR analysis.

Gene	Name	Sequence (5' -> 3')	Strand	Bp	Tm	GC
LEPBI_I0073	galK-F	AGTGGTAACTGGCTTTGCGA	Plus	20	59.89	50
	galK-R	GCTTTCTGTCCAATCACGGC	Minus	20	59.83	55
LEPBI_I3479	dnaX2-F	AGAACCGCCTCCACATACAA	Plus	20	59.02	50
	dnaX2-R	CTGCGGGAGGTAGTGGAAAG	Minus	20	60.11	60
LEPBI_I1589	flaB-F	GTCTAACGACGCGAACCTGA	Plus	20	60.11	55
	flaB-R	CTGCAAGTCCAGATGCGTCA	Minus	20	60.67	55
LEPBI_I1944	adk-F	CAAGCAAAGGCTCTCTCGGA	Plus	20	59.75	50
	adk-R	AGCGTCCTTCTTTGATCGCT	Minus	20	59.61	55
LEPBI_Ia0817	ompL1-F	AGTGGGTTCGGTCTCAACTG	Plus	20	59.61	55
	ompL1-R	GAGCAGAAGCTCCACCGATT	Minus	20	60.11	55
LEPBI_I2132	flaB3-F	GCAAACGCAAGGCAAAGAGA	Plus	20	59.97	50
	flaB3-R	TTTCCAGGTGTCGAGAGTGC	Minus	20	59.97	55
LEPBI_p0012	hemS-F	AATTCGAGACGCAGCCAAAC	Plus	20	59.48	50
	hemS-R	CCCAAGTTTTGGCGTTTCCA	Minus	20	59.54	50
LEPBI_p0014	hemU-F	AGTTTGGGAGGGGCATCTTG	Plus	20	59.96	55
	hemU-R	CCAAGTGACCTGCTTCTCGT	Minus	20	59.97	55
LEPBI_I0092	pyrD-F	GACTTGCCGCTGGATTTGAC	Plus	20	59.83	50
	pyrD-R	TTTGGCTGTGATGGTTCCGA	Minus	20	59.89	55
LEPBI_I0008	pilZ1-F	GACTAGCCTTTCAAACGACA	Plus	20	55.72	45
	pilZ1-R	AGAGGTTTGAAAATCACCGA	Minus	20	54.89	40
LEPBI_II0088	pilZ2-F	GTATCCAAAGGCAAAAGTGG	Plus	20	54.84	45
	pilZ2-R	TATCGTCCTCAAAAAGTTGGT	Minus	21	55.16	38
LEPBI_I0917	cheY1-F	GGTATGACGGGAATCGAATTA	Plus	21	55.29	43
	cheY1-R	GGTTTTACAAGCCAACCAAC	Minus	20	55.66	45
LEPBI_I1764	cheR-F	TTACTCCAGTTTCCGTTTCC	Plus	20	55.31	45
	cheR-R	AGGATCAAATACCCTTTGGG	Minus	20	54.65	45
LEPBI_I2335	flaA1-F	TGAATCTTGGGACAATCCAG	Plus	20	55.02	45
	flaA1-R	GATTTTGCTGGGTCATTCAG	Minus	20	54.93	45
LEPBI_I2336	flaA2-F	ACAGACACACCTTATTTGCT	Plus	20	54.88	40
	flaA2-R	TTGCTGTCAACTTTCTCCAT	Minus	20	55.17	40
LEPBI_I0104	acdA1-F	AAGAGTATGGTGGTATGGGT	Plus	20	55.19	45
	acdA1-R	TCCTTGTTGTGCTTGGATTA	Minus	20	54.86	40
LEPBI_I0052	acd-F	ATGAGAGACCTTGGTGAGAT	Plus	20	55.23	45
	acd-R	TTTCTGCATCCAATCCGTTA	Minus	20	55.05	40
LEPBI_I0777	tgl-F	TTTTTAGCGACCCTTCTCTC	Plus	20	55.1	45
	tgl-R	CCTCCCCAATACTTTACGAG	Minus	20	54.95	50
LEPBI_II0198	fabG1-F	AAGGATTCGATTGTTCTCGT	Plus	20	54.8	40
	fabG1-R	GGTTTCCTGTAGAATGGGTT	Minus	20	54.92	45
LEPBI_II0199	fabG2-F	CGAACTATCTCTTGCTGGAA	Plus	20	54.31	45
	fabG2-R	TACACAATGAGTTCTGGACG	Minus	20	55.14	45
LEPBI_II0211	fabG3-F	CGAAGAACTTGCCATTTACC	Plus	20	54.99	45
	fabG3-R	ACACGATGGAGGATACAATC	Minus	20	54.65	45
LEPBI_I2771	LEPBI_I2771-F	CTCTCGGTGGAGTTTTCGGT	Plus	20	59.68	55
	LEPBI_I2771-R	AACAAATCCCTTCGCCAGCA	Minus	20	60.64	50
