# Supplementary material for: Transcriptome Sequencing Reveals Wide Expression Reprogramming of Basal and Unknown Genes in Leptospira biflexa Biofilms
Source: mSphere. 2016 Apr 6;1(2):e00042-16. doi: 10.1128/mSphere.00042-16 (PMC4863578; doi:10.1128/mSphere.00042-16)
Supplement: Table S3 [file sph002162059st5.pdf]

Table S3. Genes used for normalization in RT-PCR relative quantification.

Gene	Position	Strand	PID	Product	CPM								
					BA120	BA48	BB120	BB48	BC120	PA120	PB120	PB48	PC48
LEPBI_I1415	1475003..1476904	Minus	183220804	hypothetical protein	439.3144	458.2669	446.4948	433.2076	453.9055	467.2638	433.2911	442.8527	424.992
LEPBI_I1808	1889568..1891535	Plus	183221194	hypothetical protein	472.9315	476.9131	473.6058	486.5844	462.4646	492.072	459.8609	465.6223	480.1459
LEPBI_I2349	2419330..2420382	Minus	183221720	Mrp family ATP-binding protein	372.4622	386.1203	381.2275	373.9328	380.1873	359.5911	389.1445	371.2439	379.0303
LEPBI_I2735	2827100..2828275	Minus	183222093	phpshoribosylglycinamide formyltransferase 2	332.7329	344.6683	338.0508	327.0437	360.0321	339.898	360.1222	351.7742	334.6008
LEPBI_I2771	2869795..2871150	Plus	183222128	nitrite extrusion protein 1 NarK	503.8746	479.0646	477.9569	478.6221	495.3203	485.9339	490.5183	474.5322	471.8729
LEPBI_I3250	3364591..3366072	Minus	183222595	hypothetical protein	343.0473	357.7207	343.0713	327.0437	354.7863	342.4555	350.3118	350.7842	337.3584
