# Supplementary material for: Transcriptome Sequencing Reveals Wide Expression Reprogramming of Basal and Unknown Genes in Leptospira biflexa Biofilms
Source: mSphere. 2016 Apr 6;1(2):e00042-16. doi: 10.1128/mSphere.00042-16 (PMC4863578; doi:10.1128/mSphere.00042-16)
Supplement: Table S4 [file sph002162059st6.pdf]

Table S4. Reads mapped by sample.

Sample	Total reads	Mapped reads	Percentage mapped
BA120	2804203	2782554	99,23
BA48	7500998	7411156	98,80
BB120	3207624	3174615	98,97
BB48	3642931	3603771	98,93
BC120	3875081	3847960	99,30
BC48	3803957	3770114	99,11
PA120	4203432	4150680	98,75
PA48	2988218	2947702	98,64
PB120	2631490	2598663	98,75
PB48	3248509	3217719	99,05
PC120	3963865	3921581	98,93
PC48	3495242	3465387	99,15
