# Supplementary material for: Transcriptome Sequencing Reveals Wide Expression Reprogramming of Basal and Unknown Genes in Leptospira biflexa Biofilms
Source: mSphere. 2016 Apr 6;1(2):e00042-16. doi: 10.1128/mSphere.00042-16 (PMC4863578; doi:10.1128/mSphere.00042-16)
Supplement: Table S5 [file sph002162059st7.pdf]

Table S5. Differentially expressed genes in BvsP_48.

Gene	Synonym	logFC	logCPM	FDR	Symbol	Product
ABZ96148	LEPBI_I0001	-0.2774406778	8.5116201028	0.0053046021	dnaA	chromosomal replication initiator protein DnaA
ABZ96150	LEPBI_I0003	-0.4022559485	8.4323463739	0.000139877	recF	DNA replication and repair protein RecF
ABZ96152	LEPBI_I0005	-0.3139795549	9.0950452965	0.0018130464	gyrB1	DNA gyrase subunit B
ABZ96155	LEPBI_I0008	-0.3074474689	7.2818832392	0.0096018139	NA	hypothetical protein
ABZ96156	LEPBI_I0009	0.3502868581	7.5171605832	0.0028384439	NA	putative lipoprotein
ABZ96158	LEPBI_I0011	-0.4534660497	6.9621288105	0.0042304467	NA	putative transcriptional regulator
ABZ96159	LEPBI_I0012	-0.357484434	8.8520236062	0.0031133869	NA	hypothetical protein
ABZ96166	LEPBI_I0019	-0.3098364837	8.9794109813	0.0084875894	NA	hypothetical protein
ABZ96171	LEPBI_I0024	-0.3863406079	9.3867439699	0.0027528366	NA	beta-galactosidase
ABZ96173	LEPBI_I0026	-0.4777871545	7.4091532672	0.0004077755	NA	putative inositol monophosphatase
ABZ96175	LEPBI_I0028	-0.442003301	9.0412007891	0.000146355	trpE	anthranilate synthase component I
ABZ96177	LEPBI_I0030	-0.3347394772	8.1861618677	0.0070359004	NA	hypothetical protein
ABZ96182	LEPBI_I0035	-0.477146564	9.0393467659	4.64326737848941e-06	NA	putative signal peptide
ABZ96184	LEPBI_I0037	-0.373757529	9.1252962907	0.0012352623	NA	putative transferase
ABZ96186	LEPBI_I0039	-0.3736306437	8.1434524694	0.0003596931	NA	hypothetical protein
ABZ96190	LEPBI_I0043	-0.4706324501	8.4109368398	0.0077450449	NA	aromatic-ring-opening dioxygenase domain-containing protein
ABZ96191	LEPBI_I0044	-0.4604881242	7.4691987591	5.91834924947306e-05	NA	putative carboxylesterase 2
ABZ96192	LEPBI_I0045	-0.3698842168	9.0717845682	0.0013549259	NA	hypothetical protein
ABZ96196	LEPBI_I0049	-0.4669239353	8.9020987668	2.40313634841349e-05	purB	adenylosuccinate lyase
ABZ96198	LEPBI_I0051	-0.3512405894	8.8630789741	0.0030706015	NA	hypothetical protein
ABZ96199	LEPBI_I0052	-0.3622163973	8.1845996565	0.0004859711	NA	enoyl-CoA hydratase
ABZ96208	LEPBI_I0061	-0.3769135626	10.1014463632	0.0087672092	NA	hypothetical protein
ABZ96213	LEPBI_I0066	-0.3917368362	7.486913097	0.0033413964	NA	hypothetical protein
ABZ96217	LEPBI_I0070	-0.3825863243	9.449611773	0.004681717	NA	hypothetical protein
ABZ96220	LEPBI_I0073	-0.4819391668	8.5328401613	9.1654086500572e-07	galK	galactokinase
ABZ96224	LEPBI_I0077	-0.3314721003	9.0017014354	0.0021136793	NA	putative sensor protein
ABZ96235	LEPBI_I0088	-0.298835188	8.9357120588	0.003460699	NA	putative signal peptide
ABZ96237	LEPBI_I0090	-0.4157240886	9.2897223871	0.001569541	NA	putative oxidase
ABZ96238	LEPBI_I0091	-0.3030960644	8.1027805627	0.0076325689	NA	putative tRNA (uracil-5-)-methyltransferase
ABZ96244	LEPBI_I0097	-0.4700054339	8.0563553901	7.79334221119504e-07	NA	ankyrin repeat-containing protein
ABZ96245	LEPBI_I0098	-0.3970132974	9.0448348634	3.63414633235725e-05	NA	hypothetical protein
ABZ96246	LEPBI_I0099	-0.4288183405	8.6933414056	7.04021669116444e-06	NA	hypothetical protein
ABZ96248	LEPBI_I0101	-0.518142507	8.0990336105	6.99181096563781e-06	NA	hypothetical protein
ABZ96249	LEPBI_I0102	-0.4350157667	8.8243622577	2.16054772528284e-06	NA	putative oxidoreductase
ABZ96251	LEPBI_I0104	-0.3341687746	8.8338910337	0.0013774153	acdA1	acyl-CoA dehydrogenase
ABZ96252	LEPBI_I0105	-0.3557708678	7.7921401064	0.0009485027	NA	hypothetical protein
ABZ96253	LEPBI_I0106	-0.4518437306	7.6132521374	0.0003824709	NA	putative short chain dehydrogenase
ABZ96254	LEPBI_I0107	-0.4636723048	9.4566216282	9.3523605875722e-06	NA	long-chain-fatty-acid--CoA ligase
ABZ96256	LEPBI_I0109	-0.3706246578	8.8295025643	0.0001259528	NA	putative methyl-accepting chemotaxis protein
ABZ96257	LEPBI_I0110	-0.4424158665	8.2890135629	0.0006287292	NA	hypothetical protein
ABZ96258	LEPBI_I0111	-0.3277726468	8.7237083684	0.0007867803	NA	putative signal peptide
ABZ96259	LEPBI_I0112	-0.3279609349	8.8962597971	0.0011635074	NA	hypothetical protein
ABZ96260	LEPBI_I0113	-0.3239615854	8.0897790689	0.0009579837	NA	putative UDP-glucose 4-epimerase
ABZ96264	LEPBI_I0117	-0.3095108304	7.4960970408	0.009654403	NA	hypothetical protein
ABZ96274	LEPBI_I0127	-0.370392573	7.9396127742	0.0082841466	NA	putative HTH-type transcriptional regulator
ABZ96338	LEPBI_I0193	-0.3291644987	8.5455384924	0.0045109581	NA	hypothetical protein
ABZ96345	LEPBI_I0200	-0.3289226024	9.3020386085	0.0025947351	NA	putative sodium/hydrogen exchanger
ABZ96371	LEPBI_I0226	-0.5570336031	6.5980863012	4.26109554257761e-05	NA	hypothetical protein
ABZ96383	LEPBI_I0238	0.8723966739	8.4718942078	0.0017471752	ispE	4-diphosphocytidyl-2-C-methyl-D-erythritol kinase
ABZ96421	LEPBI_I0277	-0.6563696817	7.6638244092	7.90027845084163e-09	NA	hypothetical protein

ABZ96433	LEPBI_I0289	1.8438733273	7.5568783639	1.06914352459008e-15	NA	hypothetical protein
ABZ96457	LEPBI_I0313	0.448373108	8.6493150698	0.0003083947	NA	putative signal peptide
ABZ96563	LEPBI_I0421	0.7195404874	6.3083556045	4.43574846217621e-08	NA	hypothetical protein
ABZ96565	LEPBI_I0423	0.3211201899	7.3499368732	0.0076325689	NA	putative signal peptide
ABZ96582	LEPBI_I0443	0.8056206823	4.9832349658	0.000957185	NA	hypothetical protein
ABZ96659	LEPBI_I0521	0.6180908237	6.1395450172	0.0002147941	NA	anti-sigma factor antagonist
ABZ96725	LEPBI_I0587	0.3549662311	7.327459629	0.0017342727	NA	hypothetical protein
ABZ96800	LEPBI_I0667	0.9032099913	6.0998438356	5.78093429758115e-07	NA	putative signal peptide
ABZ96802	LEPBI_I0669	0.5017394719	7.5789632707	1.43041796884428e-05	hmuO	heme oxygenase
ABZ96808	LEPBI_I0676	0.4991770368	6.5858554519	0.004016948	NA	hypothetical protein
ABZ96814	LEPBI_I0682	0.3674286703	6.1965768182	0.0078757222	NA	hypothetical protein
ABZ96815	LEPBI_I0683	0.4540808952	7.9980262479	9.5023337179605e-05	NA	putative lipoprotein
ABZ96907	LEPBI_I0777	0.6868192459	7.6419021	1.54041528097758e-06	NA	putative triglyceride lipase
ABZ96925	LEPBI_I0796	0.3531508652	7.5885669957	0.0021221783	gpo	glutathione peroxidase
ABZ96937	LEPBI_I0809	0.6973518276	6.1705809417	0.0002150987	NA	hypothetical protein
ABZ96945	LEPBI_Ia0817	0.8786248977	8.9245698738	2.09177309471283e-21	ompL1	outer membrane protein
ABZ96985	LEPBI_I0858	1.4839124032	8.5411101175	2.01753335334787e-09	NA	hypothetical protein
ABZ96986	LEPBI_I0859	1.3491070558	8.5895762377	7.03663828092268e-30	NA	hypothetical protein
ABZ96987	LEPBI_I0860	0.7552571541	8.267598476	2.02112082335745e-12	NA	hypothetical protein
ABZ96998	LEPBI_I0873	0.4659417786	6.485032011	0.0020637531	NA	hypothetical protein
ABZ97018	LEPBI_I0893	0.4653338158	6.6984762996	0.0095540583	NA	putative regulatory protein
ABZ97030	LEPBI_I0905	0.6809643185	6.8601264464	2.31056175421379e-07	NA	putative anti-sigma factor antagonist
ABZ97041	LEPBI_I0917	0.4773756998	6.2952693548	0.0006850977	cheY1	chemotactic two-component response regulator protein CheY
ABZ97070	LEPBI_I0946	0.3176056679	7.9648828691	0.0085635457	fliA	RNA polymerase sigma factor WhiG
ABZ97081	LEPBI_I0957	1.1012455931	9.281950417	3.40542288292167e-05	NA	hypothetical protein
ABZ97143	LEPBI_I1020	0.7142022669	5.9886276183	4.95643261880913e-05	rpmB	50S ribosomal protein L28
ABZ97159	LEPBI_I1036	0.9291929424	5.4535860263	6.85666235719861e-08	NA	putative anti-sigma factor antagonist
ABZ97160	LEPBI_I1037	0.6137566467	5.9680613995	0.0017471752	NA	putative signal peptide
ABZ97250	LEPBI_I1132	0.342328393	7.175594265	0.0063862457	etfA	electron transfer flavoprotein subunit alpha
ABZ97276	LEPBI_I1160	0.487018755	7.0716500746	0.0001173503	NA	hypothetical protein
ABZ97291	LEPBI_I1175	2.1796952693	9.6792875369	1.0723535424186e-14	NA	hypothetical protein
ABZ97296	LEPBI_I1180	0.2821977452	8.8989611081	0.0076977254	cysJ	sulfite reductase flavoprotein subunit alpha
ABZ97313	LEPBI_I1197	0.7143324301	6.483242449	1.89484769695168e-06	NA	putative anti-sigma factor antagonist
ABZ97437	LEPBI_I1327	0.7003918226	6.7889999511	1.27955265645694e-07	NA	hypothetical protein
ABZ97464	LEPBI_I1354	0.5916591726	5.5186133886	0.0076325689	NA	hypothetical protein
ABZ97465	LEPBI_I1355	0.450274531	6.9491788967	0.0020505773	NA	hypothetical protein
ABZ97468	LEPBI_I1358	0.5896007387	7.691889098	2.09752587232849e-05	ahpC2	alkyl hydroperoxide reductase subunit C
ABZ97483	LEPBI_I1374	0.5259074063	7.1912266909	0.0008295216	NA	putative signal peptide
ABZ97539	LEPBI_I1431	0.4320313492	6.6533332517	0.001899946	NA	hypothetical protein
ABZ97567	LEPBI_I1460	0.7605452406	8.4802439142	6.42633480772418e-11	NA	hypothetical protein
ABZ97630	LEPBI_I1523	0.8093042512	6.8410351384	1.88763349075341e-05	NA	hypothetical protein
ABZ97636	LEPBI_I1529	0.2757633926	8.6444776899	0.0078757222	pnp	polynucleotide phosphorylase/polyadenylase
ABZ97660	LEPBI_I1553	0.6223103932	7.5904339659	0.0001490022	NA	hypothetical protein
ABZ97668	LEPBI_I1561	2.2547102072	6.2211385294	3.03753117108509e-13	NA	hypothetical protein
ABZ97671	LEPBI_I1564	1.2813602135	6.2415586079	2.99916068854988e-05	rpsU	30S ribosomal protein S21
ABZ97696	LEPBI_I1589	1.1976007103	8.4983223575	8.84666262432476e-19	flaB	flagellar filament core protein FlaB
ABZ97760	LEPBI_I1653	-0.3308361616	8.7710196891	0.0048006743	NA	histidine kinase sensor protein
ABZ97818	LEPBI_I1712	-0.3335181652	7.6796626338	0.0037172198	NA	hypothetical protein
ABZ97822	LEPBI_I1716	0.4148955931	7.0132458599	0.0028609619	NA	putative transcriptional regulator
ABZ97828	LEPBI_I1722	-0.3425874711	8.4385430108	0.0014010937	asnC	asparaginyl-tRNA synthetase
ABZ97847	LEPBI_I1741	0.6878087418	6.7762049247	1.46583548016082e-07	NA	putative anti-sigma factor antagonist
ABZ97870	LEPBI_I1764	-0.3752435558	8.1274781223	0.0014263515	cheR	chemotaxis protein methyltransferase
ABZ97874	LEPBI_I1768	-0.2726518565	8.8133796008	0.0074527995	NA	hypothetical protein

ABZ97879	LEPBI_I1773	0.4826032361	6.4431406362	0.0020100915	rplU	50S ribosomal protein L21
ABZ97927	LEPBI_I1821	-0.472539901	8.3562674465	0.0013549259	uppP	undecaprenyl-diphosphatase
ABZ97928	LEPBI_I1822	0.3841817286	7.2725099307	0.0051365774	lipL31	hypothetical protein
ABZ97932	LEPBI_I1826	-0.3027845205	8.714221964	0.009654403	NA	hypothetical protein
ABZ97936	LEPBI_I1830	-0.4051462675	8.0790273864	0.0001092322	NA	hypothetical protein
ABZ97977	LEPBI_I1873	0.3797523665	7.4199539177	0.0033553975	NA	OmpA domain-containing protein
ABZ97979	LEPBI_I1875	-0.3481526423	9.0327880324	0.0018214691	NA	putative cyclic-nucleotide-gated cation channel
ABZ97987	LEPBI_I1883	0.497060409	9.1583260814	2.39829787303184e-05	fecA	Iron(III) dicitrate TonB-dependent receptor
ABZ98042	LEPBI_I1939	0.4691771591	7.6291786255	0.0005144007	rpoA	DNA-directed RNA polymerase subunit alpha
ABZ98043	LEPBI_I1940	0.785015116	6.8695709785	1.59943081911331e-08	rpsD	30S ribosomal protein S4
ABZ98044	LEPBI_I1941	0.7709407874	6.0284836945	5.54973572993379e-05	rpsK	30S ribosomal protein S11
ABZ98045	LEPBI_I1942	0.8099274276	7.2382197288	5.1374358951367e-05	rpsM	30S ribosomal protein S13
ABZ98047	LEPBI_I1944	1.2011542675	7.582434287	3.927986482889e-09	adk	adenylate kinase
ABZ98051	LEPBI_I1948	0.6033912619	6.4207941322	0.0002029436	rpsE	30S ribosomal protein S5
ABZ98052	LEPBI_I1949	0.4015120593	6.9013316787	0.004533346	rplR	50S ribosomal protein L18
ABZ98053	LEPBI_I1950	0.445597405	7.9281292974	0.0011316214	rplF	50S ribosomal protein L6
ABZ98055	LEPBI_I1952	0.5296909398	6.8987982903	0.0006287292	rplE	50S ribosomal protein L5
ABZ98058	LEPBI_I1955	0.593869769	5.6680117723	0.0011816936	rpsQ	30S ribosomal protein S17
ABZ98063	LEPBI_I1960	0.5314428839	6.3076127659	0.0009027774	rpsS	30S ribosomal protein S19
ABZ98071	LEPBI_I1968	0.6577493698	7.8629596368	4.65789205245376e-06	rpsG	30S ribosomal protein S7
ABZ98075	LEPBI_I1973	0.7590536473	7.1433181785	7.76496624580802e-05	rplL	50S ribosomal protein L7/L12
ABZ98078	LEPBI_I1976	0.6444300901	6.9254905332	9.53751438765862e-07	rplK	50S ribosomal protein L11
ABZ98079	LEPBI_I1977	0.4134176948	6.5867884018	0.0044034882	nusG	transcription antitermination protein NusG
ABZ98123	LEPBI_I2021	0.3232975628	8.1509533542	0.0087581221	NA	hypothetical protein
ABZ98158	LEPBI_I2056	0.387438407	7.6924002243	0.0009485027	sucC	succinyl-CoA synthetase subunit beta
ABZ98182	LEPBI_I2080	0.3136475403	7.4812934163	0.0080382144	gap	glyceraldehyde-3-phosphate dehydrogenase
ABZ98222	LEPBI_I2120	0.4050618594	6.7631418125	0.0017074462	NA	putative rhodanese-like protein
ABZ98229	LEPBI_I2127	-0.3226402183	7.8895959439	0.0020067108	NA	hypothetical protein
ABZ98234	LEPBI_I2132	0.9818268374	8.0151454016	4.79507813266989e-12	flaB3	flagellar filament 35 kDa core protein
ABZ98235	LEPBI_I2133	0.6662649147	8.7972458296	3.88300280306701e-09	flaB2	flagellar filament 35 kDa core protein
ABZ98245	LEPBI_I2143	0.6511156623	6.1255715526	1.64108949543208e-05	NA	hypothetical protein
ABZ98263	LEPBI_I2162	0.6602805172	5.2229351838	0.0076325689	NA	hypothetical protein
ABZ98277	LEPBI_I2178	0.6736955693	5.8393397543	0.0007441843	NA	hypothetical protein
ABZ98278	LEPBI_I2179	0.5385024109	6.1121609277	0.0022329521	NA	hypothetical protein
ABZ98285	LEPBI_I2186	0.3666009157	6.8836942332	0.0071694094	NA	hypothetical protein
ABZ98291	LEPBI_I2192	0.7115460706	6.0031216541	5.72708546835716e-06	NA	hypothetical protein
ABZ98296	LEPBI_I2197	0.608983689	4.9869206723	0.0064797966	NA	hypothetical protein
ABZ98313	LEPBI_I2215	0.5702868326	5.6534165468	0.002783662	NA	hypothetical protein
ABZ98335	LEPBI_I2239	1.0676042243	5.2054237171	7.74402199945281e-08	NA	hypothetical protein
ABZ98341	LEPBI_I2245	0.5942809811	6.05871683	0.0009459959	NA	hypothetical protein
ABZ98345	LEPBI_I2249	0.9316860953	4.5203356447	1.83069745509135e-05	NA	putative virulence-associated protein B
ABZ98395	LEPBI_I2300	0.4229678583	7.289483981	0.0015202802	trxA2	thioredoxin
ABZ98404	LEPBI_I2309	0.6475666187	6.1759139708	0.000298534	NA	hypothetical protein
ABZ98407	LEPBI_I2312	0.381744625	7.3505483519	0.0031720687	NA	hypothetical protein
ABZ98420	LEPBI_I2327	0.5398806218	8.6892593631	1.91588568281179e-07	NA	putative ATPase
ABZ98421	LEPBI_I2328	0.7262357443	8.0021304043	1.86740245994051e-08	NA	putative anti-sigma factor antagonist
ABZ98427	LEPBI_I2335	0.3329400745	7.4555872494	0.0067659289	flaA1	flagellar filament outer layer protein A
ABZ98429	LEPBI_I2337	0.8827476317	6.9587776068	3.3085705670083e-10	NA	hypothetical protein
ABZ98443	LEPBI_I2352	0.4017207156	7.2196025597	0.0006413758	NA	hypothetical protein
ABZ98456	LEPBI_I2365	0.2884302263	8.6111737674	0.005592818	NA	putative signal peptide
ABZ98466	LEPBI_I2375	0.4060362201	8.1655992764	0.00175989	NA	hemolysin
ABZ98487	LEPBI_I2397	0.6483124138	4.927678441	0.0021674945	NA	hypothetical protein
ABZ98551	LEPBI_I2462	0.6249201675	5.6350417864	0.0002319297	NA	putative signal peptide

ABZ98603	LEPBI_I2518	0.517701659	7.5926052631	9.5023337179605e-05	NA	hypothetical protein
ABZ98605	LEPBI_I2521	-0.3921504799	8.6166309669	0.0029961746	NA	putative phopholipase
ABZ98637	LEPBI_I2556	0.6473384042	4.8420418281	0.0044592063	NA	hypothetical protein
ABZ98639	LEPBI_I2558	0.72051362	4.8158857148	0.0029961746	NA	hypothetical protein
ABZ98666	LEPBI_I2585	0.9645631441	6.1305232359	0.006819283	acpP	acyl carrier protein
ABZ98674	LEPBI_I2595	0.2741966008	8.7488277069	0.0084875894	rpsA	30S ribosomal protein S1
ABZ98697	LEPBI_I2618	0.6508038643	6.8626622771	0.0015202802	NA	putative signal peptide
ABZ98748	LEPBI_I2670	0.6483161483	8.0733538377	1.76961775742407e-06	NA	putative signal peptide
ABZ98752	LEPBI_I2674	0.6418081465	8.153967187	7.90027845084163e-09	lntB	apolipoprotein N-acyltransferase
ABZ98754	LEPBI_I2676	0.8646608676	8.6629562717	3.14168265025901e-12	NA	CarD family transcriptional regulator
ABZ98783	LEPBI_I2705	0.5258404851	7.3673715019	5.54973572993379e-05	NA	hypothetical protein
ABZ98788	LEPBI_I2710	1.1744820453	4.0922227985	0.0006771047	NA	hypothetical protein
ABZ98790	LEPBI_I2712	0.9275820328	4.3194947743	7.76496624580802e-05	NA	hypothetical protein
ABZ98838	LEPBI_I2760	0.3835725286	9.2277980906	0.0001554424	NA	putative TonB-dependent receptor protein
ABZ98839	LEPBI_I2761	0.6824124413	6.307398388	2.72829780708785e-07	NA	putative signal peptide
ABZ98862	LEPBI_I2785	0.5242566095	6.1916277137	9.67450180611628e-05	NA	hypothetical protein
ABZ98912	LEPBI_I2841	0.8901408199	5.3527854204	2.1314202935505e-07	NA	hypothetical protein
ABZ98931	LEPBI_I2862	0.5128673609	10.0298131004	0.00577553	NA	hypothetical protein
ABZ98933	LEPBI_I2864	1.1133684863	8.4763324115	1.32389521021739e-22	NA	hypothetical protein
ABZ99004	LEPBI_I2935	-0.3580039772	8.9862083644	0.0011865691	NA	hypothetical protein
ABZ99093	LEPBI_I3027	0.4883106985	6.1270721741	0.0046847607	NA	TetR family transcriptional regulator
ABZ99192	LEPBI_I3126	0.3291606979	7.5643481771	0.008975755	slyD	FKBP-type peptidyl-prolyl cis-trans isomerase SlyD
ABZ99212	LEPBI_I3147	-0.433293192	7.2745539692	0.004930255	NA	TetR family transcriptional regulator
ABZ99251	LEPBI_I3186	1.9274265643	8.321380916	2.32436652989504e-12	NA	hypothetical protein
ABZ99340	LEPBI_I3275	-0.3040800082	8.0106153339	0.0076325689	metF	5,10-methylenetetrahydrofolate reductase
ABZ99355	LEPBI_I3290	-0.371505068	7.8407484687	0.0031744466	NA	hypothetical protein
ABZ99412	LEPBI_I3348	-0.3504554003	8.6977475258	0.0003038048	NA	hypothetical protein
ABZ99413	LEPBI_I3349	-0.3150684381	8.0586095595	0.0014723753	NA	hypothetical protein
ABZ99423	LEPBI_I3359	-0.4181321956	7.8565517028	0.0015272922	NA	hypothetical protein
ABZ99425	LEPBI_I3361	-0.3590553112	9.4706691295	0.0011559736	NA	hypothetical protein
ABZ99426	LEPBI_I3362	-0.325759062	9.697471866	0.0027906705	NA	TonB-dependent receptor protein
ABZ99427	LEPBI_I3363	-0.4560433223	7.5181215828	1.17395203031626e-05	NA	hypothetical protein
ABZ99428	LEPBI_I3364	-0.2890789834	9.2500335958	0.0063862457	NA	putative outer membrane lipoprotein
ABZ99431	LEPBI_I3367	-0.3861818704	8.6837456051	5.91834924947306e-05	NA	UDP-N- acetylmuramate dehydrogenase
ABZ99432	LEPBI_I3368	-0.3852140036	7.1475286481	0.0050805846	NA	ArsC family arsenate permease
ABZ99435	LEPBI_I3371	-0.3860537844	9.7393362491	0.000303663	NA	hypothetical protein
ABZ99436	LEPBI_I3372	-0.2981137323	9.5545917323	0.0078757222	NA	hypothetical protein
ABZ99437	LEPBI_I3373	-0.3436814821	8.2245152257	0.0011559736	NA	hypothetical protein
ABZ99438	LEPBI_I3374	-0.323665242	8.4062701451	0.009654403	NA	hypothetical protein
ABZ99442	LEPBI_I3378	-0.3638385163	8.9007318993	0.0006665973	dnaJ	chaperone protein DnaJ
ABZ99443	LEPBI_I3379	-0.3533635487	8.8856945882	0.0004703403	dnaK	molecular chaperone DnaK
ABZ99444	LEPBI_I3380	-0.4069242666	7.7915793917	0.0033879689	grpE	heat shock protein GrpE
ABZ99454	LEPBI_I3390	-0.3082109204	8.1716411427	0.002783662	NA	ABC transporter outer membrane protein
ABZ99456	LEPBI_I3392	-0.3781413484	9.6009031817	0.0006530151	NA	hypothetical protein
ABZ99469	LEPBI_I3407	-0.2791945199	8.1299544669	0.009654403	NA	putative chemotactis response regulator
ABZ99470	LEPBI_I3408	-0.359091237	9.0154460416	0.0018214691	aroC	chorismate synthase
ABZ99474	LEPBI_I3412	-0.5476575721	7.8221326514	2.16300522117819e-05	aroD	3-dehydroquinate dehydratase
ABZ99475	LEPBI_I3413	-0.3397920853	9.0633221317	0.0014319329	NA	putative signal peptide
ABZ99476	LEPBI_I3414	-0.3203598856	9.1153782947	0.0066784996	NA	oxidoreductase
ABZ99477	LEPBI_I3415	-0.3916272731	7.6834640598	0.00845019	NA	hypothetical protein
ABZ99479	LEPBI_I3417	-0.4032445596	9.2698148294	0.0013360628	pepN2	aminopeptidase N
ABZ99483	LEPBI_I3422	-0.4409107576	6.853470692	0.0012894481	NA	hypothetical protein
ABZ99494	LEPBI_I3433	-0.3748540924	8.2885852945	0.0010948741	NA	putative signal peptide

ABZ99501	LEPBI_I3440	-0.4061107651	9.6923366252	0.0001070033	hepA	ATP-dependent RNA helicase
ABZ99507	LEPBI_I3447	-0.4136222336	7.7745925292	0.0001432651	NA	hypothetical protein
ABZ99509	LEPBI_I3449	-0.2967528546	8.402440069	0.0081496344	NA	hypothetical protein
ABZ99518	LEPBI_I3458	-0.4002192868	8.0973103331	3.77396197505922e-05	NA	TrmA family RNA methyltransferase
ABZ99520	LEPBI_I3461	-0.4132345778	9.4690485431	9.5023337179605e-05	dnaX1	DNA polymerase III subunit gamma
ABZ99523	LEPBI_I3464	-0.3614108107	8.7616774559	0.0011853288	NA	putative signal peptide
ABZ99526	LEPBI_I3467	-0.4178076272	9.4674946582	0.0022391596	serS	seryl-tRNA synthetase
ABZ99527	LEPBI_I3468	-0.4738666656	8.5530835698	1.01898022776492e-05	NA	putative hydrolase
ABZ99531	LEPBI_I3472	-0.3873762369	9.426563623	0.0005261486	NA	ABC transporter ATP-binding protein
ABZ99532	LEPBI_I3473	-0.3084993805	8.2981378631	0.0043439646	parB	chromosome partitioning protein ParB
ABZ99534	LEPBI_I3475	-0.3099708459	7.7853665576	0.005390319	gidB	glucose-inhibited division protein B
ABZ99536	LEPBI_I3477	-0.3749634578	9.4367798935	0.0005144007	gidA	tRNA uridine 5-carboxymethylaminomethyl modification protein GidA
ABZ99538	LEPBI_I3479	-0.4642040667	7.5343536677	0.0007441843	dnaX2	DNA polymerase III subunit gamma/tau
ABZ99546	LEPBI_II0008	-0.3044908751	8.846672758	0.0033879689	NA	Histidine kinase sensor protein
ABZ99550	LEPBI_II0012	-0.3305023627	7.9400143985	0.0006298511	batA	von Willebrand factor A
ABZ99552	LEPBI_II0014	-0.3635262641	7.2958062658	0.0023792307	NA	hypothetical protein
ABZ99553	LEPBI_II0015	-0.5555213135	9.7966742459	0.0021508851	NA	methanol dehydrogenase regulator (MoxR)
ABZ99557	LEPBI_II0019	-0.5013035646	6.142125097	0.0062315272	NA	signal peptide
ABZ99559	LEPBI_II0021	-0.3273172442	7.9326448698	0.0050330915	ndh	NADH dehydrogenase
ABZ99564	LEPBI_II0026	-0.387284534	7.3549049373	0.0012658474	NA	chromosome partitioning protein ParB
ABZ99570	LEPBI_II0032	-0.3226421312	8.5180412867	0.0033553975	NA	hypothetical protein
ABZ99573	LEPBI_II0035	-0.3848581304	8.5963102811	0.0002294211	NA	hypothetical protein
ABZ99577	LEPBI_II0039	-0.4317628792	7.4443177621	0.0004858284	NA	hypothetical protein
ABZ99578	LEPBI_II0040	-0.4095996349	8.099679672	1.85479332904623e-05	NA	hypothetical protein
ABZ99579	LEPBI_II0041	-0.2773366185	8.5020577941	0.009654403	glpD	glycerol-3-phosphate oxidase
ABZ99586	LEPBI_II0048	-0.2961652863	8.5217379995	0.0068466741	aspA	Aspartate ammonia-lyase (aspartase)
ABZ99593	LEPBI_II0055	-0.4889922716	6.6825737446	0.0023521999	NA	hypothetical protein
ABZ99594	LEPBI_II0056	-0.3789899424	7.4726472056	0.0006501991	NA	hypothetical protein
ABZ99596	LEPBI_II0058	-0.3017289464	9.1681648556	0.0085653983	NA	P-type ATPase, copper transporting ATPase, a phophatase-like domain
ABZ99598	LEPBI_II0060	-0.3348220315	7.720393719	0.0077450449	NA	iron-sulfur cluster-binding protein; transmembrane protein
ABZ99602	LEPBI_II0064	-0.3931432569	8.2613831628	6.69150719194611e-06	NA	cbb3-type cytochrome c oxidase subunit I
ABZ99604	LEPBI_II0066	-0.2856379733	8.1115591169	0.002908963	gltD	glutamate synthase (NADPH)
ABZ99613	LEPBI_II0075	-0.3139760066	8.4737579511	0.0028384439	traB	Pheromone shutdown protein, TraB family
ABZ99619	LEPBI_II0081	-0.4029789389	6.6193324058	0.0032523022	NA	signal peptide
ABZ99622	LEPBI_II0084	-0.5189622304	8.7802207034	0.0004781311	NA	MotA/TolQ/ExbB proton channel protein
ABZ99623	LEPBI_II0085	-0.5206256185	8.7527155181	8.62005520862355e-09	NA	hypothetical protein
ABZ99624	LEPBI_II0087	-0.5164292732	10.0395416751	3.70753058017549e-06	NA	acriflavine resistance protein D; transmembrane protein
ABZ99625	LEPBI_II0088	-0.5290373113	7.0127542183	9.91446625630244e-05	NA	hypothetical protein
ABZ99627	LEPBI_II0090	-0.3985813059	6.8225609576	0.008154375	NA	response regulatory protein
ABZ99630	LEPBI_II0094	-0.3145397555	9.0870864192	0.0047792244	mutB	methylmalonyl-COA mutase large subunit
ABZ99636	LEPBI_II0100	-0.4116015112	7.9607930649	3.40542288292167e-05	NA	hypothetical protein
ABZ99637	LEPBI_II0101	-0.544192899	8.1163761914	1.32262705794507e-08	NA	hypothetical protein
ABZ99643	LEPBI_II0107	-0.5497794033	8.4716290265	5.91834924947306e-05	NA	hypothetical protein
ABZ99645	LEPBI_II0109	-0.4705694741	8.2869700547	2.6245815773568e-07	NA	hypothetical protein
ABZ99646	LEPBI_II0110	-0.4002650537	8.1725594262	5.60163502933227e-05	pyrF	Orotidine-5'-monophosphate decarboxylase
ABZ99647	LEPBI_II0111	-0.4123139504	8.3859053142	5.17330300916011e-05	NA	dehydrogenase
ABZ99649	LEPBI_II0113	-0.5674751913	9.1160443759	7.71276280448329e-06	cfa	Cyclopropane-fatty-acyl-phospholipid synthase
ABZ99653	LEPBI_II0117	-0.3480574376	9.0595463636	0.0015852622	NA	signal peptide
ABZ99657	LEPBI_II0121	-0.4346819422	6.5363239245	0.004930255	NA	Dolichyl-phosphate beta-D-mannosyltransferase
ABZ99661	LEPBI_II0125	-0.4758101063	8.355063544	6.95339394839553e-07	htrA1	HtrA1; signal peptide
ABZ99665	LEPBI_II0129	-0.2957721273	8.3407545591	0.0079732126	NA	permease; transmembrane protein
ABZ99669	LEPBI_II0133	-0.3783479224	9.1816905984	0.0003522414	NA	helicase, ATP-dependent
ABZ99671	LEPBI_II0135	-0.3543538884	8.0229748715	0.0011965073	NA	hypothetical protein

ABZ99673	LEPBI_II0138	-0.5461933237	8.2848587832	1.19325371276709e-06	NA	hypothetical protein
ABZ99680	LEPBI_II0145	-0.3179354572	9.4306362124	0.0055750785	NA	adenylate/guanylate cyclase
ABZ99681	LEPBI_II0146	-0.3406895939	8.5248328123	0.0011853288	NA	peroxidase
ABZ99683	LEPBI_II0148	-0.4533444242	7.7275686846	0.000139877	NA	hypothetical protein
ABZ99684	LEPBI_II0149	-0.5109758755	7.8067718605	3.60462839824744e-06	NA	hypothetical protein
ABZ99692	LEPBI_II0157	-0.340723336	8.2642200335	0.0017335867	NA	4-coumarate--CoA ligase
ABZ99695	LEPBI_II0160	-0.3806872131	8.2308799345	4.26109554257761e-05	NA	hypothetical protein
ABZ99697	LEPBI_II0162	-0.328675823	9.2197858187	0.0072404349	NA	hypothetical protein
ABZ99701	LEPBI_II0166	-0.4038974728	8.522443325	0.0006182772	csdB	Cysteine desulfurase
ABZ99703	LEPBI_II0168	-0.3287123064	7.2301877943	0.0071602703	NA	hypothetical protein
ABZ99704	LEPBI_II0169	-0.4842459835	7.3511983529	0.0005047053	NA	N-carbamoyl-D-amino acid hydrolase
ABZ99705	LEPBI_II0170	-0.5990106708	7.9432972941	2.71900577311796e-07	argB	acetylglutamate kinase
ABZ99706	LEPBI_II0171	-0.4927402649	9.4449306993	9.43452255170303e-06	NA	Sigma factor SigB regulation protein RsbU; phosphoserine phosphatase
ABZ99708	LEPBI_II0173	-0.3873917002	10.0869932371	0.000943029	czcA	RND divalent metal cation efflux transporter
ABZ99709	LEPBI_II0174	-0.4009979951	7.8848817763	9.93753373093714e-05	NA	hypothetical protein
ABZ99712	LEPBI_II0179	-0.3239611719	8.5189217465	0.004681717	NA	diphosphate--fructose-6-phosphate 1-phosphotransferase
ABZ99716	LEPBI_II0183	-0.3143557952	9.5726762034	0.004533346	metH	B12-dependent methionine synthase
ABZ99720	LEPBI_II0187	-0.3236886386	9.1459851016	0.0018070318	NA	methyl-accepting chemotaxis protein TlpA
ABZ99721	LEPBI_II0188	-0.4022486438	8.3365352098	9.91446625630244e-05	NA	outermembrane protein
ABZ99722	LEPBI_II0189	-0.503676274	7.7926973938	5.04508219133025e-06	NA	drug/metabolite exporter
ABZ99725	LEPBI_II0192	-0.5004246557	6.6390394899	0.0017037483	NA	hypothetical protein
ABZ99726	LEPBI_II0193	-0.3950116583	9.2828542905	7.56588434837131e-05	NA	methyl-accepting chemotaxis protein; signal peptide
ABZ99727	LEPBI_II0194	-0.3463713039	9.1690459285	0.0006079672	NA	methyl-accepting chemotaxis protein; signal peptide
ABZ99728	LEPBI_II0195	-0.2560790772	8.3109541411	0.0083797622	NA	hypothetical protein
ABZ99731	LEPBI_II0198	-0.4542667589	8.1119952872	7.79334221119504e-07	NA	short-chain dehydrogenase/reductase SDR
ABZ99732	LEPBI_II0199	-0.3903450394	6.8544520113	0.0043517033	NA	3-oxoacyl-[acyl-carrier protein] reductase
ABZ99735	LEPBI_II0202	-0.4210142479	8.5894121594	0.0001596724	kamA	L-lysine 2,3-aminomutase
ABZ99736	LEPBI_II0203	-0.3839232104	6.8976025943	0.0017503402	NA	signal peptide
ABZ99740	LEPBI_II0207	-0.2793411951	8.533296325	0.0078757222	NA	two-component sensor histidine kinase
ABZ99741	LEPBI_II0208	-0.2997760806	8.5759097626	0.0055811374	deaD	DEAD/DEAH box helicase
ABZ99742	LEPBI_II0209	-0.3912451483	8.2018907443	0.0003476512	NA	cyclopropane-fatty-acyl-phospholipid synthase
ABZ99743	LEPBI_II0210	-0.4046302968	7.7680262763	0.0001416607	NA	hypothetical protein
ABZ99744	LEPBI_II0211	-0.3807459573	7.2969692844	0.0026456286	NA	3-oxoacyl-[acyl-carrier protein] reductase
ABZ99745	LEPBI_II0212	-0.3482961249	7.5141099756	0.0048006743	NA	Alcohol dehydrogenase (Zinc-containing alcohol dehydrogenase family)
ABZ99748	LEPBI_II0215	-1.2472755709	6.8462999763	7.35602908549769e-10	NA	hypothetical protein
ABZ99752	LEPBI_II0219	-0.5312022356	6.3418600619	0.001036133	NA	hypothetical protein
ABZ99753	LEPBI_II0220	-0.3076922665	8.7027765976	0.003012724	NA	hypothetical protein
ABZ99757	LEPBI_II0224	-0.4348459309	8.103866841	5.60163502933227e-05	NA	hypothetical protein
ABZ99758	LEPBI_II0225	-0.4631875691	6.1291112402	0.0074247643	NA	hypothetical protein
ABZ99759	LEPBI_II0226	-0.3291590041	8.0751203944	0.0019125232	NA	hypothetical protein
ABZ99760	LEPBI_II0227	-0.3323610579	7.8648316161	0.0053248034	NA	hypothetical protein
ABZ99765	LEPBI_II0232	-0.5785177502	7.3035258189	2.43357386577175e-06	NA	signal peptide
ABZ99769	LEPBI_II0236	-0.4048360572	6.7948499739	0.0042920489	NA	hypothetical protein
ABZ99781	LEPBI_II0248	-0.3929791218	8.498409416	0.000815605	NA	hypothetical protein
ABZ99783	LEPBI_II0250	-0.5952672345	7.247079387	2.5066038931868e-06	NA	hypothetical protein
ABZ99786	LEPBI_II0253	-0.3967688672	7.6061416954	0.0063862457	NA	hypothetical protein
ABZ99787	LEPBI_II0254	-0.3796268657	8.291837379	9.93753373093714e-05	NA	glycosyl transferase
ABZ99788	LEPBI_II0255	-0.4273808737	6.4791615574	0.002487851	NA	hypothetical protein
ABZ99789	LEPBI_II0256	-0.3275360481	8.6661173164	0.0055764542	NA	hypothetical protein
ABZ99795	LEPBI_II0262	-0.4399436877	9.1438382331	3.3857679122078e-06	NA	hypothetical protein
ABZ99796	LEPBI_II0263	-0.3268040058	7.9185953632	0.0049370219	NA	hypothetical protein
ABZ99798	LEPBI_II0265	-0.4491618035	9.2141080383	0.0001723272	NA	methyl-accepting chemotaxis protein
ABZ99801	LEPBI_II0268	-0.2876941391	8.8391420368	0.0048478149	NA	C-terminal processing peptidase

ABZ99809	LEPBI_II0276	-0.3343672794	8.5144601834	0.0015852622	NA	hypothetical protein
ABZ99810	LEPBI_II0277	-0.388390655	8.724654322	0.0001226758	algI	alginate O-acetyltransferase
ABZ99823	LEPBI_p0009	1.0118423181	4.6961934405	1.56039193023144e-05	NA	hypothetical protein
ABZ99826	LEPBI_p0012	0.6177657011	6.6735418498	0.0005847176	hemS	Hemin degradation protein HemS

Table S5 (cont.). Differentially expressed genes in BvsP_120.

Gene	Synonym	logFC	logCPM	FDR	Symbol	Product
ABZ96158	LEPBI_I0011	-0.5004827861	6.9621288105	0.0003063382	NA	putative transcriptional regulator
ABZ96162	LEPBI_I0015	-0.3587948725	6.7063102854	0.0044132317	NA	hypothetical protein
ABZ96187	LEPBI_I0040	-0.4258958458	6.8902257033	0.0002947639	NA	putative signal peptide
ABZ96222	LEPBI_I0075	-0.3450421164	7.2247282366	0.0028411542	NA	putative nucleotide-diphospho-sugar transferase
ABZ96228	LEPBI_I0081	-0.5770808538	6.1539470333	0.0020740618	NA	putative response regulator
ABZ96239	LEPBI_I0092	-0.2675080294	8.8887410313	0.0058228895	pyrD	dihydroorotate dehydrogenase 2
ABZ96257	LEPBI_I0110	-0.7723418584	8.2890135629	5.83934591985084e-14	NA	hypothetical protein
ABZ96261	LEPBI_I0114	-0.3763911911	8.0396991711	0.0054813602	NA	sodium/bile acid cotransporter family protein
ABZ96262	LEPBI_I0115	-0.478537662	5.8445996291	0.0043997932	NA	hypothetical protein
ABZ96271	LEPBI_I0124	-0.6278125557	6.6095732391	5.05592469442429e-05	NA	hypothetical protein
ABZ96278	LEPBI_I0132	-0.7603864466	5.5513463118	6.51490026298288e-06	NA	hypothetical protein
ABZ96287	LEPBI_I0142	-0.3477810861	7.9013797053	0.0076034178	glpF	glycerol uptake facilitator protein
ABZ96289	LEPBI_I0144	2.0605681113	7.8637143239	9.16952420795051e-09	NA	hypothetical protein
ABZ96292	LEPBI_I0147	-0.3491975588	7.6642546583	0.0028739347	exbD2	biopolymer transport ExbD protein
ABZ96299	LEPBI_I0154	-0.2720856946	8.3959859272	0.0059546873	add1	adenosine deaminase
ABZ96307	LEPBI_I0162	-0.4341443762	7.7183706169	2.32521122475938e-05	NA	hypothetical protein
ABZ96320	LEPBI_I0175	-0.3622632459	7.111661173	0.0014457866	NA	hypothetical protein
ABZ96336	LEPBI_I0191	-0.4827935024	5.706934936	0.0053230573	NA	hypothetical protein
ABZ96341	LEPBI_I0196	-0.3536632561	8.0592918224	0.0003850803	motA	chemotaxis MotA protein
ABZ96342	LEPBI_I0197	-0.3742507747	7.668812202	0.0003063382	motB	flagellar motor protein MotB
ABZ96356	LEPBI_I0211	-0.2559880835	8.0495632299	0.0082860263	NA	hypothetical protein
ABZ96383	LEPBI_I0238	1.0912475533	8.4718942078	1.71386370161502e-05	ispE	4-diphosphocytidyl-2-C-methyl-D-erythritol kinase
ABZ96387	LEPBI_I0242	-0.2910082313	8.8705302779	0.0016486715	NA	putative sodium:solute symporter
ABZ96391	LEPBI_I0246	-0.2921871022	7.7471056402	0.0029818936	NA	hypothetical protein
ABZ96392	LEPBI_I0247	-0.3729320751	8.194246796	4.49997988723769e-05	NA	hypothetical protein
ABZ96400	LEPBI_I0255	-0.5295681042	7.440287296	6.59519972216601e-07	NA	hypothetical protein
ABZ96412	LEPBI_I0267	0.454858211	9.2729552605	0.007102486	NA	putative signal peptide
ABZ96419	LEPBI_I0275	2.3398462799	8.7558557537	9.98482187635889e-06	NA	hypothetical protein
ABZ96420	LEPBI_I0276	-0.4102508853	7.9624156938	0.0001140561	NA	hypothetical protein
ABZ96421	LEPBI_I0277	0.3731184526	7.6638244092	0.0021816041	NA	hypothetical protein
ABZ96433	LEPBI_I0289	0.879161184	7.5568783639	0.0003898781	NA	hypothetical protein
ABZ96445	LEPBI_I0301	-0.506559252	5.3720011992	0.0028411542	NA	hypothetical protein
ABZ96457	LEPBI_I0313	0.7490871993	8.6493150698	3.04545470130188e-11	NA	putative signal peptide
ABZ96471	LEPBI_I0327	-0.2755210124	8.1320723898	0.0093172659	dppC	ABC transporter permease
ABZ96508	LEPBI_I0365	-0.33171967	7.548560294	0.0017562659	NA	putative negative transcriptional regulator
ABZ96511	LEPBI_I0368	-0.3960663084	6.6345834706	0.0024131652	NA	hypothetical protein
ABZ96523	LEPBI_I0381	-0.516444054	5.4813042618	0.0032052843	NA	hypothetical protein
ABZ96546	LEPBI_I0404	0.5753770984	8.561895118	3.66677906967842e-07	NA	hypothetical protein
ABZ96573	LEPBI_I0431	-0.5516027611	8.1405417145	0.007671178	NA	hypothetical protein
ABZ96627	LEPBI_I0489	-0.3224102994	8.4841156601	0.0003532647	NA	hypothetical protein
ABZ96653	LEPBI_I0515	0.5404153607	7.635377771	0.0005830755	rpsT	30S ribosomal protein S20
ABZ96661	LEPBI_I0523	-0.3669409155	8.9086831264	0.0001140561	NA	putative serine-type endopeptidase
ABZ96669	LEPBI_I0531	0.4566256608	7.0787833788	0.000892425	NA	hypothetical protein
ABZ96670	LEPBI_I0532	0.4592877715	8.1171928644	0.0007819714	NA	putative signal peptide
ABZ96675	LEPBI_I0537	0.4154387388	9.008073391	5.20831692174193e-05	NA	hypothetical protein
ABZ96676	LEPBI_I0538	0.2703612702	8.9713009829	0.0054813602	NA	putative FAD-linked oxidase
ABZ96699	LEPBI_I0561	0.4381827826	9.1667038655	2.01789277718986e-05	NA	hypothetical protein
ABZ96722	LEPBI_I0584	0.3608365597	8.0343663942	0.0002324256	NA	putative S-adenosyl-L-methionine-dependent methyltransferase
ABZ96727	LEPBI_I0590	0.5215733151	5.8478277183	0.0027790524	NA	hypothetical protein
ABZ96784	LEPBI_I0651	-0.2745219262	8.1363724317	0.0044505911	NA	hypothetical protein

ABZ96815	LEPBI_I0683	0.494760358	7.9980262479	1.31822210999569e-05	NA	putative lipoprotein
ABZ96824	LEPBI_I0692	-0.3516587097	6.7954754043	0.0098173821	NA	hypothetical protein
ABZ96830	LEPBI_I0698	0.286532168	7.9643716776	0.0092497318	NA	hypothetical protein
ABZ96853	LEPBI_I0721	0.3385683779	8.1684107458	0.0046665043	NA	putative NAD-dependent deacetylase
ABZ96895	LEPBI_I0764	-0.3153907617	8.6616390086	0.0010813264	pykF	pyruvate kinase
ABZ96899	LEPBI_I0768	-0.45892787	8.0289807663	1.27855305053055e-07	NA	hypothetical protein
ABZ96912	LEPBI_I0782	-0.2959771927	8.1573744179	0.0011561945	NA	hypothetical protein
ABZ96913	LEPBI_I0783	-0.2660955881	8.3025210536	0.0084581508	NA	hypothetical protein
ABZ96919	LEPBI_I0789	0.3797501448	8.36000227	0.0011561945	NA	hypothetical protein
ABZ96930	LEPBI_I0802	0.537193164	7.8346278004	1.03041521325514e-05	atpE	ATP synthase F0 subunit C
ABZ96932	LEPBI_I0804	0.4181898303	7.7343555693	0.0017643161	atpH	F0F1 ATP synthase subunit delta
ABZ96933	LEPBI_I0805	0.2766371152	8.8001101711	0.0076123973	atpA	F0F1 ATP synthase subunit alpha
ABZ96935	LEPBI_I0807	0.2812641385	8.7187025318	0.0084065323	atpD	F0F1 ATP synthase subunit beta
ABZ96944	LEPBI_I0816	-0.4162261386	7.1444819117	0.0010813264	NA	hypothetical protein
ABZ96945	LEPBI_Ia0817	0.3080332801	8.9245698738	0.0030279285	ompL1	outer membrane protein
ABZ96962	LEPBI_I0835	0.4746802818	7.7051666859	7.57367514275877e-05	NA	TetR family transcriptional regulator
ABZ96966	LEPBI_I0839	0.4011399063	7.7460831614	7.66348856414957e-05	NA	putative glycosyl transferase
ABZ96976	LEPBI_I0849	-0.2812566533	8.5707959044	0.0046119217	NA	ABC transporter ATP binding protein/permease
ABZ96986	LEPBI_I0859	0.5178020285	8.5895762377	4.0277829735504e-05	NA	hypothetical protein
ABZ96994	LEPBI_I0869	0.4288205961	7.7972620919	9.96548764614356e-05	NA	hypothetical protein
ABZ96996	LEPBI_I0871	0.3346437356	8.3998819939	0.0003898781	NA	CRP family transcriptional regulator
ABZ97010	LEPBI_I0885	0.5331205018	9.3602059476	0.0029888831	NA	hypothetical protein
ABZ97026	LEPBI_I0901	0.421393682	9.3535406276	5.15212416420026e-05	NA	two-component sensor protein
ABZ97029	LEPBI_I0904	0.4340502882	7.8713621332	0.0001345808	NA	putative sigma factor
ABZ97030	LEPBI_I0905	0.7142772435	6.8601264464	2.12125592542439e-07	NA	putative anti-sigma factor antagonist
ABZ97039	LEPBI_I0915	0.3285666032	8.837961437	0.0005246082	NA	putative two-component sensor molecule
ABZ97058	LEPBI_I0934	0.4793919601	5.6268364943	0.0082206676	NA	hypothetical protein
ABZ97075	LEPBI_I0951	0.4804869892	7.3313405598	0.0009975244	gmk	guanylate kinase
ABZ97081	LEPBI_I0957	2.1282336693	9.281950417	1.32477852136588e-18	NA	hypothetical protein
ABZ97098	LEPBI_I0974	0.3885846048	7.1308126772	0.0082206676	tatA	Sec-independent protein secretion pathway component TatA/E
ABZ97100	LEPBI_I0976	0.2505494867	8.210910781	0.0095365389	NA	putative signal peptide
ABZ97150	LEPBI_I1027	-0.5928395646	7.5107175096	1.04735356354059e-05	NA	hypothetical protein
ABZ97198	LEPBI_I1075	0.2473972456	8.2093778511	0.0082206676	NA	putative methyl-accepting chemotaxis protein
ABZ97205	LEPBI_I1084	0.4186248952	8.329559309	1.42373415945619e-06	NA	hypothetical protein
ABZ97250	LEPBI_I1132	0.348075466	7.175594265	0.0068590506	etfA	electron transfer flavoprotein subunit alpha
ABZ97266	LEPBI_I1150	-0.3634332474	7.8572688471	0.0025507974	NA	putative cAMP binding protein
ABZ97287	LEPBI_I1171	0.4834331976	8.7129464391	3.43353500980654e-07	hemA	glutamyl-tRNA reductase
ABZ97291	LEPBI_I1175	0.9858684154	9.6792875369	0.0005964039	NA	hypothetical protein
ABZ97294	LEPBI_I1178	0.3051613478	7.9068666418	0.0052089101	sirA	siroheme synthase
ABZ97304	LEPBI_I1188	0.4146898436	8.3563120906	5.15212416420026e-05	sbp	sulfate ABC transporter substrate-binding protein
ABZ97313	LEPBI_I1197	0.444341838	6.483242449	0.0074919595	NA	putative anti-sigma factor antagonist
ABZ97333	LEPBI_I1219	0.3295528847	8.3426694355	0.0009255343	NA	putative sensory transduction histidine kinase
ABZ97345	LEPBI_I1231	0.27992056	8.571468184	0.0029818936	NA	hypothetical protein
ABZ97353	LEPBI_I1240	0.4196629143	7.4850084556	0.0010727767	NA	putative dihydrofolate reductase
ABZ97358	LEPBI_I1245	0.3637326605	7.7018733743	0.002922699	NA	hypothetical protein
ABZ97362	LEPBI_I1250	-0.4936634238	7.2146052061	0.0001541092	NA	hypothetical protein
ABZ97364	LEPBI_I1252	0.3736896718	8.5399821618	2.24214159230944e-05	NA	putative signal peptide
ABZ97388	LEPBI_I1278	0.2752814022	8.4014578635	0.0094625268	NA	hypothetical protein
ABZ97396	LEPBI_I1286	-0.5381504538	6.810047695	0.0011303821	NA	hypothetical protein
ABZ97414	LEPBI_I1304	0.6098169372	5.4819084829	0.0046119738	nuoK	NADH-quinone oxidoreductase subunit K
ABZ97427	LEPBI_I1317	0.6335904967	7.6509028855	0.0086715178	NA	hypothetical protein
ABZ97431	LEPBI_I1321	0.6351426829	6.2756291366	1.36761458390032e-05	NA	putative anti-sigma factor antagonist
ABZ97449	LEPBI_I1339	-0.2801752521	7.9096627658	0.005248281	NA	hypothetical protein

ABZ97468	LEPBI_I1358	0.4947536749	7.691889098	0.0002911909	ahpC2	alkyl hydroperoxide reductase subunit C
ABZ97477	LEPBI_I1368	0.4190845298	7.2820459311	0.0002753902	NA	putative cytochrome c3
ABZ97541	LEPBI_I1433	-0.3206829803	8.2793048531	0.0006993374	NA	hypothetical protein
ABZ97553	LEPBI_I1445	0.5256174398	7.0961037277	1.00111261317932e-05	NA	hypothetical protein
ABZ97567	LEPBI_I1460	0.4462248886	8.4802439142	0.00023718	NA	hypothetical protein
ABZ97570	LEPBI_I1463	-0.3972696968	7.86280289	3.82061579241368e-05	NA	putative ribosomal subunit pseudouridine synthase
ABZ97604	LEPBI_I1497	0.4923235703	7.4631599078	7.21534581289544e-05	NA	ABC transporter ATP-binding protein
ABZ97608	LEPBI_I1501	-0.3344710383	6.6395552255	0.0084714983	ruvA	Holliday junction DNA helicase RuvA
ABZ97630	LEPBI_I1523	0.7477214656	6.8410351384	7.21534581289544e-05	NA	hypothetical protein
ABZ97645	LEPBI_I1538	-0.3774496687	7.9163682894	0.0006088059	NA	putative S-adenosyl-L-methionine-dependent methyltransferase
ABZ97668	LEPBI_I1561	1.2748213282	6.2211385294	7.86768133727318e-05	NA	hypothetical protein
ABZ97671	LEPBI_I1564	1.69573447	6.2415586079	1.37893679685547e-08	rpsU	30S ribosomal protein S21
ABZ97681	LEPBI_I1574	-0.7250295129	6.3430604353	1.62852735964469e-08	NA	hypothetical protein
ABZ97682	LEPBI_I1575	-0.2613592897	8.8403062091	0.0090648343	NA	putative phosphodiesterase or phosphatase
ABZ97700	LEPBI_I1593	-0.2707961524	8.3438819231	0.0030496603	metY	O-acetylhomoserine sulfhydrylase
ABZ97703	LEPBI_I1596	-0.4279671769	7.0980862131	0.0056927529	NA	hypothetical protein
ABZ97732	LEPBI_I1625	-0.3145306344	8.0190484779	0.0010231909	leuC2	isopropylmalate isomerase large subunit
ABZ97744	LEPBI_I1637	-0.3061483011	7.6216152918	0.0079209101	NA	putative glycosyltransferase
ABZ97790	LEPBI_I1683	-0.311385845	8.3825894498	0.0028739347	ftsA2	cell division protein FtsA
ABZ97796	LEPBI_I1689	-0.9441102863	5.3304241323	1.20914933729027e-06	NA	hypothetical protein
ABZ97806	LEPBI_I1700	-0.3699569159	6.4361794058	0.0080789211	NA	hypothetical protein
ABZ97820	LEPBI_I1714	0.3492968363	6.8304671701	0.0091397466	NA	hypothetical protein
ABZ97847	LEPBI_I1741	0.4062902193	6.7762049247	0.0082860263	NA	putative anti-sigma factor antagonist
ABZ97862	LEPBI_I1756	-0.2648163748	8.6740266574	0.0059546873	murC	UDP-N-acetylmuramate--L-alanine ligase
ABZ97865	LEPBI_I1759	-0.3602269757	7.4184519401	0.0008725906	mraY	phospho-N-acetylmuramoyl-pentapeptide-transferase
ABZ97870	LEPBI_I1764	0.3250714117	8.1274781223	0.005345247	cheR	chemotaxis protein methyltransferase
ABZ97873	LEPBI_I1767	-0.2883494105	8.7726226654	0.0074994184	NA	ammonium ABC transporter permease
ABZ97875	LEPBI_I1769	-0.2697187001	8.3331070104	0.0079615906	NA	hypothetical protein
ABZ97880	LEPBI_I1774	0.5190850854	6.9144571884	0.0018086298	NA	putative ribosomal protein
ABZ97885	LEPBI_I1779	0.5630459199	6.4327019678	0.0001003934	NA	putative nicotinate-nucleotide adenylyltransferase
ABZ97888	LEPBI_I1782	-0.5176340675	5.9732500918	0.0010231909	NA	hypothetical protein
ABZ97898	LEPBI_I1792	-0.3184330466	7.2403314206	0.0041482571	NA	hypothetical protein
ABZ97924	LEPBI_I1818	0.2637419295	8.4685618137	0.0051896523	NA	putative poly(beta-D-mannuronate) O-acetylase
ABZ97939	LEPBI_I1833	-0.2756064688	7.7839539972	0.0062129536	NA	hypothetical protein
ABZ97942	LEPBI_I1836	-0.3164687121	7.9762608372	0.0040007243	NA	putative acyl-CoA dehydrogenase
ABZ97950	LEPBI_I1844	-0.2651486803	8.0886165341	0.007029352	NA	hypothetical protein
ABZ97951	LEPBI_I1845	-0.3169443502	8.0356859749	0.0005832955	leuA	2-isopropylmalate synthase
ABZ97952	LEPBI_I1846	-0.3200925371	7.3132920096	0.0029818936	hisC	histidinol-phosphate aminotransferase
ABZ97963	LEPBI_I1859	-0.3305019559	7.3813533047	0.0051383982	NA	putative signal peptide
ABZ98012	LEPBI_I1909	0.9560629248	3.8758311372	0.0060952823	NA	putative BolA-like protein
ABZ98042	LEPBI_I1939	0.3985913007	7.6291786255	0.0035279915	rpoA	DNA-directed RNA polymerase subunit alpha
ABZ98046	LEPBI_I1943	1.1511826645	7.0649249356	1.6501570143038e-13	infA	translation initiation factor IF-1
ABZ98047	LEPBI_I1944	0.7265295898	7.582434287	0.0005269942	adk	adenylate kinase
ABZ98071	LEPBI_I1968	0.5299191662	7.8629596368	0.0001941983	rpsG	30S ribosomal protein S7
ABZ98106	LEPBI_I2004	0.3471916465	8.9576062915	0.0092592744	NA	hypothetical protein
ABZ98145	LEPBI_I2043	0.3126561593	8.1802768376	0.0010033301	NA	putative acyltransferase
ABZ98172	LEPBI_I2070	0.4931074515	8.6856363965	6.59519972216601e-07	NA	hypothetical protein
ABZ98202	LEPBI_I2100	0.5738763781	6.9863550498	9.22991131858375e-05	rpsF	30S ribosomal protein S6
ABZ98229	LEPBI_I2127	0.4170548039	7.8895959439	1.16376698335723e-05	NA	hypothetical protein
ABZ98268	LEPBI_I2168	0.5543056795	6.0291875888	0.0013557755	NA	hypothetical protein
ABZ98276	LEPBI_I2177	0.3710547763	7.5113037713	0.0013434707	NA	hypothetical protein
ABZ98278	LEPBI_I2179	-0.5363296371	6.1121609277	0.0009723839	NA	hypothetical protein
ABZ98292	LEPBI_I2193	0.3260501374	8.9838272836	0.0008725906	NA	hypothetical protein

ABZ98316	LEPBI_I2218	0.4281935102	6.6042303073	0.0097058514	NA	putative transcriptional regulator
ABZ98326	LEPBI_I2229	-0.3930152313	6.7546200893	0.0038591894	NA	hypothetical protein
ABZ98331	LEPBI_I2234	-0.6378351665	4.9382457877	0.0058228895	NA	hypothetical protein
ABZ98375	LEPBI_I2280	0.4168751426	7.1885242585	0.0037450434	NA	hypothetical protein
ABZ98395	LEPBI_I2300	0.3936334659	7.289483981	0.0040732073	trxA2	thioredoxin
ABZ98453	LEPBI_I2362	0.2456271308	8.7556402926	0.0091277516	ilvB2	acetolactate synthase large subunit
ABZ98462	LEPBI_I2371	-1.7616960196	4.6391423933	8.38129239825869e-17	NA	hypothetical protein
ABZ98533	LEPBI_I2444	0.325556606	7.8670450967	0.0039383443	NA	hypothetical protein
ABZ98584	LEPBI_I2497	0.3402544689	9.5188267052	0.0012687027	ppc	phosphoenolpyruvate carboxylase
ABZ98591	LEPBI_I2505	-0.4636956631	5.9428906308	0.0029818936	NA	hypothetical protein
ABZ98625	LEPBI_I2544	0.2723654722	9.2133021353	0.0054813602	NA	putative DNA repair helicase
ABZ98666	LEPBI_I2585	0.9720659576	6.1305232359	0.0052369147	acpP	acyl carrier protein
ABZ98680	LEPBI_I2601	0.3291577532	7.5127624705	0.005248281	NA	hypothetical protein
ABZ98689	LEPBI_I2610	0.4009911346	8.9900237414	6.36544411039116e-06	NA	membrane-associated Zn-dependent metalloprotease
ABZ98692	LEPBI_I2613	-0.4420167029	5.7952043528	0.0082206676	uppS	undecaprenyl pyrophosphate synthase
ABZ98697	LEPBI_I2618	0.6874555375	6.8626622771	0.0003898781	NA	putative signal peptide
ABZ98708	LEPBI_I2629	0.6197047826	7.6663611145	8.00741067821248e-07	NA	rhodanese-like domain-containing protein
ABZ98709	LEPBI_I2630	-0.2982844614	8.00753974	0.0059546873	NA	putative hydroxyacylglutathione hydrolase
ABZ98712	LEPBI_I2633	0.4070903933	8.0872755338	0.00098092	msrA2	peptide methionine sulfoxide reductase MsrA
ABZ98724	LEPBI_I2645	0.8022721764	7.3781310611	3.42715306700497e-08	acpS	holo-[acyl-carrier-protein] synthase
ABZ98754	LEPBI_I2676	0.6528368148	8.6629562717	1.87995337994497e-07	NA	CarD family transcriptional regulator
ABZ98759	LEPBI_I2681	0.5521296461	7.5907715913	5.79339860956089e-05	ctaF	cytochrome c oxidase polypeptide IVB
ABZ98771	LEPBI_I2693	0.5956358893	6.880796331	3.55004568369791e-06	NA	hypothetical protein
ABZ98804	LEPBI_I2726	-0.3553242607	7.8695769031	0.0005982467	NA	hypothetical protein
ABZ98840	LEPBI_I2762	-0.3423628049	8.1605496225	0.000275716	NA	putative iron-regulated membrane protein
ABZ98851	LEPBI_I2774	-0.666299587	7.3436874777	1.28988609770586e-08	moaA	molybdenum cofactor biosynthesis protein A
ABZ98867	LEPBI_I2790	-0.3912037897	6.9076248179	0.0095535037	NA	putative sulfide quinone reductase
ABZ98878	LEPBI_I2801	0.53113126	5.4032309869	0.004214287	rbpA1	RNA-binding protein
ABZ98893	LEPBI_I2818	-0.4190354231	7.2377712193	0.000101318	NA	hypothetical protein
ABZ98909	LEPBI_I2838	0.5033014613	6.5804017383	0.0001140561	ptsN	PTS system transporter subunit IIA
ABZ98960	LEPBI_I2891	0.3151714782	8.84874063	0.0009630779	icd	isocitrate dehydrogenase
ABZ98975	LEPBI_I2906	0.4053266469	8.8711003382	6.04399516589403e-05	NA	hypothetical protein
ABZ98979	LEPBI_I2910	0.4674824599	8.2095073495	7.05958228158738e-05	NA	putative transporter
ABZ98994	LEPBI_I2925	-0.3371458427	7.3406666628	0.0013230533	msrA1	peptide methionine sulfoxide reductase MsrA
ABZ98996	LEPBI_I2927	3.1758071978	8.8006624451	4.19359519202343e-12	NA	hypothetical protein
ABZ99001	LEPBI_I2932	0.3784295768	8.8679048014	0.0032542728	NA	RNA polymerase ECF-type sigma factor
ABZ99004	LEPBI_I2935	0.3741096515	8.9862083644	0.000219236	NA	hypothetical protein
ABZ99014	LEPBI_I2946	-0.4199914657	8.3321432166	0.0002136427	fliG1	flagellar motor switch protein FliG
ABZ99023	LEPBI_I2955	0.4340186168	7.5301055695	0.0001722823	norC	nitric oxide reductase, cytochrome c-containing subunit
ABZ99054	LEPBI_I2987	0.4854762278	6.1528032782	0.0025553633	NA	hypothetical protein
ABZ99079	LEPBI_I3012	-0.4206209675	7.6128738127	6.32884811962751e-05	NA	putative two-component response regulator
ABZ99080	LEPBI_I3013	0.5371695767	7.7516830312	4.4406489489337e-08	NA	putative signal peptide
ABZ99082	LEPBI_I3016	-0.4055482461	6.3561509552	0.0021607919	NA	putative signal peptide
ABZ99113	LEPBI_I3047	0.4989748564	7.8808125827	0.0009236461	NA	hypothetical protein
ABZ99124	LEPBI_I3058	-0.5752004796	5.0261390985	0.0094173014	NA	hypothetical protein
ABZ99175	LEPBI_I3109	-0.4963198142	6.9561742253	4.95508234108735e-06	NA	putative signal peptide
ABZ99179	LEPBI_I3113	0.4998455268	6.6085493405	0.0039383443	NA	hypothetical protein
ABZ99225	LEPBI_I3160	0.3386308559	8.7179847787	0.0022513701	NA	putative ABC transporter periplasmic phosphate-binding protein
ABZ99232	LEPBI_I3167	-0.6079086977	6.0192485143	3.70803602055551e-05	NA	hypothetical protein
ABZ99248	LEPBI_I3183	-0.570868464	6.3620837308	1.89607476485205e-05	NA	hypothetical protein
ABZ99251	LEPBI_I3186	1.4922270776	8.321380916	8.216587493592e-08	NA	hypothetical protein
ABZ99256	LEPBI_I3191	-0.6719912064	6.0504128859	1.08400974111352e-05	NA	putative ferredoxin-like protein, 2 Fe-2S
ABZ99259	LEPBI_I3194	-0.5087967461	7.0270623118	1.88874931997581e-05	NA	hypothetical protein

ABZ99265	LEPBI_I3200	0.4384880051	8.6398336858	0.0027501385	NA	hypothetical protein
ABZ99275	LEPBI_I3210	-0.413754804	6.3532003318	0.0044177411	csrA	carbon storage regulator
ABZ99283	LEPBI_I3218	-0.4319404252	6.4514488171	0.0020740618	NA	hypothetical protein
ABZ99287	LEPBI_I3222	0.3261627687	7.8164583803	0.002903737	NA	hypothetical protein
ABZ99319	LEPBI_I3254	-0.3742412025	9.1818383859	0.0014091479	NA	putative sugar transport protein
ABZ99343	LEPBI_I3278	-0.4825638828	6.099774894	0.0009376137	NA	putative chemotaxis protein CheC
ABZ99363	LEPBI_I3298	-0.439878993	8.4075640885	0.0004256592	NA	hypothetical protein
ABZ99370	LEPBI_I3305	-0.3223912428	7.5003742532	0.0082206676	NA	putative phosphoribosyl-AMP cyclohydrolase
ABZ99376	LEPBI_I3311	-0.4586314684	7.8524955551	0.0007081493	NA	hypothetical protein
ABZ99389	LEPBI_I3324	0.6058280703	7.7358589513	6.11042585264906e-07	NA	hypothetical protein
ABZ99399	LEPBI_I3335	-0.377028914	8.2726394266	0.0026319204	NA	hypothetical protein
ABZ99409	LEPBI_I3345	-0.4334134953	7.0849435797	0.0003898781	arsR3	transcription regulator ArsR
ABZ99419	LEPBI_I3355	-0.6093463886	6.395006823	2.09864317298447e-06	NA	TetR family transcriptional regulator
ABZ99420	LEPBI_I3356	-0.4877666167	8.493032301	1.30353367302616e-06	NA	putative nitroreductase
ABZ99432	LEPBI_I3368	-0.4483413528	7.1475286481	8.87063727811892e-05	NA	ArsC family arsenate permease
ABZ99455	LEPBI_I3391	-0.3617947419	6.6724550427	0.0096389528	NA	hypothetical protein
ABZ99472	LEPBI_I3410	-0.5333397967	6.1778947223	0.000801927	NA	putative transcription antitermination protein NusG
ABZ99477	LEPBI_I3415	-0.524617456	7.6834640598	2.17282055706122e-05	NA	hypothetical protein
ABZ99480	LEPBI_I3418	-0.4331529441	7.0366007087	0.0017632099	NA	hypothetical protein
ABZ99488	LEPBI_I3427	-0.4299087942	6.9426988771	0.000386522	NA	hypothetical protein
ABZ99497	LEPBI_I3436	-0.277544803	8.1128771637	0.0063167725	NA	putative ferredoxin--NADP reductase
ABZ99507	LEPBI_I3447	-0.3139146569	7.7745925292	0.0036305521	NA	hypothetical protein
ABZ99515	LEPBI_I3455	-0.4215499712	8.5563224672	0.0001181132	NA	putative feruloyl esterase
ABZ99527	LEPBI_I3468	-0.3210827308	8.5530835698	0.0021345265	NA	putative hydrolase
ABZ99533	LEPBI_I3474	-0.3607212055	7.2141008693	0.0033953654	parA	chromosome partitioning protein ParA
ABZ99550	LEPBI_II0012	0.4339769581	7.9400143985	1.08045653502463e-06	batA	von Willebrand factor A
ABZ99560	LEPBI_II0022	0.4413778314	6.7068700112	0.0057554276	NA	phosphotyrosine protein phosphatase
ABZ99568	LEPBI_II0030	0.3892276095	6.6348012584	0.0082860263	NA	cyclic nucleotide binding protein
ABZ99572	LEPBI_II0034	0.5057210684	6.0240678714	0.0008507999	NA	hypothetical protein
ABZ99573	LEPBI_II0035	0.3924107099	8.5963102811	6.37753397994937e-05	NA	hypothetical protein
ABZ99603	LEPBI_II0065	0.3261344766	8.6800882416	0.0090048337	NA	hypothetical protein
ABZ99613	LEPBI_II0075	0.287892826	8.4737579511	0.0046659708	traB	Pheromone shutdown protein, TraB family
ABZ99615	LEPBI_II0077	0.7103382402	5.60107023	0.0003317945	NA	hypothetical protein
ABZ99625	LEPBI_II0088	0.4407570414	7.0127542183	0.0013536718	NA	hypothetical protein
ABZ99637	LEPBI_II0101	0.2926096995	8.1163761914	0.0039383443	NA	hypothetical protein
ABZ99673	LEPBI_II0138	0.3367520482	8.2848587832	0.0034869109	NA	hypothetical protein
ABZ99676	LEPBI_II0141	-0.3570473444	7.4161554389	0.002119746	NA	hypothetical protein
ABZ99681	LEPBI_II0146	0.5588470661	8.5248328123	2.44351198595913e-09	NA	peroxidase
ABZ99698	LEPBI_II0163	0.4212768256	7.6268817081	0.0001042093	NA	DnaK suppressor; DksA/TraR zinc finger signature
ABZ99724	LEPBI_II0191	0.4867766907	7.0065893202	0.0043754295	NA	hypothetical protein
ABZ99748	LEPBI_II0215	0.5807646906	6.8462999763	0.0082206676	NA	hypothetical protein
ABZ99768	LEPBI_II0235	-0.5253194612	5.9250789968	0.000716646	NA	hypothetical protein
ABZ99773	LEPBI_II0240	0.5303330161	5.8480491694	0.0027790524	NA	hypothetical protein
ABZ99815	LEPBI_p0001	0.5820177283	7.3651490081	8.13564386263533e-06	NA	Putative chromosome partitioning protein ParA
ABZ99816	LEPBI_p0002	0.7682749084	7.2179907489	1.50287060275832e-06	NA	Putative chromosome partitioning protein ParB
ABZ99817	LEPBI_p0003	0.6748879695	5.2512951295	0.0014898741	NA	Putative bacteriophage protein
ABZ99820	LEPBI_p0006	0.8432546614	5.5779477497	7.21534581289544e-05	NA	hypothetical protein
ABZ99821	LEPBI_p0007	0.9238064157	5.8743352978	2.41771046723606e-06	NA	Putative transcriptional regulator
ABZ99822	LEPBI_p0008	1.008242835	5.302500316	2.74639316105206e-08	NA	hypothetical protein
ABZ99824	LEPBI_p0010	0.9068922467	7.7665292155	1.98262136460412e-10	NA	Putative phosphoserine phosphatase; putative membrane protein
ABZ99825	LEPBI_p0011	0.6369867187	6.830762051	0.0015339255	NA	Putative signal transduction histidine kinase; putative membrane protein
ABZ99826	LEPBI_p0012	0.8197660629	6.6735418498	2.08240823245777e-07	hemS	Hemin degradation protein HemS
ABZ99827	LEPBI_p0013	1.008185783	7.7027560584	6.12689934885853e-21	hemV	ABC-type hemin transport system, ATPase

ABZ99828	LEPBI_p0014	0.6366044815	6.9039504166	8.1741169666748e-07	hemU	ABC-type hemin transport system, permease; putative membrane protein
ABZ99829	LEPBI_p0015	0.4792258211	7.1398566179	0.0003898781	hemT	ABC-type Fe3+-hydroxamate transport system, periplasmic component
ABZ99830	LEPBI_p0016	0.8101200983	6.2040294925	5.19006213628056e-08	NA	hypothetical protein
ABZ99831	LEPBI_pa0017	0.4905581995	5.8793469236	0.0042089772	NA	hypothetical protein
ABZ99832	LEPBI_p0018	0.73815268	8.0859708578	8.44458317936533e-09	NA	Putative TonB-dependent outer membrane receptor
ABZ99833	LEPBI_p0019	0.6420859387	7.9931573001	6.1619862074208e-06	recD	ATP-dependent exoDNAse (exonuclease V), alpha subunit, helicase superfamily I member
ABZ99834	LEPBI_p0020	0.6346749011	8.5342270881	1.75862071089783e-06	recB	Exodeoxyribonuclease V beta chain, UvrD subfamily
ABZ99835	LEPBI_p0021	0.8989665509	8.6907703717	7.84411672078581e-17	recC	Exodeoxyribonuclease V gamma chain
ABZ99836	LEPBI_p0022	0.7128686385	7.7620920425	1.08045653502463e-06	NA	Putative hydrolase; putative signal peptide
ABZ99837	LEPBI_p0023	0.6242648115	6.8247767908	0.0010813264	NA	Putative transcription regulator; putative membrane protein
ABZ99838	LEPBI_p0024	0.673743941	9.3917321385	5.27524958492304e-09	NA	hypothetical protein
ABZ99839	LEPBI_p0025	0.6656242669	6.1565362514	2.01789277718986e-05	NA	hypothetical protein
ABZ99840	LEPBI_pa0026	0.7196111825	7.5357822201	3.81825923627235e-08	NA	Putative antirestriction protein
ABZ99841	LEPBI_p0027	1.0172998922	7.3274523354	3.04545470130188e-11	NA	Putative transposase
ABZ99843	LEPBI_p0029	0.9297860944	6.7618158201	1.29475564911866e-12	NA	Hypothetical protein; putative signal peptide
ABZ99844	LEPBI_p0030	0.8193615584	7.33941684	6.59519972216601e-07	NA	hypothetical protein
ABZ99846	LEPBI_p0032	0.9058403551	6.3978836137	5.56667529881021e-10	NA	hypothetical protein
ABZ99847	LEPBI_p0033	0.7407386963	9.5098120683	1.3074327911984e-10	NA	Putative protein with DEAD/DEAH box helicase and with type III restriction enzyme motif
ABZ99851	LEPBI_p0039	0.7096282426	6.7888397549	7.12620451991485e-07	NA	hypothetical protein
ABZ99852	LEPBI_p0040	0.9973667608	5.6013263532	1.21735438619597e-08	NA	hypothetical protein
ABZ99853	LEPBI_p0041	0.9075433537	7.3875574789	3.17106296253733e-15	NA	Hypothetical protein; putative signal peptide
ABZ99855	LEPBI_p0043	0.432697513	7.0849774376	0.0011926719	NA	ABC-type transport system, ATP binding protein
ABZ99856	LEPBI_p0044	0.6927673057	7.9837682777	1.23827372587425e-09	NA	ABC-type transport system, permease ; putative membrane protein; putative signal peptide
ABZ99857	LEPBI_p0045	0.7211705802	7.0966919294	6.11042585264906e-07	NA	hypothetical protein
ABZ99858	LEPBI_p0046	0.7491447934	7.0790062422	7.08723025508587e-08	NA	Putative signal transduction histidine kinase; putative membrane protein
ABZ99859	LEPBI_p0047	0.9115608528	6.735039807	4.36766708796601e-09	NA	Putative transcriptional regulator, TetR family
ABZ99860	LEPBI_p0048	0.6278702803	5.4939518706	0.0007394849	NA	Putative dihydrofolate reductase
ABZ99861	LEPBI_p0049	0.7716498047	5.6754022688	2.12125592542439e-07	NA	hypothetical protein
ABZ99862	LEPBI_p0050	0.9243525657	5.1601873764	2.94414575921383e-06	NA	hypothetical protein
ABZ99863	LEPBI_p0051	0.7321871291	6.9466021116	3.61349106467902e-08	NA	Putative rRNA methylase
ABZ99864	LEPBI_p0052	0.7172206055	6.777167293	9.1793097362508e-09	NA	serine/threonine protein kinase
ABZ99865	LEPBI_p0053	0.6372939363	7.1939608202	1.60756847364224e-07	NA	Putative GGDEF/response regulator receiver domain protein
ABZ99866	LEPBI_p0054	0.4399707233	7.4959403628	0.0087002101	NA	Hypothetical protein; putative signal peptide
ABZ99867	LEPBI_p0055	0.6683091158	6.9780245685	8.13564386263533e-06	NA	hypothetical protein
ABZ99868	LEPBI_p0056	0.6007525895	7.5257751057	3.43353500980654e-07	mdoD	glucan biosynthesis protein D
ABZ99870	LEPBI_p0058	0.7413865964	8.1383401895	3.48291710684433e-09	NA	glucosyltransferase MdoH
ABZ99871	LEPBI_p0059	0.686875607	6.867902671	6.75986539572701e-05	NA	Putative hydrolase
ABZ99872	LEPBI_p0060	0.9723424946	6.2775212019	7.23485279350627e-10	NA	hypothetical protein
ABZ99873	LEPBI_p0061	0.4788228999	6.2229614625	0.0045058836	NA	Putative bacteriophage protein

Table S5 (cont.). Differentially expressed genes in B_120vs48.

Gene	Synonym	logFC	logCPM	FDR	Symbol	Product
ABZ96156	LEPBI_I0009	-0.6612900152	7.5171605832	1.66494800968643e-12	NA	putative lipoprotein
ABZ96159	LEPBI_I0012	0.3190409883	8.8520236062	0.0041583301	NA	hypothetical protein
ABZ96173	LEPBI_I0026	0.4119711902	7.4091532672	0.0008802342	NA	putative inositol monophosphatase
ABZ96182	LEPBI_I0035	0.3603269509	9.0393467659	0.0002226065	NA	putative signal peptide
ABZ96186	LEPBI_I0039	0.3566987517	8.1434524694	0.000135919	NA	hypothetical protein
ABZ96191	LEPBI_I0044	0.477932868	7.4691987591	1.85588817454207e-06	NA	putative carboxylesterase 2
ABZ96246	LEPBI_I0099	0.2795594057	8.6933414056	0.0026211731	NA	hypothetical protein
ABZ96249	LEPBI_I0102	0.2541710366	8.8243622577	0.0055241215	NA	putative oxidoreductase
ABZ96254	LEPBI_I0107	0.2802221078	9.4566216282	0.0070738372	NA	long-chain-fatty-acid--CoA ligase
ABZ96257	LEPBI_I0110	0.4127474482	8.2890135629	0.0003965156	NA	hypothetical protein
ABZ96309	LEPBI_I0164	-0.3939633312	8.7103882132	0.0004051183	NA	GDP-mannose 4,6-dehydratase
ABZ96383	LEPBI_I0238	-1.381858042	8.4718942078	3.95480757220242e-10	NA	4-diphosphocytidyl-2-C-methyl-D-erythritol kinase
ABZ96400	LEPBI_I0255	-0.3420110267	7.440287296	0.0023130868	aatA	hypothetical protein
ABZ96412	LEPBI_I0267	-0.8163227986	9.2729552605	5.53908046562187e-09	NA	putative signal peptide
ABZ96419	LEPBI_I0275	-2.1112446	8.7558557537	5.09518921179537e-06	NA	hypothetical protein
ABZ96433	LEPBI_I0289	-2.1107444042	7.5568783639	4.91659912592585e-27	acpP	hypothetical protein
ABZ96457	LEPBI_I0313	-0.7153417186	8.6493150698	1.26819118569222e-12	NA	putative signal peptide
ABZ96461	LEPBI_I0317	-0.2659876069	8.2399381761	0.0032539778	rpsA	hypothetical protein
ABZ96480	LEPBI_I0337	-0.300539212	8.9877394673	0.0012144118	NA	long-chain-fatty-acid--CoA ligase
ABZ96546	LEPBI_I0404	0.407941258	8.561895118	0.0004009599	lntB	hypothetical protein
ABZ96563	LEPBI_I0421	-0.7767407178	6.3083556045	4.47397018173158e-12	NA	hypothetical protein
ABZ96565	LEPBI_I0423	-0.2902624871	7.3499368732	0.0072957525	NA	putative signal peptide
ABZ96578	LEPBI_I0439	-0.7036358664	4.1900467173	0.0055241215	NA	hypothetical protein
ABZ96579	LEPBI_I0440	-0.8639984292	3.8507075085	0.0072957525	NA	hypothetical protein
ABZ96585	LEPBI_I0446	-0.4476949537	8.5627921642	0.0034801671	NA	rod shape-determining protein MreB
ABZ96591	LEPBI_I0452	-0.5812422758	7.6893387356	1.00406627150222e-05	NA	hypothetical protein
ABZ96606	LEPBI_I0468	-0.3676301765	6.7299693195	0.0080004087	NA	hypothetical protein
ABZ96618	LEPBI_I0480	-0.3026497051	8.4191667254	0.0022342354	NA	mannose-1-phosphate guanylyltransferase
ABZ96653	LEPBI_I0515	-0.4725123471	7.635377771	0.0012597478	NA	30S ribosomal protein S20
ABZ96689	LEPBI_I0551	-0.2789781423	8.5705875405	0.0035672895	NA	putative signal peptide
ABZ96736	LEPBI_I0599	-0.3996052795	8.7046497661	0.0047934142	NA	3-hydroxyacyl-CoA dehydrogenase
ABZ96800	LEPBI_I0667	-0.9990387634	6.0998438356	7.15182344702655e-11	nirD	putative signal peptide
ABZ96802	LEPBI_I0669	-0.3621915136	7.5789632707	0.0006229971	NA	heme oxygenase
ABZ96814	LEPBI_I0682	-0.3816561505	6.1965768182	0.0013790713	NA	hypothetical protein
ABZ96815	LEPBI_I0683	-0.6511386438	7.9980262479	1.07918517872556e-11	NA	putative lipoprotein
ABZ96828	LEPBI_I0696	-0.3076354376	8.240027159	0.006065494	NA	DegT/DnrJ/EryC1/StrS family aminotransferase
ABZ96878	LEPBI_I0746	0.3065078374	7.3347739227	0.0043577495	rhlE	hypothetical protein
ABZ96907	LEPBI_I0777	-0.5028723856	7.6419021	0.0001009693	NA	putative triglyceride lipase
ABZ96930	LEPBI_I0802	-0.3553172689	7.8346278004	0.0029045149	NA	ATP synthase F0 subunit C
ABZ96931	LEPBI_I0803	-0.5060014799	7.7456518602	3.87862380326242e-07	NA	F0F1 ATP synthase subunit B
ABZ96935	LEPBI_I0807	-0.2643123177	8.7187025318	0.009002364	NA	F0F1 ATP synthase subunit beta
ABZ96945	LEPBI_Ia0817	-0.81492372	8.9245698738	2.06388602401253e-23	NA	outer membrane protein
ABZ96985	LEPBI_I0858	-0.9148420442	8.5411101175	4.36593605728824e-05	ptsN	hypothetical protein
ABZ96986	LEPBI_I0859	-1.0229265198	8.5895762377	1.59442180476944e-22	NA	hypothetical protein
ABZ96987	LEPBI_I0860	-0.572231438	8.267598476	1.88758026026515e-09	NA	hypothetical protein
ABZ97030	LEPBI_I0905	-0.7044292908	6.8601264464	3.95480757220242e-10	NA	putative anti-sigma factor antagonist
ABZ97041	LEPBI_I0917	-0.4546353173	6.2952693548	0.0002054767	NA	chemotactic two-component response regulator protein CheY
ABZ97056	LEPBI_I0932	0.50003982	7.1269448598	0.0053668624	NA	putative signal peptide
ABZ97070	LEPBI_I0946	-0.3766196655	7.9648828691	0.0002272597	acdA4	RNA polymerase sigma factor WhiG
ABZ97081	LEPBI_I0957	-1.1350787304	9.281950417	4.1297582606442e-07	icd	hypothetical protein

ABZ97143	LEPBI_I1020	-0.7637859378	5.9886276183	3.14039780699028e-07	NA	50S ribosomal protein L28
ABZ97157	LEPBI_I1034	-0.2948313876	7.5893219551	0.0081895828	norB	hypothetical protein
ABZ97159	LEPBI_I1036	-0.6910850683	5.4535860263	3.78415318597904e-06	NA	putative anti-sigma factor antagonist
ABZ97192	LEPBI_I1069	0.3632527061	8.3096426587	2.68505531370092e-05	NA	putative two-component response regulator
ABZ97198	LEPBI_I1075	0.2323856427	8.2093778511	0.0093966188	NA	putative methyl-accepting chemotaxis protein
ABZ97199	LEPBI_I1078	0.3627172537	7.6273280551	0.0030522487	mreC	putative two-component hybrid sensor and regulator
ABZ97201	LEPBI_I1080	0.247722578	8.205252581	0.0097511263	NA	putative adenylate cyclase
ABZ97202	LEPBI_I1081	0.4630687273	7.5242872954	2.48416520604931e-06	NA	hypothetical protein
ABZ97205	LEPBI_I1084	0.2704646351	8.329559309	0.0023971998	slyD	hypothetical protein
ABZ97211	LEPBI_I1090	0.2790199421	8.1277518762	0.0088908745	rbpA3	hypothetical protein
ABZ97250	LEPBI_I1132	-0.473081949	7.175594265	4.1270375677487e-06	NA	electron transfer flavoprotein subunit alpha
ABZ97251	LEPBI_I1133	-0.362561572	7.6392210962	0.0067801338	NA	electron transfer flavoprotein subunit beta
ABZ97259	LEPBI_I1142	-0.4673235429	6.2273039611	0.0040674019	NA	hypothetical protein
ABZ97276	LEPBI_I1160	-0.3944660562	7.0716500746	0.0005240865	NA	hypothetical protein
ABZ97291	LEPBI_I1175	-2.0644022767	9.6792875369	6.71391255490524e-18	NA	hypothetical protein
ABZ97313	LEPBI_I1197	-0.5606148232	6.483242449	2.36059999257151e-05	NA	putative anti-sigma factor antagonist
ABZ97316	LEPBI_I1200	0.3390675546	7.9699373943	0.0015594684	NA	putative signal transduction histidine kinase
ABZ97318	LEPBI_I1204	0.3629484506	6.7506914094	0.0034801671	NA	hypothetical protein
ABZ97319	LEPBI_I1205	0.5389002904	6.4186494239	0.0005185164	NA	putative integrase/recombinase
ABZ97321	LEPBI_I1207	0.4108965652	7.8390446574	0.0001301509	NA	parallel beta-helix repeat-containing electron transport protein
ABZ97340	LEPBI_I1226	0.4429697277	6.3120906877	0.0038505823	dnaX2	hypothetical protein
ABZ97437	LEPBI_I1327	-0.8641313111	6.7889999511	1.31721538280066e-14	NA	hypothetical protein
ABZ97468	LEPBI_I1358	-0.4319020944	7.691889098	0.0006625938	NA	alkyl hydroperoxide reductase subunit C
ABZ97477	LEPBI_I1368	-0.3512059519	7.2820459311	0.0006005628	batD	putative cytochrome c3
ABZ97483	LEPBI_I1374	-0.4282301675	7.1912266909	0.0026440562	NA	putative signal peptide
ABZ97495	LEPBI_I1386	-0.2654015731	8.6625051522	0.0074516589	NA	Poly(A) polymerase
ABZ97497	LEPBI_I1388	-0.4587180377	7.5491950141	1.88224600007802e-05	NA	50S ribosomal protein L13
ABZ97537	LEPBI_I1429	0.2660604197	7.9317130167	0.0074555604	NA	hypothetical protein
ABZ97567	LEPBI_I1460	-0.8267986644	8.4802439142	1.59976781826278e-16	glpD	hypothetical protein
ABZ97630	LEPBI_I1523	-0.7060513478	6.8410351384	1.97619033093919e-05	NA	hypothetical protein
ABZ97632	LEPBI_I1525	-0.2914881124	8.8261726893	0.0069840095	NA	translation initiation factor IF-2
ABZ97635	LEPBI_I1528	-0.4138941075	6.7617326078	0.0096253755	NA	30S ribosomal protein S15
ABZ97636	LEPBI_I1529	-0.3562845827	8.6444776899	3.20472986407974e-05	NA	polynucleotide phosphorylase/polyadenylase
ABZ97654	LEPBI_I1547	0.288024697	8.2218400671	0.0066671972	NA	putative short-chain dehydrogenase/reductase SDR
ABZ97660	LEPBI_I1553	-0.876715188	7.5904339659	8.48666134684224e-11	pyrF	hypothetical protein
ABZ97661	LEPBI_I1554	0.3617925581	7.4941545673	0.0041310297	cfa	CDP-diacylglycerol--glycerol-3-phosphate 3-phosphatidyltransferase
ABZ97668	LEPBI_I1561	-1.5807666823	6.2211385294	1.83900705002891e-09	htrA1	hypothetical protein
ABZ97671	LEPBI_I1564	-0.9307522566	6.2415586079	0.0007370681	NA	30S ribosomal protein S21
ABZ97681	LEPBI_I1574	-0.5330409481	6.3430604353	2.68874737693891e-05	NA	hypothetical protein
ABZ97696	LEPBI_I1589	-1.8846899859	8.4983223575	6.24735399772845e-59	NA	flagellar filament core protein FlaB
ABZ97704	LEPBI_I1597	-0.5583394004	8.110539693	7.23498199019086e-07	NA	putative signal peptide
ABZ97767	LEPBI_I1660	-0.5583526849	5.13305053	0.0032186098	NA	30S ribosomal protein S16
ABZ97791	LEPBI_I1684	0.4171045032	6.7743214829	0.0004485117	NA	hypothetical protein
ABZ97812	LEPBI_I1706	-0.3954946786	7.2063703734	0.0003575689	argB	ATP-dependent Clp protease proteolytic subunit
ABZ97822	LEPBI_I1716	-0.5952828426	7.0132458599	1.69653070521406e-07	NA	putative transcriptional regulator
ABZ97847	LEPBI_I1741	-0.891119317	6.7762049247	7.76268034852977e-16	NA	putative anti-sigma factor antagonist
ABZ97855	LEPBI_I1749	0.4838614487	7.9980804465	0.0002054767	NA	hypothetical protein
ABZ97861	LEPBI_I1755	0.3162558866	7.9819430619	0.0042971334	NA	phosphoribosylaminoimidazole carboxylase ATPase subunit
ABZ97871	LEPBI_I1765	0.2946413587	7.4026839301	0.0043279602	NA	hypothetical protein
ABZ97879	LEPBI_I1773	-0.573901939	6.4431406362	1.21595748665141e-05	NA	50S ribosomal protein L21
ABZ97881	LEPBI_I1775	-0.6972470104	5.5761902923	6.20324896603489e-06	NA	50S ribosomal protein L27
ABZ97891	LEPBI_I1785	0.4136729593	7.6229822779	0.0006925418	NA	putative N-acyl-L-amino acid amidohydrolase, M20D peptidase
ABZ97902	LEPBI_I1796	0.3278697452	7.9049568417	0.0015701672	NA	putative D-alanine--d-alanine ligase

ABZ97903	LEPBI_I1797	0.3270324048	8.0373298555	0.0040913783	NA	L-lysine 2,3-aminomutase
ABZ97911	LEPBI_I1805	0.2881271395	8.2693992148	0.0023452088	NA	hypothetical protein
ABZ97928	LEPBI_I1822	-0.3906039972	7.2725099307	0.0011061497	NA	hypothetical protein
ABZ97977	LEPBI_I1873	-0.3842990433	7.4199539177	0.0006625938	algI	OmpA domain-containing protein
ABZ97979	LEPBI_I1875	0.3099016837	9.0327880324	0.0025076848	NA	putative cyclic-nucleotide-gated cation channel
ABZ97987	LEPBI_I1883	-0.4405957261	9.1583260814	2.17560511310176e-05	NA	Iron(III) dicitrate TonB-dependent receptor
ABZ98041	LEPBI_I1938	-0.3992931823	8.020023751	0.0083942911	NA	50S ribosomal protein L17
ABZ98042	LEPBI_I1939	-0.531472313	7.6291786255	3.25439530546909e-06	NA	DNA-directed RNA polymerase subunit alpha
ABZ98043	LEPBI_I1940	-0.8006152855	6.8695709785	1.85732696100216e-11	NA	30S ribosomal protein S4
ABZ98044	LEPBI_I1941	-0.740159642	6.0284836945	6.64916919170731e-06	NA	30S ribosomal protein S11
ABZ98045	LEPBI_I1942	-0.9014928617	7.2382197288	8.41170782315474e-08	hemV	30S ribosomal protein S13
ABZ98047	LEPBI_I1944	-1.0289471453	7.582434287	5.31317090133357e-09	hemU	adenylate kinase
ABZ98049	LEPBI_I1946	-0.3010378157	7.5663647491	0.0042243594	hemT	50S ribosomal protein L15
ABZ98050	LEPBI_I1947	-0.433194137	6.7507264781	0.0099448054	NA	50S ribosomal protein L30
ABZ98052	LEPBI_I1949	-0.4904788189	6.9013316787	3.20472986407974e-05	NA	50S ribosomal protein L18
ABZ98053	LEPBI_I1950	-0.3694822207	7.9281292974	0.0028968931	recD	50S ribosomal protein L6
ABZ98054	LEPBI_I1951	-0.3892808556	7.8034519721	0.0002238489	recB	30S ribosomal protein S8
ABZ98057	LEPBI_I1954	-0.4963693617	5.9771792268	0.0011157196	recC	50S ribosomal protein L14
ABZ98058	LEPBI_I1955	-0.7702669001	5.6680117723	4.02292462791895e-07	NA	30S ribosomal protein S17
ABZ98059	LEPBI_I1956	-1.0677680143	5.7111419031	4.56455192539989e-06	NA	50S ribosomal protein L29
ABZ98062	LEPBI_I1959	-0.6941966347	5.3569232051	0.0048354439	NA	50S ribosomal protein L22
ABZ98063	LEPBI_I1960	-0.4737420207	6.3076127659	0.0007894997	NA	30S ribosomal protein S19
ABZ98069	LEPBI_I1966	-0.5912790493	9.1924111814	3.84359499034162e-06	NA	elongation factor Tu
ABZ98071	LEPBI_I1968	-0.5845482687	7.8629596368	3.44486726644269e-06	NA	30S ribosomal protein S7
ABZ98072	LEPBI_I1969	-0.5329203238	8.4920131929	5.30900847369325e-09	NA	30S ribosomal protein S12
ABZ98074	LEPBI_I1972	-0.286042781	9.6775020841	0.0074555604	NA	DNA-directed RNA polymerase subunit beta
ABZ98075	LEPBI_I1973	-0.833542611	7.1433181785	2.96433609890579e-07	NA	50S ribosomal protein L7/L12
ABZ98077	LEPBI_I1975	-0.386943875	6.9741037207	0.0055118819	NA	50S ribosomal protein L1
ABZ98078	LEPBI_I1976	-0.6775716527	6.9254905332	1.62843732547982e-09	NA	50S ribosomal protein L11
ABZ98081	LEPBI_I1979	-0.3001874796	6.8982566664	0.0082247414	NA	hypothetical protein
ABZ98158	LEPBI_I2056	-0.3038780622	7.6924002243	0.0047607092	NA	succinyl-CoA synthetase subunit beta
ABZ98159	LEPBI_I2057	-0.2667207582	7.7591965031	0.0080004087	NA	succinyl-CoA synthetase subunit alpha
ABZ98182	LEPBI_I2080	-0.3158247999	7.4812934163	0.0023452088	NA	glyceraldehyde-3-phosphate dehydrogenase
ABZ98200	LEPBI_I2098	-0.4920489317	5.8901718969	0.0015337013	NA	30S ribosomal protein S18
ABZ98215	LEPBI_I2113	0.4269211511	6.8529182262	0.0024048226	NA	putative RNA pseudouridine synthase
ABZ98229	LEPBI_I2127	0.2957066097	7.8895959439	0.0017912994	NA	hypothetical protein
ABZ98234	LEPBI_I2132	-1.112099975	8.0151454016	1.08617546087052e-19	NA	flagellar filament 35 kDa core protein
ABZ98235	LEPBI_I2133	-0.811405758	8.7972458296	2.72611633780737e-17	NA	flagellar filament 35 kDa core protein
ABZ98244	LEPBI_I2142	-0.3722476723	6.4495340853	0.0037510156	NA	SET domain-containing protein
ABZ98245	LEPBI_I2143	-0.6290320482	6.1255715526	1.30837034177873e-06	NA	hypothetical protein
ABZ98248	LEPBI_I2146	-0.7257356624	7.0288711353	6.86357336511931e-06	NA	putative sigma-54 modulation protein
ABZ98278	LEPBI_I2179	-0.4755475355	6.1121609277	0.002451789	NA	hypothetical protein
ABZ98280	LEPBI_I2181	-0.3888848578	5.9478068276	0.0057495325	NA	hypothetical protein
ABZ98291	LEPBI_I2192	-0.4630600309	6.0031216541	0.0012102762	NA	hypothetical protein
ABZ98324	LEPBI_I2227	-0.4731668796	5.3580654432	0.0092371269	NA	hypothetical protein
ABZ98345	LEPBI_I2249	-1.0991822171	4.5203356447	2.29476011586199e-09	NA	putative virulence-associated protein B
ABZ98346	LEPBI_I2250	-0.559997825	4.4728321505	0.0078303165	mdoD	PilT protein
ABZ98395	LEPBI_I2300	-0.5536616423	7.289483981	5.17376449119446e-07	NA	thioredoxin
ABZ98407	LEPBI_I2312	-0.3621983814	7.3505483519	0.001529118	NA	hypothetical protein
ABZ98413	LEPBI_I2318	-0.7995067916	4.7075676831	3.58997523828429e-05	NA	putative two-component response regulator
ABZ98420	LEPBI_I2327	-0.4912650606	8.6892593631	5.63415954513082e-08	NA	putative ATPase
ABZ98421	LEPBI_I2328	-0.7392998106	8.0021304043	2.98439452066314e-11	NA	putative anti-sigma factor antagonist
ABZ98427	LEPBI_I2335	-0.4329052301	7.4555872494	1.97619033093919e-05	flaA1	flagellar filament outer layer protein A

ABZ98428	LEPBI_I2336	-0.5060569234	8.2340581999	3.73300983797586e-06	flaA2	flagellar filament outer layer protein
ABZ98429	LEPBI_I2337	-1.0376088409	6.9587776068	6.71391255490524e-18	0	hypothetical protein
ABZ98443	LEPBI_I2352	-0.3475481909	7.2196025597	0.000921407	0	hypothetical protein
ABZ98474	LEPBI_I2384	-0.3700477177	7.5269025376	0.0011061497	0	hypothetical protein
ABZ98487	LEPBI_I2397	-1.1140663516	4.927678441	2.00246336119162e-10	0	hypothetical protein
ABZ98489	LEPBI_I2399	-0.678438591	5.5448259099	0.0004967178	rpmI	50S ribosomal protein L35
ABZ98490	LEPBI_I2400	-0.5554006046	8.3213669627	0.00112598	infC	translation initiation factor IF-3
ABZ98519	LEPBI_I2430	-0.3742000085	8.0574276377	0.0004832863	0	di-heme cytochrome C peroxidase
ABZ98524	LEPBI_I2435	-0.3557064375	8.0091960566	3.0755378280586e-05	0	putative malate synthase-like protein
ABZ98551	LEPBI_I2462	-0.5384648737	5.6350417864	0.0003155003	0	putative signal peptide
ABZ98603	LEPBI_I2518	-0.4394300898	7.5926052631	0.0002051016	0	hypothetical protein
ABZ98604	LEPBI_I2520	0.3090430846	9.1954357002	0.0026211731	0	putative cAMP-dependent protein kinase
ABZ98605	LEPBI_I2521	0.422194278	8.6166309669	0.000266701	0	putative phopholipase
ABZ98607	LEPBI_I2523	0.5465300829	7.6675402688	5.63415954513082e-08	0	hypothetical protein
ABZ98608	LEPBI_I2524	0.5506121812	6.198086361	0.0002357489	0	hypothetical protein
ABZ98609	LEPBI_I2525	0.4102630093	9.7055523007	3.44486726644269e-06	0	putative adenylate cyclase
ABZ98610	LEPBI_I2526	0.6656262495	7.0942151349	6.80563584498791e-06	0	putative two-component response regulator
ABZ98611	LEPBI_I2527	0.5939038775	7.2658920621	2.88451892694582e-06	0	hypothetical protein
ABZ98613	LEPBI_I2529	0.5331452303	6.8129801391	0.003642449	0	hypothetical protein
ABZ98615	LEPBI_I2531	0.4045546221	6.71072081	0.0023964165	0	hypothetical protein
ABZ98617	LEPBI_I2536	0.3507379508	7.1758585521	0.0025075664	0	putative signal peptide
ABZ98618	LEPBI_I2537	0.4194708407	8.1946182144	6.64826703554731e-06	0	hypothetical protein
ABZ98619	LEPBI_I2538	0.4421412972	7.1152351795	0.0007370681	0	alpha/beta family hydrolase
ABZ98622	LEPBI_I2541	0.5069514821	7.0526330308	0.0002054767	0	putative esterase/lipase
ABZ98624	LEPBI_I2543	0.2686886272	7.9441721937	0.0099448054	aatA	aspartate aminotransferase A
ABZ98625	LEPBI_I2544	0.3263340895	9.2133021353	0.0003360798	0	putative DNA repair helicase
ABZ98642	LEPBI_I2561	0.4870965469	8.6356501772	0.0011131725	0	hypothetical protein
ABZ98666	LEPBI_I2585	-0.9376721758	6.1305232359	0.002451789	acpP	acyl carrier protein
ABZ98673	LEPBI_I2594	-0.4475245075	7.098391653	3.39814306458814e-05	0	hypothetical protein
ABZ98674	LEPBI_I2595	-0.3583499405	8.7488277069	3.03837085924976e-05	rpsA	30S ribosomal protein S1
ABZ98748	LEPBI_I2670	-0.9468607727	8.0733538377	4.20051635336817e-17	0	putative signal peptide
ABZ98752	LEPBI_I2674	-0.5260805198	8.153967187	8.12630720705156e-08	lntB	apolipoprotein N-acyltransferase
ABZ98754	LEPBI_I2676	-1.1212551061	8.6629562717	3.68374385101756e-26	0	CarD family transcriptional regulator
ABZ98772	LEPBI_I2694	-0.4738713343	7.6070395397	2.68874737693891e-05	0	putative cytochrome c
ABZ98776	LEPBI_I2698	0.3649529065	6.7307007754	0.0018198154	0	hypothetical protein
ABZ98783	LEPBI_I2705	-0.6718375013	7.3673715019	6.17298361804489e-10	0	hypothetical protein
ABZ98788	LEPBI_I2710	-0.948169841	4.0922227985	0.0017207284	0	hypothetical protein
ABZ98790	LEPBI_I2712	-1.3983151396	4.3194947743	2.90106713371941e-12	0	hypothetical protein
ABZ98804	LEPBI_I2726	-0.3884349884	7.8695769031	4.46005392214808e-05	0	hypothetical protein
ABZ98815	LEPBI_I2737	0.3861964091	7.5551379856	0.0005456934	0	AraC family transcriptional regulator
ABZ98821	LEPBI_I2743	0.2599206396	9.242422636	0.0087573309	0	hypothetical protein
ABZ98838	LEPBI_I2760	-0.3005250796	9.2277980906	0.0012597478	0	putative TonB-dependent receptor protein
ABZ98839	LEPBI_I2761	-0.4415035116	6.307398388	0.0002447357	0	putative signal peptide
ABZ98844	LEPBI_I2767	0.4425831295	6.4506955641	0.0003871886	nirD	nitrite reductase small subunit
ABZ98882	LEPBI_I2805	0.3622426739	7.216960189	0.0005240865	0	putative transcriptional regulator
ABZ98895	LEPBI_I2820	0.343648487	7.4968734428	0.0027443475	0	hypothetical protein
ABZ98899	LEPBI_I2824	0.4328302123	7.6481155893	1.55214998443609e-06	0	hypothetical protein
ABZ98900	LEPBI_I2825	0.486824352	8.4659944067	5.13607470894924e-08	0	hypothetical protein
ABZ98901	LEPBI_I2827	0.4639412781	8.3177689168	5.5458120398082e-06	rhlE	ATP-dependent RNA helicase RhlE
ABZ98902	LEPBI_I2828	0.7700091748	6.0913107468	1.62707477746209e-08	0	hypothetical protein
ABZ98903	LEPBI_I2830	0.8833101354	5.62810546	1.32304396351803e-08	0	hypothetical protein
ABZ98905	LEPBI_I2834	0.8773318951	7.7202881334	6.86357336511931e-06	0	hypothetical protein
ABZ98907	LEPBI_I2836	0.3330664139	7.8761247775	0.0029779045	0	ABC transporter inner membrane protein

ABZ98908	LEPBI_I2837	0.3280292224	8.2736938297	0.0001922253	0	ABC transporter inner membrane protein
ABZ98909	LEPBI_I2838	0.4007196232	6.5804017383	0.0013639668	ptsN	PTS system transporter subunit IIA
ABZ98912	LEPBI_I2841	-0.9296696705	5.3527854204	2.00881706859064e-10	0	hypothetical protein
ABZ98931	LEPBI_I2862	-0.5264111618	10.0298131004	0.0011131725	0	hypothetical protein
ABZ98933	LEPBI_I2864	-0.8803892261	8.4763324115	2.54698878359593e-18	0	hypothetical protein
ABZ98944	LEPBI_I2875	-0.2465199435	8.2827508129	0.0049948556	0	putative NADH oxidoreductase
ABZ98945	LEPBI_I2876	-0.3840708812	7.5350746458	0.0012102762	0	hypothetical protein
ABZ98949	LEPBI_I2880	-0.3348701367	8.8395303818	0.0001487297	acdA4	acyl-CoA dehydrogenase
ABZ98960	LEPBI_I2891	-0.2590373151	8.84874063	0.0050938015	icd	isocitrate dehydrogenase
ABZ99004	LEPBI_I2935	0.2873998817	8.9862083644	0.0055891675	0	hypothetical protein
ABZ99022	LEPBI_I2954	-0.2746194326	8.4602040512	0.0078056488	norB	nitric-oxide reductase subunit B
ABZ99041	LEPBI_I2973	-0.4078446444	7.3490824183	0.0002447357	0	putative signal peptide
ABZ99045	LEPBI_I2977	-0.3435172833	7.1455181008	0.0087458232	0	putative two-component response regulator
ABZ99052	LEPBI_I2984	-0.2663619836	8.0645981035	0.0078653978	0	hypothetical protein
ABZ99139	LEPBI_I3073	-0.3054235058	8.1359935871	0.0032186098	mreC	rod shape-determining protein MreC
ABZ99146	LEPBI_I3080	-0.3246794231	6.9508866925	0.0090082021	0	putative anti-sigma factor antagonist
ABZ99147	LEPBI_I3081	-0.397720244	8.2552117214	1.1348815648535e-06	0	putative signal peptide
ABZ99192	LEPBI_I3126	-0.607126036	7.5643481771	8.67233037454515e-10	slyD	FKBP-type peptidyl-prolyl cis-trans isomerase SlyD
ABZ99193	LEPBI_I3127	-0.4311290045	7.3058244236	0.0047607092	rbpA3	RNA-binding protein
ABZ99214	LEPBI_I3149	-0.4061465295	8.1465944668	0.0007651594	0	hypothetical protein
ABZ99230	LEPBI_I3165	-0.2970392876	7.6838012865	0.0072367826	0	hypothetical protein
ABZ99251	LEPBI_I3186	-2.8257091342	8.321380916	3.73524982166904e-34	0	hypothetical protein
ABZ99279	LEPBI_I3214	-0.345335809	6.9369289273	0.001529118	0	hypothetical protein
ABZ99300	LEPBI_I3235	0.2780369537	7.7072519512	0.0092031851	0	putative membrane-associated phospholipid phosphatase
ABZ99310	LEPBI_I3245	-0.3654483267	8.3103333975	1.21595748665141e-05	0	putative signal peptide
ABZ99432	LEPBI_I3368	0.4831134596	7.1475286481	2.61309270658375e-05	0	ArsC family arsenate permease
ABZ99491	LEPBI_I3430	-0.3936600027	7.5783107977	0.0013657833	0	hypothetical protein
ABZ99513	LEPBI_I3453	-0.3882225957	6.8140996211	0.0029595045	0	hypothetical protein
ABZ99514	LEPBI_I3454	0.4553747925	6.5957530391	0.0007945307	0	hypothetical protein
ABZ99538	LEPBI_I3479	0.3475720093	7.5343536677	0.008294762	dnaX2	DNA polymerase III subunit gamma/tau
ABZ99544	LEPBI_II0006	0.3385488391	6.9121799072	0.0074201949	0	hypothetical protein
ABZ99546	LEPBI_II0008	0.3075280025	8.846672758	0.0008772245	0	Histidine kinase sensor protein
ABZ99548	LEPBI_II0010	0.2822192678	8.4349104784	0.0080322873	batD	von Willebrand factor A
ABZ99552	LEPBI_II0014	0.3665982587	7.2958062658	0.0005289492	0	hypothetical protein
ABZ99561	LEPBI_II0023	0.3123913486	7.5033779598	0.0030244144	0	hypothetical protein
ABZ99572	LEPBI_II0034	0.8572122815	6.0240678714	5.02034019900044e-11	0	hypothetical protein
ABZ99574	LEPBI_II0036	-0.7143154861	6.6179608391	0.0013790713	0	DNA-binding protein (HU-like protein)
ABZ99579	LEPBI_II0041	0.2534885741	8.5020577941	0.0098080302	glpD	glycerol-3-phosphate oxidase
ABZ99599	LEPBI_II0061	-0.3423908056	6.9737258184	0.002090864	0	cytochrome c oxidase, subunit III
ABZ99608	LEPBI_II0070	0.5849835751	6.0551327974	0.0005240865	0	hypothetical protein
ABZ99623	LEPBI_II0085	0.3381127239	8.7527155181	9.33922136517404e-05	0	hypothetical protein
ABZ99636	LEPBI_II0100	0.2652857445	7.9607930649	0.0066671972	0	hypothetical protein
ABZ99637	LEPBI_II0101	0.3781781732	8.1163761914	2.70825014744589e-05	0	hypothetical protein
ABZ99646	LEPBI_II0110	0.3823251616	8.1725594262	1.452091580803e-05	pyrF	Orotidine-5'-monophosphate decarboxylase
ABZ99649	LEPBI_II0113	0.4673905085	9.1160443759	6.02583434026744e-05	cfa	Cyclopropane-fatty-acyl-phospholipid synthase
ABZ99661	LEPBI_II0125	0.265435642	8.355063544	0.006065494	htrA1	HtrA1; signal peptide
ABZ99671	LEPBI_II0135	0.2913320593	8.0229748715	0.004270222	0	hypothetical protein
ABZ99673	LEPBI_II0138	0.3875547145	8.2848587832	0.0002809429	0	hypothetical protein
ABZ99686	LEPBI_II0151	0.2977413412	7.9867838966	0.0066807725	0	two-component response regulator
ABZ99691	LEPBI_II0156	-0.4314517267	8.6263575031	2.85400250219942e-07	0	signal peptide
ABZ99692	LEPBI_II0157	0.277465917	8.2642200335	0.0066671972	0	4-coumarate--CoA ligase
ABZ99704	LEPBI_II0169	0.4187187979	7.3511983529	0.0010325471	0	N-carbamoyl-D-amino acid hydrolase
ABZ99705	LEPBI_II0170	0.3541672602	7.9432972941	0.002254659	argB	acetylglutamate kinase

ABZ99724	LEPBI_II0191	-0.6985965306	7.0065893202	3.34743440555916e-07	0	hypothetical protein
ABZ99748	LEPBI_II0215	0.6826859526	6.8462999763	0.0007697921	0	hypothetical protein
ABZ99770	LEPBI_II0237	-0.5434593895	7.2700050679	5.54484612674846e-06	0	two-component response regulator
ABZ99771	LEPBI_II0238	-0.4311899927	6.574828207	0.0050571826	0	hypothetical protein
ABZ99783	LEPBI_II0250	0.3958211259	7.247079387	0.0012597478	0	hypothetical protein
ABZ99787	LEPBI_II0254	0.3010441472	8.291837379	0.000936312	0	glycosyl transferase
ABZ99788	LEPBI_II0255	0.3574557854	6.4791615574	0.0067772125	0	hypothetical protein
ABZ99789	LEPBI_II0256	0.2893379889	8.6661173164	0.0081078549	0	hypothetical protein
ABZ99796	LEPBI_II0263	0.3662430599	7.9185953632	0.0002660373	0	hypothetical protein
ABZ99799	LEPBI_II0266	0.2967731643	7.8052176651	0.0040841681	0	hemopexin
ABZ99801	LEPBI_II0268	0.2661158229	8.8391420368	0.0042132025	0	C-terminal processing peptidase
ABZ99804	LEPBI_II0271	-0.651601784	5.7861411887	2.88159787555988e-05	0	hypothetical protein
ABZ99810	LEPBI_II0277	0.3371604467	8.724654322	0.0002639945	algI	alginate O-acetyltransferase
ABZ99815	LEPBI_p0001	0.6393202348	7.3651490081	1.65073994155061e-07	0	Putative chromosome partitioning protein ParA
ABZ99816	LEPBI_p0002	0.6555744657	7.2179907489	2.68505531370092e-05	0	Putative chromosome partitioning protein ParB
ABZ99817	LEPBI_p0003	0.6057118967	5.2512951295	0.0023452088	0	Putative bacteriophage protein
ABZ99821	LEPBI_p0007	0.5914254016	5.8743352978	0.002643714	0	Putative transcriptional regulator
ABZ99824	LEPBI_p0010	0.6721161268	7.7665292155	1.67319172028971e-06	0	Putative phosphoserine phosphatase; putative membrane protein
ABZ99825	LEPBI_p0011	0.6519984788	6.830762051	0.0007136707	0	Putative signal transduction histidine kinase; putative membrane protein
ABZ99827	LEPBI_p0013	0.8012462728	7.7027560584	3.20384070665683e-15	hemV	ABC-type hemin transport system, ATPase
ABZ99828	LEPBI_p0014	0.9157153411	6.9039504166	1.04193191890597e-14	hemU	ABC-type hemin transport system, permease; putative membrane protein
ABZ99829	LEPBI_p0015	0.6768367675	7.1398566179	2.45711399227707e-08	hemT	ABC-type Fe3+-hydroxamate transport system, periplasmic component
ABZ99830	LEPBI_p0016	0.4954096268	6.2040294925	0.0006578796	0	hypothetical protein
ABZ99832	LEPBI_p0018	0.8438482178	8.0859708578	3.29903236798477e-12	0	Putative TonB-dependent outer membrane receptor
ABZ99833	LEPBI_p0019	0.8590605591	7.9931573001	8.48666134684224e-11	recD	ATP-dependent exoDNAse (exonuclease V), alpha subunit, helicase superfamily I member
ABZ99834	LEPBI_p0020	0.8254134955	8.5342270881	4.59627947653547e-11	recB	Exodeoxyribonuclease V beta chain, UvrD subfamily
ABZ99835	LEPBI_p0021	0.8495557389	8.6907703717	3.16726925495518e-16	recC	Exodeoxyribonuclease V gamma chain
ABZ99836	LEPBI_p0022	0.8016679732	7.7620920425	7.80435453662061e-09	0	Putative hydrolase; putative signal peptide
ABZ99837	LEPBI_p0023	0.7343732432	6.8247767908	3.45577468114401e-05	0	Putative transcription regulator; putative membrane protein
ABZ99838	LEPBI_p0024	0.9241619182	9.3917321385	2.72611633780737e-17	0	hypothetical protein
ABZ99839	LEPBI_p0025	1.0306013089	6.1565362514	3.45544501841125e-13	0	hypothetical protein
ABZ99840	LEPBI_pa0026	0.7158786536	7.5357822201	8.25674079927612e-09	0	Putative antirestriction protein
ABZ99841	LEPBI_p0027	0.7541498729	7.3274523354	3.87006883308544e-07	0	Putative transposase
ABZ99843	LEPBI_p0029	0.6508955641	6.7618158201	1.64985187327397e-07	0	Hypothetical protein; putative signal peptide
ABZ99844	LEPBI_p0030	0.748228404	7.33941684	2.88451892694582e-06	0	hypothetical protein
ABZ99846	LEPBI_p0032	0.6336258789	6.3978836137	4.73173466677166e-06	0	hypothetical protein
ABZ99847	LEPBI_p0033	0.8045831481	9.5098120683	3.86238795386737e-13	0	Putative protein with DEAD/DEAH box helicase and with type III restriction enzyme motif
ABZ99850	LEPBI_p0038	0.9284663692	4.7973762941	5.54484612674846e-06	0	hypothetical protein
ABZ99851	LEPBI_p0039	0.8276231982	6.7888397549	4.54042599221947e-10	0	hypothetical protein
ABZ99852	LEPBI_p0040	1.2496819304	5.6013263532	5.20539726425048e-15	0	hypothetical protein
ABZ99853	LEPBI_p0041	0.8495831905	7.3875574789	5.20539726425048e-15	0	Hypothetical protein; putative signal peptide
ABZ99854	LEPBI_p0042	0.8308618216	4.3037886679	0.0001650338	0	hypothetical protein
ABZ99855	LEPBI_p0043	0.6583605814	7.0849774376	2.26886226235785e-08	0	ABC-type transport system, ATP binding protein
ABZ99856	LEPBI_p0044	0.7547689876	7.9837682777	2.16126361132689e-12	0	ABC-type transport system, permease ; putative membrane protein; putative signal peptide
ABZ99857	LEPBI_p0045	0.5925231032	7.0966919294	2.40378945295262e-05	0	hypothetical protein
ABZ99858	LEPBI_p0046	0.6952142633	7.0790062422	1.49416241562169e-07	0	Putative signal transduction histidine kinase; putative membrane protein
ABZ99859	LEPBI_p0047	1.0106920196	6.735039807	3.11310645246936e-12	0	Putative transcriptional regulator, TetR family
ABZ99860	LEPBI_p0048	0.7512394187	5.4939518706	5.77598544571485e-06	0	Putative dihydrofolate reductase
ABZ99861	LEPBI_p0049	0.5049058644	5.6754022688	0.0002660373	0	hypothetical protein
ABZ99862	LEPBI_p0050	0.8808289665	5.1601873764	1.00451010584044e-06	0	hypothetical protein
ABZ99863	LEPBI_p0051	0.781169156	6.9466021116	2.52737044569932e-10	0	Putative rRNA methylase
ABZ99864	LEPBI_p0052	0.7212057001	6.777167293	3.75661765188459e-10	0	serine/threonine protein kinase
ABZ99865	LEPBI_p0053	0.6253708147	7.1939608202	3.76872285960213e-08	0	Putative GGDEF/response regulator receiver domain protein

ABZ99866	LEPBI_p0054	0.5651813728	7.4959403628	0.0002326741	0	Hypothetical protein; putative signal peptide
ABZ99867	LEPBI_p0055	0.6698015847	6.9780245685	2.32943036454988e-06	0	hypothetical protein
ABZ99868	LEPBI_p0056	0.7036444214	7.5257751057	1.36050070203523e-10	mdoD	glucan biosynthesis protein D
ABZ99869	LEPBI_p0057	1.0724535312	3.8213042137	0.0001615641	0	hypothetical protein
ABZ99870	LEPBI_p0058	0.6616252303	8.1383401895	4.66845231716461e-08	0	glucosyltransferase MdoH
ABZ99871	LEPBI_p0059	0.58277244	6.867902671	0.0005935163	0	Putative hydrolase
ABZ99872	LEPBI_p0060	0.6647449688	6.2775212019	9.6952807354787e-06	0	hypothetical protein
ABZ99873	LEPBI_p0061	0.4212749784	6.2229614625	0.0090082021	0	Putative bacteriophage protein

Table S5 (cont.). Differentially expressed genes in P_120vs48.

Gene	Synonym	logFC	logCPM	FDR	Symbol	Product
ABZ96150	LEPBI_I0003	-0.3465848184	8.4323463739	0.0017083801	recF	DNA replication and repair protein RecF
ABZ96174	LEPBI_I0027	-0.328927636	8.4751140323	0.0021605427	sodB	superoxide dismutase
ABZ96175	LEPBI_I0028	-0.405897426	9.0412007891	0.0006915646	trpE	anthranilate synthase component I
ABZ96190	LEPBI_I0043	-0.5180716923	8.4109368398	0.0027623277	NA	aromatic-ring-opening dioxygenase domain-containing protein
ABZ96199	LEPBI_I0052	-0.3014755743	8.1845996565	0.0054567866	NA	enoyl-CoA hydratase
ABZ96204	LEPBI_I0057	-0.2841962476	8.2696206764	0.0099501967	acdA2	acyl-CoA dehydrogenase
ABZ96220	LEPBI_I0073	-0.33273166	8.5328401613	0.0017083801	galK	galactokinase
ABZ96222	LEPBI_I0075	0.4838117222	7.2247282366	7.03565623348734e-05	NA	putative nucleotide-diphospho-sugar transferase
ABZ96226	LEPBI_I0079	-0.3388488722	8.2761492677	0.009867344	NA	putative proline iminopeptidase
ABZ96227	LEPBI_I0080	-0.5915260028	6.7289624721	2.70638432364524e-06	NA	putative ferredoxin
ABZ96229	LEPBI_I0082	-0.3979858663	7.5763771911	0.004580483	NA	putative ribosomal RNA methyltransferase
ABZ96230	LEPBI_I0083	-0.7715823759	9.5173057388	0.0015136719	ispA	geranyltranstransferase
ABZ96231	LEPBI_I0084	-0.3993353984	7.3473820632	0.0011998187	ndk	nucleoside diphosphate kinase
ABZ96248	LEPBI_I0101	-0.5232064547	8.0990336105	1.05940095401221e-05	NA	hypothetical protein
ABZ96257	LEPBI_I0110	0.7426734402	8.2890135629	1.64549552653353e-10	NA	hypothetical protein
ABZ96261	LEPBI_I0114	0.4330838675	8.0396991711	0.0029574608	NA	sodium/bile acid cotransporter family protein
ABZ96266	LEPBI_I0119	-0.4077763597	7.2479034611	0.0059233791	NA	hypothetical protein
ABZ96278	LEPBI_I0132	0.7818789099	5.5513463118	3.89028021179621e-05	NA	hypothetical protein
ABZ96280	LEPBI_I0134	-0.4903331863	8.7476751843	0.004950142	NA	hypothetical protein
ABZ96289	LEPBI_I0144	-1.1170872962	7.8637143239	0.0060119604	NA	hypothetical protein
ABZ96303	LEPBI_I0158	-0.4010732436	7.5725021812	0.0005226767	NA	hypothetical protein
ABZ96336	LEPBI_I0191	0.6563135797	5.706934936	0.0003936262	NA	hypothetical protein
ABZ96356	LEPBI_I0211	0.2973967276	8.0495632299	0.0038799754	NA	hypothetical protein
ABZ96358	LEPBI_I0213	-0.3693877193	6.8977483891	0.0080020033	NA	hypothetical protein
ABZ96368	LEPBI_I0223	-0.3491633956	9.8324048536	0.0037493651	fad	acyl-CoA dehydrogenase
ABZ96380	LEPBI_I0235	-0.4750198512	7.7551394192	0.0002563571	rplY	50S ribosomal protein L25/general stress protein Ctc
ABZ96382	LEPBI_I0237	-0.3904357975	8.2110419304	0.0033416609	glmU	bifunctional N-acetylglucosamine-1-phosphate uridyltransferase / glucosamine-1-phosphate N-acetyltransferase
ABZ96383	LEPBI_I0238	-1.6007089214	8.4718942078	9.42565849965789e-10	ispE	4-diphosphocytidyl-2-C-methyl-D-erythritol kinase
ABZ96387	LEPBI_I0242	0.2850967207	8.8705302779	0.0051311951	NA	putative sodium:solute symporter
ABZ96400	LEPBI_I0255	0.3820221119	7.440287296	0.0021605427	NA	hypothetical protein
ABZ96405	LEPBI_I0260	-0.4046584359	8.2875892689	0.0007650903	NA	hypothetical protein
ABZ96412	LEPBI_I0267	-0.8316034554	9.2729552605	4.95235955882606e-07	NA	putative signal peptide
ABZ96419	LEPBI_I0275	-4.1249145411	8.7558557537	1.72780998473544e-13	NA	hypothetical protein
ABZ96420	LEPBI_I0276	0.4550927367	7.9624156938	0.0001101053	NA	hypothetical protein
ABZ96421	LEPBI_I0277	-0.7556732533	7.6638244092	9.81884869969479e-11	NA	hypothetical protein
ABZ96424	LEPBI_I0280	-0.3189026883	9.1068650497	0.0059247933	NA	hypothetical protein
ABZ96433	LEPBI_I0289	-1.1460322609	7.5568783639	9.14331145628664e-06	NA	hypothetical protein
ABZ96445	LEPBI_I0301	0.6334093689	5.3720011992	0.0005716384	NA	hypothetical protein
ABZ96448	LEPBI_I0304	-0.2687072253	8.1753802807	0.0074250362	NA	carbonic anhydrase/acetyltransferase
ABZ96455	LEPBI_I0311	-0.3425432507	8.9218648551	0.0019079003	NA	putative signal peptide
ABZ96457	LEPBI_I0313	-1.0160558099	8.6493150698	7.05895075139778e-18	NA	putative signal peptide
ABZ96479	LEPBI_I0336	-0.4108872139	6.4029268702	0.0054567866	NA	hypothetical protein
ABZ96481	LEPBI_I0338	-0.4071387681	7.7536892697	0.0004225923	NA	enoyl-CoA hydratase
ABZ96495	LEPBI_I0352	-0.3930895779	8.3652800813	0.0004192106	lpxA	UDP-N-acetylglucosamine acyltransferase
ABZ96511	LEPBI_I0368	0.4186048171	6.6345834706	0.0032435295	NA	hypothetical protein
ABZ96513	LEPBI_I0370	0.5195329249	6.3524406641	0.0030395777	NA	PadR family transcriptional regulator
ABZ96523	LEPBI_I0381	0.8874752257	5.4813042618	1.94408448451755e-06	NA	hypothetical protein
ABZ96541	LEPBI_I0399	-0.5845876581	5.5509963723	0.004326537	NA	hypothetical protein
ABZ96544	LEPBI_I0402	-0.6462942757	4.9885344813	0.0088206088	NA	hypothetical protein
ABZ96546	LEPBI_I0404	-0.4297549607	8.561895118	0.0010326024	NA	hypothetical protein

ABZ96547	LEPBI_I0405	0.357467459	7.2882325629	0.0092811519	NA	hypothetical protein
ABZ96551	LEPBI_I0409	-0.4145616472	9.8869100175	0.0049005304	NA	hypothetical protein
ABZ96570	LEPBI_I0428	-0.3968932701	9.0274590485	0.0035828206	NA	sodium:proton antiporter
ABZ96627	LEPBI_I0489	0.3762348404	8.4841156601	0.0001277015	NA	hypothetical protein
ABZ96634	LEPBI_I0496	-0.5961708542	6.6186222019	0.0040349676	NA	putative anti-sigma-B factor antagonist
ABZ96653	LEPBI_I0515	-0.7051262245	7.635377771	1.68902522700376e-05	rpsT	30S ribosomal protein S20
ABZ96654	LEPBI_I0516	-0.3176085689	9.0566114217	0.004326537	NA	putative phosphomannomutase/phosphoglucomutase
ABZ96659	LEPBI_I0521	0.6301135957	6.1395450172	0.0002758791	NA	anti-sigma factor antagonist
ABZ96661	LEPBI_I0523	0.4674596249	8.9086831264	6.59484277669629e-06	NA	putative serine-type endopeptidase
ABZ96669	LEPBI_I0531	-0.4650674406	7.0787833788	0.0018153566	NA	hypothetical protein
ABZ96682	LEPBI_I0544	0.3693400604	8.1492564856	0.0045130234	NA	hypothetical protein
ABZ96690	LEPBI_I0552	0.5754609544	6.1866651601	0.0048981636	NA	putative phospholipid/glycerol acyltransferase
ABZ96699	LEPBI_I0561	-0.6811860413	9.1667038655	1.25143274107633e-10	NA	hypothetical protein
ABZ96709	LEPBI_I0571	0.3338475393	8.3402370778	0.0019725127	NA	putative signal peptide
ABZ96716	LEPBI_I0578	-0.2961407397	8.9939322747	0.0099607003	NA	putative FAD dehydrogenase
ABZ96722	LEPBI_I0584	-0.3556734202	8.0343663942	0.001006871	NA	putative S-adenosyl-L-methionine-dependent methyltransferase
ABZ96750	LEPBI_I0616	0.6695992465	7.4649727953	0.0067209781	NA	alpha/beta family hydrolase
ABZ96753	LEPBI_I0619	0.4830927591	6.8615026577	0.006613847	NA	hypothetical protein
ABZ96784	LEPBI_I0651	0.4536402742	8.1363724317	5.2716106225958e-06	NA	hypothetical protein
ABZ96815	LEPBI_I0683	-0.6918181067	7.9980262479	4.18489172984644e-09	NA	putative lipoprotein
ABZ96842	LEPBI_I0710	0.3770854141	7.0590613758	0.0037962871	NA	hypothetical protein
ABZ96853	LEPBI_I0721	-0.4576441589	8.1684107458	0.0001959568	NA	putative NAD-dependent deacetylase
ABZ96867	LEPBI_I0735	0.4035889653	6.847472028	0.006826869	NA	hypothetical protein
ABZ96872	LEPBI_I0740	0.7834298714	5.8012476674	1.82762747568574e-05	NA	putative PemI family protein
ABZ96875	LEPBI_I0743	0.514184605	6.6995797438	0.0007167412	NA	putative transcriptional regulator
ABZ96878	LEPBI_I0746	0.4179269995	7.3347739227	0.0003641866	NA	hypothetical protein
ABZ96899	LEPBI_I0768	0.5872757406	8.0289807663	6.10352366039483e-10	NA	hypothetical protein
ABZ96912	LEPBI_I0782	0.5666600892	8.1573744179	1.03353684597116e-09	NA	hypothetical protein
ABZ96919	LEPBI_I0789	-0.3759698878	8.36000227	0.0031547638	NA	hypothetical protein
ABZ96929	LEPBI_I0801	-0.3329384444	8.2062485514	0.0023289581	atpB	F0F1 ATP synthase subunit A
ABZ96930	LEPBI_I0802	-0.5650120267	7.8346278004	2.15546304222098e-05	atpE	ATP synthase F0 subunit C
ABZ96931	LEPBI_I0803	-0.5461266489	7.7456518602	5.2716106225958e-06	atpF	F0F1 ATP synthase subunit B
ABZ96932	LEPBI_I0804	-0.4603518622	7.7343555693	0.0012951741	atpH	F0F1 ATP synthase subunit delta
ABZ96935	LEPBI_I0807	-0.3368128449	8.7187025318	0.0024344904	atpD	F0F1 ATP synthase subunit beta
ABZ96944	LEPBI_I0816	0.7077716529	7.1444819117	1.04728750418896e-07	NA	hypothetical protein
ABZ96951	LEPBI_I0824	0.5331950385	6.5269971361	0.000728408	NA	putative permease
ABZ96962	LEPBI_I0835	-0.5280371932	7.7051666859	4.58464278979579e-05	NA	TetR family transcriptional regulator
ABZ96965	LEPBI_I0838	-0.4356114598	6.4640368087	0.0051308296	NA	hypothetical protein
ABZ96966	LEPBI_I0839	-0.4032395415	7.7460831614	0.0003182494	NA	putative glycosyl transferase
ABZ96976	LEPBI_I0849	0.3776500484	8.5707959044	0.0003056972	NA	ABC transporter ATP binding protein/permease
ABZ96980	LEPBI_I0853	0.308086836	7.721263271	0.0060613005	NA	hypothetical protein
ABZ96983	LEPBI_I0856	0.4626753669	8.2192276718	9.33597969250689e-05	NA	hypothetical protein
ABZ96994	LEPBI_I0869	-0.4075812372	7.7972620919	0.0008838864	NA	hypothetical protein
ABZ96996	LEPBI_I0871	-0.3002181209	8.3998819939	0.0041824537	NA	CRP family transcriptional regulator
ABZ97010	LEPBI_I0885	-0.5847718762	9.3602059476	0.0021605427	NA	hypothetical protein
ABZ97028	LEPBI_I0903	0.3152279546	8.2158354469	0.0014745896	nhaC	Na(+)/H(+) antiporter
ABZ97030	LEPBI_I0905	-0.7377422157	6.8601264464	1.0282501503218e-06	NA	putative anti-sigma factor antagonist
ABZ97034	LEPBI_I0910	0.3325641779	7.7357543236	0.0078719432	NA	hypothetical protein
ABZ97050	LEPBI_I0926	0.3416759333	7.9307787253	0.0022010686	NA	putative phosphoserine phosphatase RsbU
ABZ97055	LEPBI_I0931	0.2950431665	8.5850541652	0.0054795742	NA	hypothetical protein
ABZ97058	LEPBI_I0934	-0.5333537281	5.6268364943	0.0047760708	NA	hypothetical protein
ABZ97062	LEPBI_I0938	0.3399664328	8.0099583467	0.0080721675	NA	putative protein-disulfide reductase
ABZ97075	LEPBI_I0951	-0.6452430388	7.3313405598	1.93796242105673e-05	gmk	guanylate kinase

ABZ97081	LEPBI_I0957	-2.1620668067	9.281950417	2.1470020841257e-17	NA	hypothetical protein
ABZ97086	LEPBI_I0962	0.3800765649	8.4361789715	0.0022147949	NA	hypothetical protein
ABZ97098	LEPBI_I0974	-0.4922121195	7.1308126772	0.0011526081	tatA	Sec-independent protein secretion pathway component TatA/E
ABZ97112	LEPBI_I0988	-0.4766082864	9.280791194	0.0007924677	NA	hypothetical protein
ABZ97128	LEPBI_I1004	-0.3637572018	7.7580367082	0.0037219056	NA	hypothetical protein
ABZ97145	LEPBI_I1022	0.3970933623	7.6863675176	0.0092727873	NA	hypothetical protein
ABZ97165	LEPBI_I1042	0.3087027664	9.1121425664	0.0023289581	NA	putative phosphoserine phosphatase RsbP
ABZ97175	LEPBI_I1052	-0.7669480648	7.2850766584	7.32130342108601e-06	NA	hypothetical protein
ABZ97183	LEPBI_I1060	-0.4731344035	7.1009797609	0.0004076831	NA	hypothetical protein
ABZ97186	LEPBI_I1063	0.2905313499	7.9681567943	0.0071163786	aniA	copper-containing nitrite reductase
ABZ97192	LEPBI_I1069	0.3990147448	8.3096426587	6.53723240992135e-05	NA	putative two-component response regulator
ABZ97199	LEPBI_I1078	0.4574818911	7.6273280551	0.0007709657	NA	putative two-component hybrid sensor and regulator
ABZ97200	LEPBI_I1079	0.5072203954	6.0971547346	0.0018153566	NA	putative two-component response regulator
ABZ97201	LEPBI_I1080	0.3199804716	8.205252581	0.0020618473	NA	putative adenylate cyclase
ABZ97202	LEPBI_I1081	0.3590075031	7.5242872954	0.003542725	NA	hypothetical protein
ABZ97205	LEPBI_I1084	-0.2748669565	8.329559309	0.007553353	NA	hypothetical protein
ABZ97246	LEPBI_I1127	0.4054595067	7.9730459052	0.0001074543	NA	putative competence protein
ABZ97249	LEPBI_I1131	-0.4008582738	7.5284883987	0.0068638911	NA	putative signal peptide
ABZ97250	LEPBI_I1132	-0.4788290221	7.175594265	0.0002378597	etfA	electron transfer flavoprotein subunit alpha
ABZ97251	LEPBI_I1133	-0.6203013669	7.6392210962	1.46770287451628e-05	etfB	electron transfer flavoprotein subunit beta
ABZ97256	LEPBI_I1139	0.3370518426	8.6360545257	0.0015337905	NA	hypothetical protein
ABZ97266	LEPBI_I1150	0.5809741606	7.8572688471	3.65943042617853e-06	NA	putative cAMP binding protein
ABZ97277	LEPBI_I1161	0.4889519945	7.2827981765	5.63716442842716e-05	NA	putative signal peptide
ABZ97287	LEPBI_I1171	-0.3557509164	8.7129464391	0.0012631007	hemA	glutamyl-tRNA reductase
ABZ97290	LEPBI_I1174	0.4851144725	6.4494143414	0.0040349676	NA	hypothetical protein
ABZ97291	LEPBI_I1175	-0.8705754228	9.6792875369	0.0054005054	NA	hypothetical protein
ABZ97299	LEPBI_I1183	-0.2729549951	8.7331599331	0.0075216107	cysD	sulfate adenylyltransferase subunit 2
ABZ97301	LEPBI_I1185	0.3665529412	7.1210106645	0.007946382	cysA	sulfate/thiosulfate ABC transporter ATP-binding protein
ABZ97309	LEPBI_I1193	0.3873401455	7.3902106343	0.0046359276	NA	hypothetical protein
ABZ97319	LEPBI_I1205	0.4720799667	6.4186494239	0.0099501967	NA	putative integrase/recombinase
ABZ97323	LEPBI_I1209	0.5542114114	5.8008616387	0.0008838864	NA	hypothetical protein
ABZ97333	LEPBI_I1219	-0.3572069761	8.3426694355	0.0008588362	NA	putative sensory transduction histidine kinase
ABZ97345	LEPBI_I1231	-0.2704337094	8.571468184	0.0080281612	NA	hypothetical protein
ABZ97353	LEPBI_I1240	-0.5142059784	7.4850084556	0.000131053	NA	putative dihydrofolate reductase
ABZ97355	LEPBI_I1242	0.5305519238	6.4626730223	0.0068534504	NA	hypothetical protein
ABZ97364	LEPBI_I1252	-0.318763004	8.5399821618	0.0014831965	NA	putative signal peptide
ABZ97370	LEPBI_I1258	0.3194115253	8.0800415226	0.0093895845	NA	putative methyl-accepting chemotaxis protein
ABZ97388	LEPBI_I1278	-0.3370701585	8.4014578635	0.0021972565	NA	hypothetical protein
ABZ97396	LEPBI_I1286	0.6649565608	6.810047695	0.000214523	NA	hypothetical protein
ABZ97404	LEPBI_I1294	0.4892476826	5.5804958818	0.0053415299	NA	hypothetical protein
ABZ97431	LEPBI_I1321	-0.4570984378	6.2756291366	0.0074860312	NA	putative anti-sigma factor antagonist
ABZ97437	LEPBI_I1327	-0.5544801912	6.7889999511	0.00038669	NA	hypothetical protein
ABZ97440	LEPBI_I1330	0.4568608155	6.9932534404	0.0037962871	NA	hypothetical protein
ABZ97464	LEPBI_I1354	0.8169125225	5.5186133886	0.0001217471	NA	hypothetical protein
ABZ97472	LEPBI_I1362	0.3188488487	8.6948323718	0.0025535563	NA	putative alkaline phosphatase
ABZ97477	LEPBI_I1368	-0.619797769	7.2820459311	1.04728750418896e-07	NA	putative cytochrome c3
ABZ97480	LEPBI_I1371	-0.3606669048	7.4668511821	0.0052729234	NA	hypothetical protein
ABZ97486	LEPBI_I1377	0.419497494	7.4564660655	0.0058224056	NA	hypothetical protein
ABZ97494	LEPBI_I1385	0.2759501202	8.1974771754	0.0082387023	NA	putative membrane associated metalloendopeptidase
ABZ97496	LEPBI_I1387	0.444662856	7.6144755967	0.0019118157	thiL	thiamine-monophosphate kinase
ABZ97497	LEPBI_I1388	-0.4699434838	7.5491950141	0.0003641866	rplM	50S ribosomal protein L13
ABZ97503	LEPBI_I1394	-0.2866911277	8.0707963468	0.0094337249	lolA	outer membrane lipoprotein carrier protein
ABZ97506	LEPBI_I1397	-0.4313458529	7.2504729341	0.0013619918	NA	CDP-diacylglycerol--glycerol-3-phosphate 3-phosphatidyltransferase

ABZ97511	LEPBI_I1402	0.3382388341	7.5856883833	0.0065867363	secF	preprotein translocase subunit SecF
ABZ97515	LEPBI_I1406	-0.3876398463	8.4976351788	0.0006532676	ribBA	riboflavin biosynthesis protein RibA
ABZ97541	LEPBI_I1433	0.2738943408	8.2793048531	0.0098929222	NA	hypothetical protein
ABZ97546	LEPBI_I1438	0.5062369477	5.8008355338	0.0061640951	NA	putative cAMP-binding protein
ABZ97551	LEPBI_I1443	0.5836237296	5.8348426212	0.0007650903	NA	putative transcriptional regulator
ABZ97553	LEPBI_I1445	-0.5749152242	7.0961037277	8.75388598098079e-06	NA	hypothetical protein
ABZ97567	LEPBI_I1460	-0.5124783123	8.4802439142	7.88930375617459e-05	NA	hypothetical protein
ABZ97570	LEPBI_I1463	0.4383238343	7.86280289	4.11643134269312e-05	NA	putative ribosomal subunit pseudouridine synthase
ABZ97596	LEPBI_I1489	0.6172324745	5.5595576434	0.003486031	NA	ankyrin repeat-containing protein
ABZ97600	LEPBI_I1493	0.5468990303	6.3969064779	0.0010253742	NA	putative lipocalin
ABZ97603	LEPBI_I1496	0.4466042181	6.9865158125	0.0030547946	glcK	glucokinase
ABZ97604	LEPBI_I1497	-0.4621631696	7.4631599078	0.0008351309	NA	ABC transporter ATP-binding protein
ABZ97623	LEPBI_I1516	0.3958701853	7.0204290069	0.0055328253	NA	hypothetical protein
ABZ97630	LEPBI_I1523	-0.6444685622	6.8410351384	0.002294038	NA	hypothetical protein
ABZ97656	LEPBI_I1549	0.4486276545	7.5122760919	0.000120926	NA	two-component response regulator
ABZ97660	LEPBI_I1553	-0.5446194324	7.5904339659	0.0019685283	NA	hypothetical protein
ABZ97661	LEPBI_I1554	0.4902948749	7.4941545673	0.0002378597	pgsA	CDP-diacylglycerol--glycerol-3-phosphate 3-phosphatidyltransferase
ABZ97671	LEPBI_I1564	-1.3451265131	6.2415586079	5.7468233958364e-05	rpsU	30S ribosomal protein S21
ABZ97681	LEPBI_I1574	0.5707306153	6.3430604353	0.0001074543	NA	hypothetical protein
ABZ97694	LEPBI_I1587	0.2784712262	8.5001861303	0.0065867363	NA	putative sensor protein
ABZ97696	LEPBI_I1589	-0.9717123837	8.4983223575	4.34537187255095e-11	flaB	flagellar filament core protein FlaB
ABZ97700	LEPBI_I1593	0.2609946438	8.3438819231	0.0091538749	metY	O-acetylhomoserine sulfhydrylase
ABZ97703	LEPBI_I1596	0.4551487875	7.0980862131	0.0065416958	NA	hypothetical protein
ABZ97704	LEPBI_I1597	-0.5761282289	8.110539693	1.98198142460178e-05	NA	putative signal peptide
ABZ97722	LEPBI_I1615	-0.4177424183	9.0272675576	0.0020252339	guaA	GMP synthase
ABZ97764	LEPBI_I1657	-0.3431143073	7.9988839882	0.0053220268	fmt	methionyl-tRNA formyltransferase
ABZ97767	LEPBI_I1660	-0.6941143348	5.13305053	0.0032001374	rpsP	30S ribosomal protein S16
ABZ97768	LEPBI_I1661	-0.6708884051	7.6380244726	0.0024614061	NA	RNA-binding protein
ABZ97790	LEPBI_I1683	0.4285502391	8.3825894498	9.43947665530121e-05	ftsA2	cell division protein FtsA
ABZ97796	LEPBI_I1689	0.7010205183	5.3304241323	0.0019220475	NA	hypothetical protein
ABZ97806	LEPBI_I1700	0.614113179	6.4361794058	2.15546304222098e-05	NA	hypothetical protein
ABZ97812	LEPBI_I1706	-0.3893918253	7.2063703734	0.0034878297	clpP1	ATP-dependent Clp protease proteolytic subunit
ABZ97830	LEPBI_I1724	-0.4344393178	7.2087294425	0.0016423136	NA	hypothetical protein
ABZ97833	LEPBI_I1727	0.383043048	7.0264102162	0.0044696323	NA	putative signal peptide
ABZ97847	LEPBI_I1741	-0.6096007945	6.7762049247	6.40154794650362e-05	NA	putative anti-sigma factor antagonist
ABZ97865	LEPBI_I1759	0.3193953086	7.4184519401	0.0080281612	mraY	phospho-N-acetylmuramoyl-pentapeptide-transferase
ABZ97870	LEPBI_I1764	-0.6228538715	8.1274781223	3.18446572337306e-08	cheR	chemotaxis protein methyltransferase
ABZ97875	LEPBI_I1769	0.2950183011	8.3331070104	0.0065652539	NA	hypothetical protein
ABZ97879	LEPBI_I1773	-0.5110144404	6.4431406362	0.00313964	rplU	50S ribosomal protein L21
ABZ97880	LEPBI_I1774	-0.5435934982	6.9144571884	0.002294038	NA	putative ribosomal protein
ABZ97885	LEPBI_I1779	-0.7296208477	6.4327019678	1.39521919762484e-06	NA	putative nicotinate-nucleotide adenylyltransferase
ABZ97888	LEPBI_I1782	0.5649394692	5.9732500918	0.0011526081	NA	hypothetical protein
ABZ97921	LEPBI_I1815	0.3052269425	8.308784681	0.0021493551	NA	(dimethylallyl)adenosine tRNA methylthiotransferase
ABZ97924	LEPBI_I1818	-0.274117727	8.4685618137	0.0064317899	NA	putative poly(beta-D-mannuronate) O-acetylase
ABZ97925	LEPBI_I1819	0.5972487133	6.8172144328	2.28980566678375e-05	NA	hypothetical protein
ABZ97927	LEPBI_I1821	-0.6567012229	8.3562674465	4.43669557354016e-06	uppP	undecaprenyl-diphosphatase
ABZ97930	LEPBI_I1824	0.436884963	6.946530594	0.0006648421	panC	pantoate--beta-alanine ligase
ABZ97950	LEPBI_I1844	0.3925921857	8.0886165341	0.0001106337	NA	hypothetical protein
ABZ97951	LEPBI_I1845	0.2685393125	8.0356859749	0.0094337249	leuA	2-isopropylmalate synthase
ABZ97952	LEPBI_I1846	0.482511026	7.3132920096	1.98198142460178e-05	hisC	histidinol-phosphate aminotransferase
ABZ97963	LEPBI_I1859	0.3923973953	7.3813533047	0.0019118157	NA	putative signal peptide
ABZ98018	LEPBI_I1915	0.5100547529	5.5744013333	0.006826869	NA	putative metal-sulfur cluster biosynthetic enzyme
ABZ98040	LEPBI_I1937	0.3378012992	8.073085546	0.0036310449	NA	putative histidine kinase sensor protein

ABZ98042	LEPBI_I1939	-0.4608864546	7.6291786255	0.0013889124	rpoA	DNA-directed RNA polymerase subunit alpha
ABZ98046	LEPBI_I1943	-1.3012004398	7.0649249356	2.82844185079457e-15	infA	translation initiation factor IF-1
ABZ98054	LEPBI_I1951	-0.3721758938	7.8034519721	0.00313964	rpsH	30S ribosomal protein S8
ABZ98069	LEPBI_I1966	-0.5326244362	9.1924111814	0.0004933729	tuf	elongation factor Tu
ABZ98071	LEPBI_I1968	-0.4567180651	7.8629596368	0.0040817191	rpsG	30S ribosomal protein S7
ABZ98072	LEPBI_I1969	-0.4591488149	8.4920131929	4.1487307279546e-05	rpsL	30S ribosomal protein S12
ABZ98080	LEPBI_I1978	-0.6975435551	5.7905125906	0.0011526081	secE	preprotein translocase subunit SecE
ABZ98087	LEPBI_I1985	0.3724359339	7.6745792088	0.0045296023	NA	putative acyltransferase
ABZ98089	LEPBI_I1987	0.3662654341	8.0286143262	0.0005199323	NA	putative signal peptide
ABZ98095	LEPBI_I1993	0.4505489848	8.5142399608	0.0018618739	NA	hypothetical protein
ABZ98096	LEPBI_I1994	0.3835346035	8.2818491349	0.0014831965	NA	putative glycosyltransferase
ABZ98099	LEPBI_I1997	-0.3847666029	7.8181494047	0.0043311562	NA	putative glycosyltransferase
ABZ98103	LEPBI_I2001	0.3882873387	8.1249119278	0.0006166455	NA	putative glycosyltransferase
ABZ98110	LEPBI_I2008	0.4188204849	8.4368947925	0.0017277645	NA	putative poly(beta-D-mannuronate) O-acetylase
ABZ98111	LEPBI_I2009	0.3880188558	7.2907612861	0.0034878297	NA	putative glycosyltransferase
ABZ98124	LEPBI_I2022	0.3694977983	7.145317483	0.0082210961	NA	putative acetyltransferase
ABZ98132	LEPBI_I2030	0.4899280822	6.4331027959	0.0035924796	NA	hypothetical protein
ABZ98145	LEPBI_I2043	-0.2901231507	8.1802768376	0.0054836794	NA	putative acyltransferase
ABZ98172	LEPBI_I2070	-0.5720153375	8.6856363965	8.56350509239383e-08	NA	hypothetical protein
ABZ98183	LEPBI_I2081	0.3200003079	7.4557307052	0.0089433944	pgk	phosphoglycerate kinase
ABZ98202	LEPBI_I2100	-0.4433328591	6.9863550498	0.0082387023	rpsF	30S ribosomal protein S6
ABZ98203	LEPBI_I2101	0.3811159406	7.2525426765	0.00208198	NA	hypothetical protein
ABZ98229	LEPBI_I2127	-0.4439884125	7.8895959439	1.98198142460178e-05	NA	hypothetical protein
ABZ98231	LEPBI_I2129	0.3169588587	7.6087470967	0.0065512108	NA	hypothetical protein
ABZ98234	LEPBI_I2132	-0.456614467	8.0151454016	0.0062275734	flaB3	flagellar filament 35 kDa core protein
ABZ98235	LEPBI_I2133	-0.365326289	8.7972458296	0.0041824537	flaB2	flagellar filament 35 kDa core protein
ABZ98239	LEPBI_I2137	0.4101756206	7.1096209818	0.0011327165	NA	hypothetical protein
ABZ98248	LEPBI_I2146	-0.7088020841	7.0288711353	0.000374096	NA	putative sigma-54 modulation protein
ABZ98249	LEPBI_I2147	0.5505289897	5.8837548873	0.0085907083	NA	hypothetical protein
ABZ98266	LEPBI_I2165	0.9116768543	3.6392790013	0.0094090969	NA	hypothetical protein
ABZ98268	LEPBI_I2168	-0.6301276289	6.0291875888	0.0005935716	NA	hypothetical protein
ABZ98271	LEPBI_I2172	0.4562057128	6.7394356694	0.0082387023	NA	hypothetical protein
ABZ98275	LEPBI_I2176	0.4615236662	6.6494428545	0.0022432391	NA	hypothetical protein
ABZ98278	LEPBI_I2179	0.5992845125	6.1121609277	0.0007924677	NA	hypothetical protein
ABZ98285	LEPBI_I2186	0.4410375691	6.8836942332	0.0011526081	NA	hypothetical protein
ABZ98291	LEPBI_I2192	0.57123878	6.0031216541	0.000943771	NA	hypothetical protein
ABZ98293	LEPBI_I2194	0.7200567332	5.1120133647	0.0037493651	NA	hypothetical protein
ABZ98296	LEPBI_I2197	0.8191865784	4.9869206723	0.0001904381	NA	hypothetical protein
ABZ98304	LEPBI_I2205	0.4330638072	6.4664330422	0.0094790597	NA	hypothetical protein
ABZ98312	LEPBI_I2214	0.4423273795	7.6632001577	0.0019404214	NA	hypothetical protein
ABZ98318	LEPBI_I2220	0.4025742308	6.5452615378	0.0077156595	NA	hypothetical protein
ABZ98331	LEPBI_I2234	0.6903418577	4.9382457877	0.0059233791	NA	hypothetical protein
ABZ98335	LEPBI_I2239	0.7102533784	5.2054237171	0.0020618473	NA	hypothetical protein
ABZ98341	LEPBI_I2245	0.6392857686	6.05871683	0.0004849397	NA	hypothetical protein
ABZ98352	LEPBI_I2256	0.7906239141	5.3911615877	5.93555956240899e-05	NA	putative plasmid maintenance system antidote-like protein
ABZ98362	LEPBI_I2266	-0.3438949185	7.4805704326	0.0085907083	NA	hypothetical protein
ABZ98375	LEPBI_I2280	-0.4900438177	7.1885242585	0.0011526081	NA	hypothetical protein
ABZ98376	LEPBI_I2281	0.4434832444	7.0271391164	0.0007155401	NA	glycosyl transferase family protein
ABZ98395	LEPBI_I2300	-0.5243272498	7.289483981	0.0001890673	trxA2	thioredoxin
ABZ98401	LEPBI_I2306	0.3393290669	8.3246879529	0.0017750874	NA	putative signal peptide
ABZ98402	LEPBI_I2307	0.3060571767	7.9480891015	0.0080020033	NA	hypothetical protein
ABZ98427	LEPBI_I2335	-0.3841106363	7.4555872494	0.0028310494	flaA1	flagellar filament outer layer protein A
ABZ98453	LEPBI_I2362	-0.2893281292	8.7556402926	0.0032696034	ilvB2	acetolactate synthase large subunit

ABZ98462	LEPBI_I2371	2.174346113	4.6391423933	5.1573692738627e-18	NA	hypothetical protein
ABZ98471	LEPBI_I2381	0.4274560033	7.0593995773	0.0058673116	NA	hypothetical protein
ABZ98489	LEPBI_I2399	-0.7676223157	5.5448259099	0.0017123876	rpmI	50S ribosomal protein L35
ABZ98490	LEPBI_I2400	-0.59465606	8.3213669627	0.0022432391	infC	translation initiation factor IF-3
ABZ98517	LEPBI_I2428	-0.3973430014	7.3187197921	0.0016927379	pyrE	orotate phosphoribosyltransferase
ABZ98522	LEPBI_I2433	0.5476873842	6.3470128417	0.0062752397	NA	putative globin
ABZ98606	LEPBI_I2522	0.3147371557	8.5283840581	0.0069094998	NA	hypothetical protein
ABZ98607	LEPBI_I2523	0.3345433012	7.6675402688	0.0078229655	NA	hypothetical protein
ABZ98611	LEPBI_I2527	0.5253695297	7.2658920621	0.0005723503	NA	hypothetical protein
ABZ98613	LEPBI_I2529	0.8647976746	6.8129801391	8.95390732328848e-06	NA	hypothetical protein
ABZ98614	LEPBI_I2530	0.6114873347	6.5164962668	0.0020182097	NA	hypothetical protein
ABZ98615	LEPBI_I2531	0.5785644997	6.71072081	0.0001218824	NA	hypothetical protein
ABZ98618	LEPBI_I2537	0.3264343006	8.1946182144	0.0040798439	NA	hypothetical protein
ABZ98677	LEPBI_I2598	0.375564513	7.5386196949	0.0041824537	aroA	3-phosphoshikimate 1-carboxyvinyltransferase
ABZ98680	LEPBI_I2601	-0.4017408618	7.5127624705	0.0010865491	NA	hypothetical protein
ABZ98682	LEPBI_I2603	0.7252847806	7.5041439136	0.007946382	NA	hypothetical protein
ABZ98689	LEPBI_I2610	-0.3697891855	8.9900237414	0.0001959568	NA	membrane-associated Zn-dependent metalloprotease
ABZ98692	LEPBI_I2613	0.4821344626	5.7952043528	0.0073197637	uppS	undecaprenyl pyrophosphate synthase
ABZ98704	LEPBI_I2624	-0.3844204861	7.3014459958	0.0078916475	NA	putative AraC-type transcriptional regulator
ABZ98708	LEPBI_I2629	-0.5955289799	7.6663611145	1.98198142460178e-05	NA	rhodanese-like domain-containing protein
ABZ98709	LEPBI_I2630	0.3502133521	8.00753974	0.0024746209	NA	putative hydroxyacylglutathione hydrolase
ABZ98710	LEPBI_I2631	-0.3215486457	8.0910440011	0.0091365632	NA	hypothetical protein
ABZ98712	LEPBI_I2633	-0.5393487251	8.0872755338	2.48694537893918e-05	msrA2	peptide methionine sulfoxide reductase MsrA
ABZ98724	LEPBI_I2645	-0.845163584	7.3781310611	8.15776676589459e-08	acpS	holo-[acyl-carrier-protein] synthase
ABZ98727	LEPBI_I2648	0.3154686276	8.0094377462	0.0078225683	NA	hypothetical protein
ABZ98739	LEPBI_I2661	-0.3804542882	7.3905641187	0.0036599185	cheX	chemotaxis protein
ABZ98749	LEPBI_I2671	0.326460478	8.1451950262	0.0014060257	NA	hypothetical protein
ABZ98754	LEPBI_I2676	-0.9094310533	8.6629562717	3.49427794291579e-12	NA	CarD family transcriptional regulator
ABZ98759	LEPBI_I2681	-0.532529574	7.5907715913	0.0004566523	ctaF	cytochrome c oxidase polypeptide IVB
ABZ98771	LEPBI_I2693	-0.6677936756	6.880796331	1.47885344619054e-06	NA	hypothetical protein
ABZ98774	LEPBI_I2696	0.7587049493	5.2717665703	0.0007555075	NA	hypothetical protein
ABZ98794	LEPBI_I2716	0.383646399	7.7450315557	0.0040349676	NA	putative iron-sulfur cluster-binding protein
ABZ98831	LEPBI_I2753	0.5379980954	6.304908465	0.0023088549	NA	hypothetical protein
ABZ98836	LEPBI_I2758	0.5001740379	6.2094193211	0.0016303694	NA	hypothetical protein
ABZ98840	LEPBI_I2762	0.4144753481	8.1605496225	5.0770304536292e-05	NA	putative iron-regulated membrane protein
ABZ98851	LEPBI_I2774	0.7351919467	7.3436874777	1.97335246900228e-08	moaA	molybdenum cofactor biosynthesis protein A
ABZ98860	LEPBI_I2783	-0.4007530997	7.185974713	0.0088241096	NA	hypothetical protein
ABZ98891	LEPBI_I2816	0.4424405276	7.0312547752	0.0037219056	NA	hypothetical protein
ABZ98893	LEPBI_I2818	0.6089885817	7.2377712193	1.4857128630473e-07	NA	hypothetical protein
ABZ98895	LEPBI_I2820	0.4666128719	7.4968734428	0.0002257826	NA	hypothetical protein
ABZ98900	LEPBI_I2825	0.3713572877	8.4659944067	0.000613198	NA	hypothetical protein
ABZ98902	LEPBI_I2828	0.5418725897	6.0913107468	0.0027586343	NA	hypothetical protein
ABZ98905	LEPBI_I2834	0.8464552546	7.7202881334	0.000131053	NA	hypothetical protein
ABZ98906	LEPBI_I2835	0.4672998216	7.6840750155	0.0008838864	NA	putative signal peptide
ABZ98921	LEPBI_I2851	0.3560500078	8.2449518225	0.0016558563	NA	putative signal peptide
ABZ98958	LEPBI_I2889	-0.3925537003	7.8212934196	0.0018230637	NA	glycosyl transferase family protein
ABZ98960	LEPBI_I2891	-0.5065120013	8.84874063	1.28014190713094e-07	icd	isocitrate dehydrogenase
ABZ98973	LEPBI_I2904	-0.3915298635	7.4253716062	0.0008838864	tpx	thiol peroxidase
ABZ98975	LEPBI_I2906	-0.3658912828	8.8711003382	0.0012536338	NA	hypothetical protein
ABZ98979	LEPBI_I2910	-0.5002691015	8.2095073495	9.02074381055783e-05	NA	putative transporter
ABZ98994	LEPBI_I2925	0.466330237	7.3406666628	2.93436725945587e-05	msrA1	peptide methionine sulfoxide reductase MsrA
ABZ98996	LEPBI_I2927	-1.5424327656	8.8006624451	0.0020302318	NA	hypothetical protein
ABZ99001	LEPBI_I2932	-0.4624684794	8.8679048014	0.000613198	NA	RNA polymerase ECF-type sigma factor

ABZ99004	LEPBI_I2935	-0.444713747	8.9862083644	3.89028021179621e-05	NA	hypothetical protein
ABZ99014	LEPBI_I2946	0.4365651833	8.3321432166	0.0005675909	fliG1	flagellar motor switch protein FliG
ABZ99023	LEPBI_I2955	-0.3588962583	7.5301055695	0.0060119604	norC	nitric oxide reductase, cytochrome c-containing subunit
ABZ99033	LEPBI_I2965	0.3859991941	7.8359906821	0.00038669	NA	hypothetical protein
ABZ99036	LEPBI_I2968	-0.2918404045	8.8881838009	0.0063273264	metK	S-adenosylmethionine synthetase
ABZ99042	LEPBI_I2974	0.3439846223	7.8599894352	0.0075791385	NA	hypothetical protein
ABZ99080	LEPBI_I3013	-0.5009780884	7.7516830312	5.39648413695307e-06	NA	putative signal peptide
ABZ99082	LEPBI_I3016	0.497562669	6.3561509552	0.0004933729	NA	putative signal peptide
ABZ99097	LEPBI_I3031	0.3275935769	8.4673063242	0.0085907083	NA	hypothetical protein
ABZ99102	LEPBI_I3036	-0.581771985	7.6173156127	0.0001074543	NA	TetR family transcriptional regulator
ABZ99108	LEPBI_I3042	0.4793688088	5.8050937243	0.0078719432	NA	hypothetical protein
ABZ99113	LEPBI_I3047	-0.6386250392	7.8808125827	4.59167732818632e-05	NA	hypothetical protein
ABZ99132	LEPBI_I3066	0.502904017	6.0060446784	0.0041375506	chpK	ChpK toxin protein
ABZ99146	LEPBI_I3080	-0.4528137475	6.9508866925	0.0014356365	NA	putative anti-sigma factor antagonist
ABZ99147	LEPBI_I3081	-0.3929834669	8.2552117214	7.13577246186355e-05	NA	putative signal peptide
ABZ99175	LEPBI_I3109	0.3341191274	6.9561742253	0.0083520736	NA	putative signal peptide
ABZ99179	LEPBI_I3113	-0.6534305388	6.6085493405	0.0002312015	NA	hypothetical protein
ABZ99180	LEPBI_I3114	0.4441292348	6.3476586234	0.0034878297	NA	hypothetical protein
ABZ99183	LEPBI_I3117	-0.3601071878	8.5484961843	0.0030916352	NA	alcohol dehydrogenase
ABZ99189	LEPBI_I3123	-0.4710992041	8.5388436413	5.2716106225958e-06	NA	hypothetical protein
ABZ99191	LEPBI_I3125	-0.307103346	8.8070083216	0.0078348244	NA	putative sodium-dependent phosphate transport protein
ABZ99192	LEPBI_I3126	-0.5017006556	7.5643481771	6.81772814359299e-05	slyD	FKBP-type peptidyl-prolyl cis-trans isomerase SlyD
ABZ99205	LEPBI_I3140	-0.3095264325	7.8256161989	0.0040769112	NA	putative transporter
ABZ99208	LEPBI_I3143	0.5164718712	5.9707872587	0.0044696323	NA	hypothetical protein
ABZ99212	LEPBI_I3147	-0.4600597689	7.2745539692	0.00313964	NA	TetR family transcriptional regulator
ABZ99213	LEPBI_I3148	-0.386798688	8.6843794526	0.0037962871	NA	hypothetical protein
ABZ99214	LEPBI_I3149	-0.3945163535	8.1465944668	0.0053415299	NA	hypothetical protein
ABZ99220	LEPBI_I3155	-0.3077638512	7.7653940295	0.0076199548	NA	putative regulatory protein
ABZ99221	LEPBI_I3156	-0.4647238141	6.4033432053	0.0027988189	NA	putative phosphatase
ABZ99225	LEPBI_I3160	-0.6000834864	8.7179847787	3.95956026315559e-08	NA	putative ABC transporter periplasmic phosphate-binding protein
ABZ99232	LEPBI_I3167	0.7920196408	6.0192485143	1.14639148924868e-06	NA	hypothetical protein
ABZ99242	LEPBI_I3177	-0.3276076597	8.2159367596	0.0078348244	NA	putative zinc-dependent hydrolase
ABZ99245	LEPBI_I3180	-0.6595467293	7.3641271994	0.003837256	NA	hypothetical protein
ABZ99251	LEPBI_I3186	-2.3905096474	8.321380916	5.33627932066809e-17	NA	hypothetical protein
ABZ99256	LEPBI_I3191	0.4831846207	6.0504128859	0.0062752397	NA	putative ferredoxin-like protein, 2 Fe-2S
ABZ99265	LEPBI_I3200	-0.6423205611	8.6398336858	1.67372726416162e-05	NA	hypothetical protein
ABZ99267	LEPBI_I3202	-0.5731242529	9.4600754297	0.0004192106	NA	putative serine protease
ABZ99275	LEPBI_I3210	0.6619599992	6.3532003318	1.47169150596246e-05	csrA	carbon storage regulator
ABZ99283	LEPBI_I3218	0.5053279091	6.4514488171	0.0008838864	NA	hypothetical protein
ABZ99287	LEPBI_I3222	-0.4453244901	7.8164583803	7.03565623348734e-05	NA	hypothetical protein
ABZ99304	LEPBI_I3239	-0.4315182461	8.9123119656	0.0002119257	sulI	dihydropteroate synthase
ABZ99309	LEPBI_I3244	-0.2614684804	8.297660639	0.0079745287	NA	putative chemotaxis sensory transducer
ABZ99317	LEPBI_I3252	-0.3830601933	7.6785092392	0.0022432391	NA	hypothetical protein
ABZ99320	LEPBI_I3255	-0.5367322078	8.0469198411	1.46770287451628e-05	NA	SDR family dehydrogenase/reductase
ABZ99356	LEPBI_I3291	-0.4066203736	8.4689379787	0.0006961692	NA	putative signal peptide
ABZ99370	LEPBI_I3305	0.3757418823	7.5003742532	0.0037552689	NA	putative phosphoribosyl-AMP cyclohydrolase
ABZ99389	LEPBI_I3324	-0.4943854761	7.7358589513	0.0003715785	NA	hypothetical protein
ABZ99393	LEPBI_I3328	-0.3256882509	8.1667560363	0.0084425627	NA	reductase
ABZ99418	LEPBI_I3354	-0.3071143335	9.6133302368	0.0054761668	NA	putative porin
ABZ99419	LEPBI_I3355	0.6156082165	6.395006823	2.21737265420913e-05	NA	TetR family transcriptional regulator
ABZ99427	LEPBI_I3363	-0.3674767713	7.5181215828	0.0010368343	NA	hypothetical protein
ABZ99432	LEPBI_I3368	0.5462408087	7.1475286481	1.47169150596246e-05	NA	ArsC family arsenate permease
ABZ99439	LEPBI_I3375	0.5072570958	6.6522904779	0.0033287725	NA	hypothetical protein

ABZ99474	LEPBI_I3412	-0.524678622	7.8221326514	7.88930375617459e-05	aroD	3-dehydroquinate dehydratase
ABZ99477	LEPBI_I3415	0.4272199157	7.6834640598	0.0027955313	NA	hypothetical protein
ABZ99488	LEPBI_I3427	0.3653940156	6.9426988771	0.0074860312	NA	hypothetical protein
ABZ99494	LEPBI_I3433	-0.3083755555	8.2885852945	0.0094090969	NA	putative signal peptide
ABZ99498	LEPBI_I3437	-0.5906024682	6.0357831416	0.0029018755	NA	putative transcriptional coactivator
ABZ99511	LEPBI_I3451	-0.497805918	7.454628202	4.1487307279546e-05	folE	GTP cyclohydrolase I
ABZ99517	LEPBI_I3457	0.4971530949	6.1786598488	0.0030271736	NA	hypothetical protein
ABZ99522	LEPBI_I3463	-0.4023969219	7.7134058701	0.0015221775	recR	recombination protein RecR
ABZ99544	LEPBI_II0006	0.4030810285	6.9121799072	0.0041824537	NA	hypothetical protein
ABZ99550	LEPBI_II0012	-0.5256615627	7.9400143985	3.18446572337306e-08	batA	von Willebrand factor A
ABZ99560	LEPBI_II0022	-0.495454881	6.7068700112	0.0031679065	NA	phosphotyrosine protein phosphatase
ABZ99568	LEPBI_II0030	-0.6126220562	6.6348012584	2.28675073165793e-05	NA	cyclic nucleotide binding protein
ABZ99573	LEPBI_II0035	-0.5822124063	8.5963102811	8.65174721543477e-09	NA	hypothetical protein
ABZ99582	LEPBI_II0044	-0.2860242403	9.40464043	0.0093508287	mcm2	methylmalonyl-CoA mutase
ABZ99593	LEPBI_II0055	-0.5322496205	6.6825737446	0.0012737515	NA	hypothetical protein
ABZ99599	LEPBI_II0061	-0.4681007719	6.9737258184	0.0001890673	NA	cytochrome c oxidase, subunit III
ABZ99601	LEPBI_II0063	-0.3301895973	7.7706318876	0.0059233791	NA	cbb3-type cytochrome c oxidase subunit II
ABZ99602	LEPBI_II0064	-0.3472995214	8.2613831628	0.0001627265	NA	cbb3-type cytochrome c oxidase subunit I
ABZ99603	LEPBI_II0065	-0.6067055591	8.6800882416	5.67884977075303e-07	NA	hypothetical protein
ABZ99604	LEPBI_II0066	-0.3975334984	8.1115591169	2.63818314094038e-05	gltD	glutamate synthase (NADPH)
ABZ99613	LEPBI_II0075	-0.4618579942	8.4737579511	5.9138814328589e-06	traB	Pheromone shutdown protein, TraB family
ABZ99615	LEPBI_II0077	-0.6236831867	5.60107023	0.0047357694	NA	hypothetical protein
ABZ99622	LEPBI_II0084	-0.5239334984	8.7802207034	0.0004289153	NA	MotA/TolQ/ExbB proton channel protein
ABZ99623	LEPBI_II0085	-0.3902295978	8.7527155181	5.0770304536292e-05	NA	hypothetical protein
ABZ99624	LEPBI_II0087	-0.3282668823	10.0395416751	0.0059233791	NA	acriflavine resistance protein D; transmembrane protein
ABZ99625	LEPBI_II0088	-0.6596894707	7.0127542183	2.09345418977814e-06	NA	hypothetical protein
ABZ99633	LEPBI_II0097	-0.3998600261	7.4584614146	0.0036323262	NA	hypothetical protein
ABZ99637	LEPBI_II0101	-0.4586244252	8.1163761914	6.63980426755572e-06	NA	hypothetical protein
ABZ99645	LEPBI_II0109	-0.3182386372	8.2869700547	0.0014242442	NA	hypothetical protein
ABZ99647	LEPBI_II0111	-0.3921339071	8.3859053142	0.0001904381	NA	dehydrogenase
ABZ99651	LEPBI_II0115	-0.6393611981	6.7043406027	0.0020704346	NA	hypothetical protein
ABZ99673	LEPBI_II0138	-0.4953906574	8.2848587832	2.21737265420913e-05	NA	hypothetical protein
ABZ99679	LEPBI_II0144	-0.2731431162	8.5706823292	0.0091809359	NA	soluble pyridine nucleotide transhydrogenase (NAD(P)(+) transhydrogenase [B-specific])
ABZ99681	LEPBI_II0146	-0.7933640561	8.5248328123	2.07212907270661e-16	NA	peroxidase
ABZ99683	LEPBI_II0148	-0.451824148	7.7275686846	0.0002306847	NA	hypothetical protein
ABZ99684	LEPBI_II0149	-0.5187824377	7.8067718605	5.9138814328589e-06	NA	hypothetical protein
ABZ99691	LEPBI_II0156	-0.4787982371	8.6263575031	1.03980411703154e-06	NA	signal peptide
ABZ99693	LEPBI_II0158	-0.5407955954	6.766522291	0.0001901054	NA	photoactive yellow protein (PYP)
ABZ99695	LEPBI_II0160	-0.4037420177	8.2308799345	2.37232924453697e-05	NA	hypothetical protein
ABZ99698	LEPBI_II0163	-0.6982400571	7.6268817081	1.3875131698766e-10	NA	DnaK suppressor; DksA/TraR zinc finger signature
ABZ99703	LEPBI_II0168	-0.3811547346	7.2301877943	0.0020618473	NA	hypothetical protein
ABZ99706	LEPBI_II0171	-0.3957840424	9.4449306993	0.0006597777	NA	Sigma factor SigB regulation protein RsbU; phosphoserine phosphatase
ABZ99709	LEPBI_II0174	-0.4194746953	7.8848817763	7.88930375617459e-05	NA	hypothetical protein
ABZ99718	LEPBI_II0185	-0.3793429208	7.8476321753	0.0029574608	ahcY	S-adenosyl-L-homocysteine hydrolase
ABZ99722	LEPBI_II0189	-0.3366382023	7.7926973938	0.0048733419	NA	drug/metabolite exporter
ABZ99724	LEPBI_II0191	-1.0302617324	7.0065893202	1.25118517101645e-10	NA	hypothetical protein
ABZ99731	LEPBI_II0198	-0.4083910915	8.1119952872	2.44820576721478e-05	NA	short-chain dehydrogenase/reductase SDR
ABZ99734	LEPBI_II0201	-0.4092870163	6.9973324342	0.0019685283	efp	elongation factor P
ABZ99742	LEPBI_II0209	-0.3715004534	8.2018907443	0.001006871	NA	cyclopropane-fatty-acyl-phospholipid synthase
ABZ99748	LEPBI_II0215	-1.1453543089	6.8462999763	3.95956026315559e-08	NA	hypothetical protein
ABZ99755	LEPBI_II0222	-0.6783968681	5.0883275166	0.0018960495	NA	hypothetical protein
ABZ99773	LEPBI_II0240	-1.0185975536	5.8480491694	5.16472789388073e-10	NA	hypothetical protein
ABZ99781	LEPBI_II0248	-0.3825806953	8.498409416	0.0013619918	NA	hypothetical protein

ABZ99795	LEPBI_II0262	-0.4105201475	9.1438382331	2.37232924453697e-05	NA	hypothetical protein
ABZ99806	LEPBI_II0273	-0.403302027	8.6301660693	0.0004257111	pykF	pyruvate kinase
ABZ99811	LEPBI_II0278	-0.8262439029	4.6417818559	0.0048981636	NA	hypothetical protein
