# Supplementary material for: Transcriptome Sequencing Reveals Wide Expression Reprogramming of Basal and Unknown Genes in Leptospira biflexa Biofilms
Source: mSphere. 2016 Apr 6;1(2):e00042-16. doi: 10.1128/mSphere.00042-16 (PMC4863578; doi:10.1128/mSphere.00042-16)
Supplement: Table S6 [file sph002162059st8.pdf]

Table S6. Expression of predicted sRNAs.

Chromosome	Start	End	Length	Strand	Description	Identification method	Active	Differentially expressed (logFC)			
								BvsP_48	BvsP_120	B_120vs48	P_120vs48
chrI	2043554	2043715	162	-	Lysine	Darn	No	-	-	-	-
chrI	2806361	2806534	174	+	Lysine	Darn	No	-	-	-	-
chrI	246415	246456	42	-	SAM	Darn	No	-	-	-	-
chrI	648107	648163	57	-	RtT	YASS/Rfam_10.0_seed	No	-	-	-	-
chrI	2380114	2380168	55	-	RtT	YASS/Rfam_10.0_seed	No	-	-	-	-
chrI	2468926	2469054	129	-	LR-PK1	YASS/Rfam_10.0_seed	No	-	-	-	-
chrI	414383	414453	71	+	CAESAR	INFERNAL	Yes	-	-	-	-
chrI	796163	796191	29	+	RprA	ERPIN	Yes	-	-	-	-
chrI	877669	877699	31	-	RprA	ERPIN	Yes	-	-0,70	-	1,3
chrI	3473660	3473689	30	-	RprA	ERPIN	Yes	-	-	-	-
chrI	906252	906279	28	-	SraE/RygA/RygB_family	ERPIN	No	-	-	-	-
chrI	1628886	1628914	29	-	SraE/RygA/RygB_family	ERPIN	No	-	-	-	-
chrI	2559248	2559275	28	-	RyeE	ERPIN	Yes	-	-	-	-
chrI	343749	343817	69	-	SL2	ERPIN	No	-	-	-	-
chrI	1155497	1155531	35	-	Threonine_operon_leader	ERPIN	Yes	-	-	-	-
chrI	906256	906281	26	+	Leucine_operon_leader	ERPIN	No	-	-	-	-
chrI	2407469	2407494	26	+	Leucine_operon_leader	ERPIN	Yes	-	-	-	-
chrI	906256	906281	26	-	Leucine_operon_leader	ERPIN	No	-	-	-	-
chrI	2407469	2407494	26	-	Leucine_operon_leader	ERPIN	No	-	-	-	-
chrI	2778262	2778283	22	+	Pseudomonas_sRNA_P9	ERPIN	No	-	-	-	-
chrI	3297300	3297322	23	+	Pseudomonas_sRNA_P9	ERPIN	No	-	-	-	-
chrI	429033	429133	101	+	GEMM_cis-regulatory_element	ERPIN	No	-	-	-	-
chrI	2620243	2620310	68	-	Pseudoknot_of_the_domain_G(G12)_of_23S_r	ERPIN	No	-	-	-	-
chrI	2922549	2922616	68	-	Pseudoknot_of_the_domain_G(G12)_of_23S_r	ERPIN	No	-	-	-	-
chrI	2429451	2429479	29	+	CRISPR_RNA_direct_repeat_element	ERPIN	No	-	-	-	-
chrI	3427282	3427302	21	+	CRISPR_RNA_direct_repeat_element	ERPIN	No	-	-	-	-
chrI	355359	355369	11	+	CRISPR_RNA_direct_repeat_element	ERPIN	No	-	-	-	-
chrI	809310	809320	11	+	CRISPR_RNA_direct_repeat_element	ERPIN	No	-	-	-	-
chrI	1302840	1302850	11	+	CRISPR_RNA_direct_repeat_element	ERPIN	No	-	-	-	-
chrI	1542856	1542866	11	+	CRISPR_RNA_direct_repeat_element	ERPIN	No	-	-	-	-
chrI	1913303	1913313	11	+	CRISPR_RNA_direct_repeat_element	ERPIN	No	-	-	-	-

chrI	3418241	3418251	11	+	CRISPR_RNA_direct_repeat_element	ERPIN	No	-	-	-	-
chrI	60811	60821	11	-	CRISPR_RNA_direct_repeat_element	ERPIN	No	-	-	-	-
chrI	702167	702177	11	-	CRISPR_RNA_direct_repeat_element	ERPIN	No	-	-	-	-
chrI	1100592	1100602	11	-	CRISPR_RNA_direct_repeat_element	ERPIN	No	-	-	-	-
chrI	1654039	1654049	11	-	CRISPR_RNA_direct_repeat_element	ERPIN	No	-	-	-	-
chrI	3078358	3078368	11	-	CRISPR_RNA_direct_repeat_element	ERPIN	No	-	-	-	-
chrI	3199865	3199875	11	-	CRISPR_RNA_direct_repeat_element	ERPIN	No	-	-	-	-
chrI	3456144	3456154	11	-	CRISPR_RNA_direct_repeat_element	ERPIN	No	-	-	-	-
chrI	3534691	3534701	11	-	CRISPR_RNA_direct_repeat_element	ERPIN	No	-	-	-	-
chrI	3590602	3590612	11	-	CRISPR_RNA_direct_repeat_element	ERPIN	No	-	-	-	-
chrI	1500802	1500827	26	-	isrJ_Hfq_binding	ERPIN	No	-	-	-	-
chrI	1690756	1690786	31	+	Deinococcus_Y_RNA	INFERNAL	No	-	-	-	-
chrI	2637082	2637128	47	+	EAV_LTH	INFERNAL	No	-	-	-	-
chrI	1479823	1479891	69	-	FinP	INFERNAL	Yes	-	-	-	-
chrI	1302364	1302469	106	+	GRIK4_3p_UTR	INFERNAL	No	-	-	-	-
chrI	261127	261199	73	-	Gurken	INFERNAL	No	-	-	-	-
chrI	808656	808703	48	-	Hairpin	INFERNAL	No	-	-	-	-
chrI	2640802	2640828	27	+	IRE	INFERNAL	No	-	-	-	-
chrI	106391	106435	45	+	K10_TLS	INFERNAL	No	-	-	-	-
chrI	270271	270311	41	+	K10_TLS	INFERNAL	No	-	-	-	-
chrI	635829	635873	45	-	K10_TLS	INFERNAL	No	-	-	-	-
chrI	2342917	2342938	22	+	PSLVbeta_UPD-PK2	INFERNAL	No	-	-	-	-
chrI	2673819	2673840	22	+	PSLVbeta_UPD-PK2	INFERNAL	No	-	-	-	-
chrI	3241758	3241875	118	+	PyrR	INFERNAL	No	-	-	-	-
chrI	2775835	2775965	131	+	PyrR	INFERNAL	No	-	-	-	-
chrI	1313428	1313543	116	+	PyrR	INFERNAL	Yes	-	-	-0,44	-
chrI	2766380	2766474	95	-	PyrR	INFERNAL	No	-	-	-	-
chrI	2167599	2167685	87	-	RtT	INFERNAL	No	-	-	-	-
chrI	1445873	1445964	92	-	RtT	INFERNAL	No	-	-	-	-
chrI	2822907	2822950	44	+	S-element	INFERNAL	No	-	-	-	-
chrI	252065	252124	60	-	S-element	INFERNAL	Yes	-	-	-	-
chrI	1658079	1658175	97	-	S15	INFERNAL	No	-	-	-	-
chrI	2384773	2384794	22	-	SBRMV1_UPD-PKd	INFERNAL	No	-	-	-	-
chrI	1850854	1850921	68	-	SECIS	INFERNAL	Yes	-	-	-	-
chrI	1870392	1870470	79	-	SNORA55	INFERNAL	No	-	-	-	-

chrI	1334839	1334907	69	+	SNORD15	INFERNAL	No	-	-	-	-
chrI	957439	957526	88	-	SNORD21	INFERNAL	No	-	-	-	-
chrI	2399635	2399723	89	-	SNORD34	INFERNAL	No	-	-	-	-
chrI	2760326	2760398	73	-	SNORD59	INFERNAL	No	-	-	-	-
chrI	79478	79550	73	-	SNORD59	INFERNAL	No	-	-	-	-
chrI	1397574	1397618	45	+	SNORD70	INFERNAL	No	-	-	-	-
chrI	2682080	2682156	77	+	SNORD70	INFERNAL	No	-	-	-	-
chrI	2941931	2941996	66	+	SNORD70	INFERNAL	No	-	-	-	-
chrI	588261	588310	50	-	SNORD70	INFERNAL	No	-	-	-	-
chrI	2273136	2273213	78	-	SNORD70	INFERNAL	No	-	-	-	-
chrI	229033	229132	100	+	SNORD86	INFERNAL	No	-	-	-	-
chrI	1864077	1864141	65	+	SNORD98	INFERNAL	No	-	-	-	-
chrI	336293	336428	136	+	Telomerase-cil	INFERNAL	No	-	-	-	-
chrI	1176691	1176823	133	-	Telomerase-cil	INFERNAL	No	-	-	-	-
chrI	1201358	1201378	21	-	UPD-PKg	INFERNAL	No	-	-	-	-
chrI	1221813	1221905	93	+	bantam	INFERNAL	No	-	-	-	-
chrI	2479052	2479120	69	-	ctRNA_pGA1	INFERNAL	No	-	-	-	-
chrI	2766386	2766450	65	+	ctRNA_pND324	INFERNAL	No	-	-	-	-
chrI	1433265	1433350	86	-	ctRNA_pND324	INFERNAL	Yes	-	-	-	-
chrI	3241798	3241883	86	+	ctRNA_pT181	INFERNAL	No	-	-	-	-
chrI	906249	906321	73	+	ctRNA_pT181	INFERNAL	No	-	-	-	-
chrI	1965107	1965175	69	+	ctRNA_pT181	INFERNAL	Yes	-	-	-	-
chrI	2988832	2988905	74	+	ctRNA_pT181	INFERNAL	No	-	-	-	-
chrI	982021	982108	88	+	ctRNA_pT181	INFERNAL	No	-	-	-	-
chrI	805787	805877	91	+	ctRNA_pT181	INFERNAL	No	-	-	-	-
chrI	1512817	1512906	90	-	ctRNA_pT181	INFERNAL	Yes	-	-	-	-
chrI	2581761	2581847	87	-	ctRNA_pT181	INFERNAL	No	-	-	-	-
chrI	551851	551953	103	-	ctRNA_pT181	INFERNAL	No	-	-	-	-
chrI	3324073	3324132	60	+	nos_TCE	INFERNAL	No	-	-	-	-
chrI	2465099	2465153	55	+	sR11	INFERNAL	No	-	-	-	-
chrI	1495918	1495968	51	+	sR11	INFERNAL	No	-	-	-	-
chrI	398673	398733	61	+	sR15	INFERNAL	No	-	-	-	-
chrI	3394232	3394298	67	+	sR15	INFERNAL	No	-	-	-	-
chrI	1708862	1708926	65	+	sR15	INFERNAL	No	-	-	-	-
chrI	2953642	2953699	58	+	sR2	INFERNAL	No	-	-	-	-

chrI	1309456	1309505	50	+	sR2	INFERNAL	No	-	-	-	-
chrI	63511	63573	63	+	sR21	INFERNAL	No	-	-	-	-
chrI	270442	270497	56	+	sR33	INFERNAL	Yes	-	-	-	-
chrI	1533334	1533388	55	-	sR48	INFERNAL	No	-	-	-	-
chrI	1556386	1556456	71	-	sn2841	INFERNAL	No	-	-	-	-
chrI	2398082	2398209	128	+	snR13	INFERNAL	No	-	-	-	-
chrI	1237121	1237215	95	+	snR58	INFERNAL	Yes	-	-	-	-
chrI	1239485	1239573	89	+	snR62	INFERNAL	No	-	-	-	-
chrI	2482774	2482842	69	+	snR62	INFERNAL	No	-	-	-	-
chrI	174215	174355	141	-	snoJ26	INFERNAL	No	-	-	-	-
chrI	187992	188057	66	-	snoJ26	INFERNAL	No	-	-	-	-
chrI	801325	801413	89	+	snoM1	INFERNAL	Yes	-	-	-	-
chrI	752727	752774	48	-	snoMe28S-Am982	INFERNAL	No	-	-	-	-
chrI	3470065	3470145	81	+	snoR11	INFERNAL	Yes	-	-	1,63	-
chrI	145230	145314	85	-	snoR11	INFERNAL	No	-	-	-	-
chrI	1709892	1709965	74	+	snoR12	INFERNAL	No	-	-	-	-
chrI	2561073	2561149	77	-	snoR160	INFERNAL	No	-	-	-	-
chrI	413407	413472	66	-	snoR28	INFERNAL	No	-	-	-	-
chrI	479500	479591	92	+	snoR30	INFERNAL	No	-	-	-	-
chrI	332258	332294	37	-	snoR31	INFERNAL	No	-	-	-	-
chrI	2656210	2656306	97	-	snoR31	INFERNAL	No	-	-	-	-
chrI	218105	218191	87	-	snoR43	INFERNAL	No	-	-	-	-
chrI	2839461	2839507	47	-	snoR4a	INFERNAL	No	-	-	-	-
chrI	2783973	2784014	42	+	snoR53Y	INFERNAL	No	-	-	-	-
chrI	1003769	1003806	38	+	snoR53Y	INFERNAL	Yes	-	-	-	-
chrI	1831833	1831900	68	+	snoR53Y	INFERNAL	No	-	-	-	-
chrI	469141	469207	67	-	snoR53Y	INFERNAL	No	-	-	-	-
chrI	365016	365081	66	+	snoR64a	INFERNAL	No	-	-	-	-
chrI	2895532	2895594	63	+	snoR72	INFERNAL	No	-	-	-	-
chrI	707984	708037	54	+	snoR72	INFERNAL	No	-	-	-	-
chrI	427418	427513	96	+	snoR98	INFERNAL	Yes	-	-	-1,29	-
chrI	2991310	2991387	78	-	snoR99	INFERNAL	No	-	-	-	-
chrI	121064	121120	57	-	snoU35	INFERNAL	No	-	-	-	-
chrI	377715	377779	65	-	snoU43C	INFERNAL	No	-	-	-	-
chrI	1180607	1180686	80	-	snoU83D	INFERNAL	No	-	-	-	-

chrI	1616861	1616909	49	+	snoZ118	INFERNAL	No	-	-	-	-
chrI	575330	575431	102	-	snoZ118	INFERNAL	No	-	-	-	-
chrI	608866	608946	81	-	snoZ122	INFERNAL	No	-	-	-	-
chrI	3168029	3168092	64	+	snoZ13_snr52	INFERNAL	No	-	-	-	-
chrI	2409314	2409373	60	+	snoZ13_snr52	INFERNAL	Yes	-	-	-0,95	-
chrI	60462	60556	95	-	snoZ13_snr52	INFERNAL	No	-	-	-	-
chrI	30984	31055	72	-	snoZ155	INFERNAL	No	-	-	-	-
chrI	705749	705803	55	-	snoZ159	INFERNAL	No	-	-	-	-
chrI	3041968	3042044	77	-	snoZ159	INFERNAL	No	-	-	-	-
chrI	3056776	3056852	77	+	snoZ165	INFERNAL	No	-	-	-	-
chrI	1915576	1915656	81	-	snoZ168	INFERNAL	No	-	-	-	-
chrI	2759895	2759935	41	+	snoZ169	INFERNAL	No	-	-	-	-
chrI	2564043	2564092	50	+	snoZ175	INFERNAL	No	-	-	-	-
chrI	2014686	2014726	41	-	snoZ175	INFERNAL	No	-	-	-	-
chrI	3438373	3438459	87	+	snoZ182	INFERNAL	No	-	-	-	-
chrI	350208	350281	74	+	snoZ196	INFERNAL	No	-	-	-	-
chrI	635480	635529	50	-	snoZ196	INFERNAL	No	-	-	-	-
chrI	841244	841333	90	+	snoZ223	INFERNAL	No	-	-	-	-
chrI	1668509	1668570	62	+	snoZ223	INFERNAL	No	-	-	-	-
chrI	2768448	2768530	83	-	snoZ223	INFERNAL	No	-	-	-	-
chrI	1445310	1445399	90	-	snoZ247	INFERNAL	No	-	-	-	-
chrI	729296	729411	116	-	snoZ5	INFERNAL	No	-	-	-	-
chrI	1520269	1520323	55	+	snoZ7	INFERNAL	No	-	-	-	-
chrI	3347127	3347200	74	+	suhB	INFERNAL	Yes	-	-	-	-
chrI	3220259	3220347	89	-	suhB	INFERNAL	Yes	-	-	-	-
chrI	2963232	2963335	104	-	sxy	INFERNAL	No	-	-	-	-
chrI	3585895	3585994	100	-	sxy	INFERNAL	Yes	-	-	-	-
chrI	2373341	2373453	113	-	TPP	combine:BLAST/Rfam_10.0_seed+INFERNAL+YASS/Rfam_10.0_seed	No	-	-	-	-
chrI	1761250	1761315	66	+	RtT	combine:BLAST/Rfam_10.0_seed+YASS/Rfam_10.0_seed	No	-	-	-	-
chrI	1222604	1222952	349	+	RNaseP	combine:BLAST/Rfam_10.0_seed+Darn+INFERNAL+YASS/Rfam_10.0_seed	Yes	-	-	-	-
chrI	132665	133015	351	-	tmRNA	combine:INFERNAL+YASS/Rfam_10.0_seed	No	-	-	-	-
chrI	2620243	2620351	109	-	PK-G12rRNA	combine:BLAST/Rfam_10.0_seed+YASS/Rfam_10.0_seed	No	-	-	-	-
chrI	2922549	2922657	109	-	PK-G12rRNA	combine:BLAST/Rfam_10.0_seed+YASS/Rfam_10.0_seed	No	-	-	-	-
chrII	101839	101849	11	+	CRISPR_RNA_direct_repeat_element	ERPIN	No	-	-	-	-
chrII	114356	114366	11	+	CRISPR_RNA_direct_repeat_element	ERPIN	No	-	-	-	-

chrII	73622	73695	74	+	GRIK4_3p_UTR	INFERNAL	No	-	-	-	-
chrII	274101	274193	93	+	SL1	INFERNAL	Yes	-0,44	0,58	0,43	-0,58
chrII	47848	47910	63	-	SNORD37	INFERNAL	No	-	-	-	-
chrII	10667	10765	99	+	Telomerase-cil	INFERNAL	No	-	-	-	-
chrII	102287	102318	32	-	UPD-PKib	INFERNAL	Yes	-	-	-	-
chrII	179514	179599	86	+	snoZ102_R77	INFERNAL	Yes	-	-	-	-
chrII	139385	139459	75	+	snoZ155	INFERNAL	Yes	-	-	-	-
chrII	220582	220642	61	-	sxy	INFERNAL	Yes	-	-	-	-
p74	23574	23672	99	-	snoJ26	INFERNAL	No	-	-	-	-
p74	57812	57872	61	-	snoR53Y	INFERNAL	No	-	-	-	-
