# Supplementary material for: Transcriptome Sequencing Reveals Wide Expression Reprogramming of Basal and Unknown Genes in Leptospira biflexa Biofilms
Source: mSphere. 2016 Apr 6;1(2):e00042-16. doi: 10.1128/mSphere.00042-16 (PMC4863578; doi:10.1128/mSphere.00042-16)
Supplement: Table S7 [file sph002162059st9.pdf]

Table S7. Annotation based on manual and structural curation.

Protein	Gene	Annotation
ABZ96159	LEPBI_I0012	DNA polymerase III, delta subunit
ABZ96159	LEPBI_I0012	DNA polymerase III, delta subunit
ABZ96192	LEPBI_I0045	Methyltransferase
ABZ96198	LEPBI_I0051	Outer membrane peptidase
ABZ96213	LEPBI_I0066	Putative transcriptional repressor
ABZ96245	LEPBI_I0098	Surface layer protein
ABZ96246	LEPBI_I0099	Phosphodiesterase- biofilm
ABZ96252	LEPBI_I0105	UDP-glucose 4-epimerase
ABZ96257	LEPBI_I0110	Lipid binding protein
ABZ96259	LEPBI_I0112	Beta propeller fold
ABZ96303	LEPBI_I0158	Signal recognition particle (ARN)
ABZ96320	LEPBI_I0175	Queuosine biosynthesis protein
ABZ96336	LEPBI_I0191	Lipid binding protein
ABZ96371	LEPBI_I0226	Putative thioesterase
ABZ96391	LEPBI_I0246	Alpha-beta hydrolase
ABZ96392	LEPBI_I0247	Thioredoxin fold; peroxiredoxin
ABZ96400	LEPBI_I0255	Outer membrane protein; cell-WALL attachment
ABZ96405	LEPBI_I0260	Sensor histidine kinase
ABZ96420	LEPBI_I0276	Ribonuclease-like protein
ABZ96445	LEPBI_I0301	alpha-beta-barrel
ABZ96461	LEPBI_I0317	tetratricopeptide repeats (TPR) containing protein
ABZ96511	LEPBI_I0368	DINB/YFIT-like putative metalloenzyme fold
ABZ96523	LEPBI_I0381	Alpha beta topology; metal transport
ABZ96546	LEPBI_I0404	Metalloendopeptidase
ABZ96563	LEPBI_I0421	Tautomerase/dehalogenase
ABZ96591	LEPBI_I0452	Chaperone protein
ABZ96627	LEPBI_I0489	Response regulator aspartate phosphatase
ABZ96669	LEPBI_I0531	Metal-binding protein
ABZ96675	LEPBI_I0537	Permease YjgP/YjgQ family
ABZ96784	LEPBI_I0651	Putative periplasmic protease
ABZ96878	LEPBI_I0746	Aminoglycoside phosphotransferase
ABZ96899	LEPBI_I0768	Transmembrane oligosaccharyl transferase
ABZ96912	LEPBI_I0782	Alpha/beta hydrolase family
ABZ96919	LEPBI_I0789	Citrate lyase; beta barrel
ABZ96937	LEPBI_I0809	Outer membrane lipoprotein
ABZ96983	LEPBI_I0856	Multidrug resistance protein outer membrane protein
ABZ96985	LEPBI_I0858	Lipase
ABZ96986	LEPBI_I0859	Lipase; alpha-beta hydrolase fold
ABZ96987	LEPBI_I0860	Lipase
ABZ97010	LEPBI_I0885	Lipase chaperone
ABZ97081	LEPBI_I0957	DNA repair
ABZ97112	LEPBI_I0988	Conserved lipoprotein LPS cell-WALL
ABZ97202	LEPBI_I1081	Diguanylate cyclase; biofilm
ABZ97256	LEPBI_I1139	flavoprotein- FAD/NADP-binding rossmann fold
ABZ97362	LEPBI_I1250	Glutamine cyclotransferase
ABZ97437	LEPBI_I1327	Anti sigma factor FlgM
ABZ97464	LEPBI_I1354	Transcriptional regulator
ABZ97465	LEPBI_I1355	Thioesterase superfamily
ABZ97541	LEPBI_I1433	Phospholipase C; membrane/calcium binding
ABZ97630	LEPBI_I1523	RNA binding protein

ABZ97791	LEPBI_I1684	PilO protein
ABZ97830	LEPBI_I1724	Alpha/beta hydrolase fold - probable esterase
ABZ97855	LEPBI_I1749	Alpha-beta sandwich; hydrolase
ABZ97888	LEPBI_I1782	Lojap-like protein
ABZ97911	LEPBI_I1805	Probable two-component response regulator
ABZ97925	LEPBI_I1819	AdoMet dependent methyltransferase
ABZ97928	LEPBI_I1822	Cell-binding factor 2; SURA-like, chaperone
ABZ97936	LEPBI_I1830	lipid binding protein
ABZ97950	LEPBI_I1844	Putative ABC transporter permease
ABZ98095	LEPBI_I1993	Transmembrane oligosaccharyl transferase
ABZ98172	LEPBI_I2070	Inner membrane Glycoside hydrolase family 9
ABZ98229	LEPBI_I2127	Alpha/beta hydrolase
ABZ98239	LEPBI_I2137	SAM-dependent methyltransferase
ABZ98276	LEPBI_I2177	Probable surface protein
ABZ98285	LEPBI_I2186	Intramembrane protease
ABZ98312	LEPBI_I2214	probable outer membrane protein
ABZ98335	LEPBI_I2239	Transcriptional repressor
ABZ98404	LEPBI_I2309	periplasmic antiviral protein
ABZ98407	LEPBI_I2312	Metal ION transporter; CBS domain
ABZ98443	LEPBI_I2352	Sensor histidine kinase
ABZ98462	LEPBI_I2371	Plasmid partition protein
ABZ98474	LEPBI_I2384	LEMA protein; bromodomain-like fold
ABZ98487	LEPBI_I2397	Coiled-coil; cell division
ABZ98603	LEPBI_I2518	Phospho-lipase
ABZ98607	LEPBI_I2523	TonB protein; beta-hairpin; transporter
ABZ98611	LEPBI_I2527	TCS - Sensor Histidine Kinase
ABZ98614	LEPBI_I2530	DNA replication and repair
ABZ98615	LEPBI_I2531	ParB/Sulfiredoxin fold
ABZ98642	LEPBI_I2561	Putative nucleotide-diphospho-sugar transferase
ABZ98673	LEPBI_I2594	Outer membrane assembly lipoprotein YFIO
ABZ98749	LEPBI_I2671	Chondroitin ABC lyase
ABZ98771	LEPBI_I2693	Putative RNA polymerase
ABZ98783	LEPBI_I2705	Carboxyl methyltransferase; membrane protein
ABZ98788	LEPBI_I2710	Histone fold protein
ABZ98895	LEPBI_I2820	Transcriptional regulator
ABZ98899	LEPBI_I2824	Choline-binding protein
ABZ98900	LEPBI_I2825	Periplasmic/cell wall glycoside hydrolase
ABZ98902	LEPBI_I2828	Antibiotic resistance
ABZ98905	LEPBI_I2834	Acetyltransferase
ABZ98945	LEPBI_I2876	Diguanylate cyclase; zinc sensor; biofilm
ABZ98975	LEPBI_I2906	DNA double-strand break repair
ABZ99004	LEPBI_I2935	Transcriptional regulator
ABZ99033	LEPBI_I2965	glycosyl transferase
ABZ99054	LEPBI_I2987	DNA-directed RNA polymerase subunit alpha
ABZ99113	LEPBI_I3047	Sensor-type histidine kinase
ABZ99214	LEPBI_I3149	Outer membrane; OMPA-like fold cell-WALL attachment
ABZ99279	LEPBI_I3214	Chaperone
ABZ99355	LEPBI_I3290	Intramembrane protease
ABZ99363	LEPBI_I3298	Outer membrane phosphate-porin
ABZ99399	LEPBI_I3335	Zinc peptidase; alpha/beta barrel
ABZ99412	LEPBI_I3348	Pyrrolo-quinoline quinone
ABZ99413	LEPBI_I3349	Sensor histidine kinase
ABZ99423	LEPBI_I3359	Ankyrin repeat family protein

ABZ99425	LEPBI_I3361	Ankyrin repeat family protein
ABZ99435	LEPBI_I3371	Glutathionylspermidine synthase
ABZ99483	LEPBI_I3422	Transcriptional regulator; biofilm
ABZ99514	LEPBI_I3454	DTDP sugar isomerase
ABZ99552	LEPBI_II0014	Membrane protein
ABZ99561	LEPBI_II0023	Probable protease HTPX homolog; heat shock protein
ABZ99570	LEPBI_II0032	Putative signal transduction protein
ABZ99573	LEPBI_II0035	ATP-NAD kinase
ABZ99577	LEPBI_II0039	TETR-family transcriptional regulator
ABZ99578	LEPBI_II0040	Diacylglycerol kinase
ABZ99603	LEPBI_II0065	RNA binding protein
ABZ99608	LEPBI_II0070	toxin-like protein
ABZ99625	LEPBI_II0088	PilZ domain
ABZ99636	LEPBI_II0100	Multidrug transporter
ABZ99637	LEPBI_II0101	RNA polymerase sigma factor
ABZ99645	LEPBI_II0109	Probable metalloproteinase
ABZ99673	LEPBI_II0138	Viral-like DNA integrase
ABZ99676	LEPBI_II0141	Membrane protein; signal transduction
ABZ99684	LEPBI_II0149	Phospholipase/carboxylesterase
ABZ99695	LEPBI_II0160	Methyl-accepting chemotaxis protein
ABZ99709	LEPBI_II0174	Transmembrane Cation efflux system protein
ABZ99725	LEPBI_II0192	Glyoxalase/bleomycin resistance protein/dioxygenase
ABZ99743	LEPBI_II0210	Carboxyl methyltransferase; membrane protein
ABZ99753	LEPBI_II0220	Porin/ outer membrane
ABZ99759	LEPBI_II0226	Cell WALL hydrolase
ABZ99768	LEPBI_II0235	Sulfate permease family protein
ABZ99781	LEPBI_II0248	Chaperone
ABZ99783	LEPBI_II0250	Putative lipoprotein
ABZ99795	LEPBI_II0262	sialic acid metabolism- kelch repeat- beta-propeller
ABZ99796	LEPBI_II0263	Sialic acid metabolism; kelch repeat
ABZ99804	LEPBI_II0271	Nitrogen regulatory protein P-II
ABZ99809	LEPBI_II0276	Lipase/acylhydrolase
ABZ99820	LEPBI_p0006	Putative thioesterase
ABZ99823	LEPBI_p0009	Alpha-beta protein
ABZ99839	LEPBI_p0025	Pyrrolo-quinoline quinone
ABZ99844	LEPBI_p0030	Cysteine peptidase
ABZ99846	LEPBI_p0032	Catabolism of external DNA
ABZ99854	LEPBI_p0042	Alpha-beta protein
ABZ99857	LEPBI_p0045	Cation efflux system protein
ABZ99861	LEPBI_p0049	Prevent HOST death protein
ABZ99862	LEPBI_p0050	Putative ribonuclease
